# Positional bias in variant calls against draft reference assemblies

**DOI:** 10.1186/s12864-017-3637-2

**Published:** 2017-03-28

**Authors:** Roman V. Briskine, Kentaro K. Shimizu

**Affiliations:** 1 Department of Evolutionary Biology and Environmental Studies, University of Zurich, Winterthurerstrasse 190, Zurich, CH-8057 Switzerland; 2 Functional Genomics Center Zurich, Winterthurerstrasse 190, Zurich, CH-8057 Switzerland; 3 Kihara Institute for Biological Research, Yokohama City University, 641-12 Maioka, Totsuka-ward, Yokohama, 244-0813 Japan

**Keywords:** Reseqencing, Variants, Polymorphisms, SNPs, Positional bias, Draft reference genome, Repetitive elements

## Abstract

**Background:**

Whole genome resequencing projects may implement variant calling using draft reference genomes assembled de novo from short-read libraries. Despite lower quality of such assemblies, they allowed researchers to extend a wide range of population genetic and genome-wide association analyses to non-model species. As the variant calling pipelines are complex and involve many software packages, it is important to understand inherent biases and limitations at each step of the analysis.

**Results:**

In this article, we report a positional bias present in variant calling performed against draft reference assemblies constructed from de Bruijn or string overlap graphs. We assessed how frequently variants appeared at each position counted from ends of a contig or scaffold sequence, and discovered unexpectedly high number of variants at the positions related to the length of either k-mers or reads used for the assembly. We detected the bias in both publicly available draft assemblies from Assemblathon 2 competition as well as in the assemblies we generated from our simulated short-read data. Simulations confirmed that the bias causing variants are predominantly false positives induced by reads from spatially distant repeated sequences. The bias is particularly strong in contig assemblies. Scaffolding does not eliminate the bias but tends to mitigate it because of the changes in variants’ relative positions and alterations in read alignments. The bias can be effectively reduced by filtering out the variants that reside in repetitive elements.

**Conclusions:**

Draft genome sequences generated by several popular assemblers appear to be susceptible to the positional bias potentially affecting many resequencing projects in non-model species. The bias is inherent to the assembly algorithms and arises from their particular handling of repeated sequences. It is recommended to reduce the bias by filtering especially if higher-quality genome assembly cannot be achieved. Our findings can help other researchers to improve the quality of their variant data sets and reduce artefactual findings in downstream analyses.

**Electronic supplementary material:**

The online version of this article (doi:10.1186/s12864-017-3637-2) contains supplementary material, which is available to authorized users.

## Background

Plummeting cost of high-throughput sequencing (HTS) allowed population geneticists to analyse hundreds of individuals on the whole genome level (e.g. [1–3]). Moreover, the researchers are no longer limited to the model species as draft genome sequences can be assembled de novo from HTS data (e.g. [4, 5]). The quality of these draft genomes is generally lower than that of the traditionally sequenced genomes [6] but they are still considered adequate for various types of analysis in population genetics as well as genome-wide association studies.

The core algorithm of a modern genome assembler usually implements either a de Bruijn graph [7] or a string graph [8]. Both approaches involve constructing a graph based on sequence overlaps and finding the optimal path through the graph. Such path would correspond to a contiguous assembled sequence known as contig. In a string graph, vertices are represented by reads while a sufficiently long overlap between two reads forms an edge. To construct a de Bruijn graph, each read is split into all possible sequences of length *k* (k-mers). The k-mers form vertices of the graph while overlaps between k-mers that are *k*−1 bases long become edges. Among the assemblers that implement the string graph approach are SGA [9] and SAGE [10] while de Bruijn graphs are incorporated into ABySS [11], Meraculous [12], ALLPATHS-LG [13], and SOAPdenovo2 [14] among many others.

Pipeline for genome assembly normally includes a rigorous error correction of the reads, which can be done either before the assembly with another tool or during the assembly by the assembler itself. Contig assembly may be followed by scaffolding whereby mate-pair reads that map to two different contigs are used to splice these contigs together into a scaffold. Finally, some pipelines for genome assembly involve merging of the overlapping paired-end reads into longer sequences before the construction of de Bruijn graphs. In fact, ALLPATHS-LG requires that sufficient number of overlapping paired-end reads are present in the input data [13]. Such approach allows the selection of longer k-mer size for the de Bruijn graph construction, which in turn improves the assembly of repetitive regions [14].

Here, we report a stark pattern that appears when calling variants against assemblies generated from de Bruijn or string graphs. If paired-end reads are aligned to the assembled contigs, an unexpectedly high number of variants will be called at a certain position from the end of a contig or scaffold. Depending on the assembler’s implementation, this position matches either the k-mer length used for de Bruijn graph construction or the length of reads used in string overlap graphs. Our analyses suggests that the bias is caused by repeated sequences that cannot be successfully resolved by assemblers. While scaffolding mitigates the bias, it does not remove the bias completely and variants are still more likely to appear at the same relative position within contigs incorporated into scaffolds. The most effective approach to attenuate the bias is to remove all variants present in repetitive elements. Since the bias-causing variants are mostly false positives, the bias may have serious implications on downstream analyses performed in resequencing projects [15, 16].

## Methods

### Whole genome assemblies

A subset of *Maylandia zebra* (fish) and *Boa constrictor* (snake) whole genome assemblies (before and after scaffolding) submitted by various teams (Table 1) as entries to the Assemblathon 2 competition [17] were downloaded from the official repository. To reduce the extent of post-processing that could potentially obscure the problem, only the teams representing the original assembler developers were chosen. Since the competitive SOAPdenovo2 assembly of the snake genome was generated using mislabelled mate-pair libraries, we downloaded the corrected version that the team made available after the competition (See Additional file 3 in Bradnam et al. [17]).

**Table 1:** List of analysed assemblies

Reads	Identifier	Assembler	Pos	k
Bcon	abyss_9C	ABySS	80	80
Bcon	merac_6C	Meraculous	71	NA
Bcon	phus_5C	Phusion	78	NA
Bcon	sga_7C	SGA	121	NA
Bcon	soap*	SOAPdenovo2	36	36
Mzeb	abyss_7C	ABySS	56	56
Mzeb	allp_6C	ALLPATHS-LG	96	96
Mzeb	soap_11E	SOAPdenovo2	46	46
Sim	allp	ALLPATHS-LG	96	96
Sim	sga_m75	SGA	100	NA
Sim	sga_m77	SGA	100	NA
Sim	soap_K69	SOAPdenovo2	70	70
Sim	soap_K71	SOAPdenovo2	72	72

This study focused on a subset of the *B. constrictor* (Bcon) and *M. zebra* (Mzeb) genome assemblies submitted by the assembler developers to the Assemblathon 2 competition. In addition, we simulated reads from *A. thaliana* chromosomes 1 and 2 (Sim) and constructed several assemblies with varying parameters. For SGA, we varied the minimum string overlap (-m 75 and -m 77 for sga_m75 and sga_m77 respectively). For SOAPdenovo2, we set the -K parameter to 69 and 71, which corresponded to k = 70 and 72 for soap_K69 and soap_K71 respectively. ‘Pos’ column shows the position (counted from ends of contigs or scaffolds) where variants occur most frequently. ‘NA’ in the ‘k’ column indicates that the choice of k was not reported and could not be determined from other sources

^*^The Bcon assembly by the SOAPdenovo2 team submitted for the competition was assembled using an incorrectly labelled library. We analysed the corrected version that was constructed after the competition [17]

For the alignment against these assemblies, we randomly selected a 400 bp insert library for *B. constrictor* (ERR234373) and 180 bp insert library for *M. zebra* (SRR077290). Each library was aligned only against the assemblies of its respective species. Both libraries were downloaded from the NCBI Sequence Read Archive.

### Simulated data set

Sequences for chromosomes 1 and 2 of *Arabidopsis thaliana* (TAIR10) were downloaded from The Arabidopsis Information Resource website [18].

To simulate the reads we used SimSeq application that aims to reproduce the biases present in normal Illumina data sets [19]. We ran the application with default parameters to simulate 15 mln 100 bp paired-end reads with the mean insert size of 180 bp and 5 mln 100 bp mate-pair reads with the mean insert size of 3 kb. Since the combined size of the chosen chromosomes is about 50 Mb, the simulated libraries yielded 60× and 20× coverage respectively.

We assembled the short reads from these libraries using ALLPATHS-LG v52293 [13] with default parameters. Henceforth, we will refer to this assembly as Sim_allp where ‘Sim’ indicates simulated libraries and ‘allp’ denotes the assembler. We also constructed two assemblies (Sim_soap_K69 and Sim_soap_K71) with SOAPdenovo2 v2.04 [14] specifying different k-mer lengths. The optimal length (*K*=69) was determined by running KmerGenie [20] for the range of lengths between 39 and 99 with the step of 2. Another length value (*K*=71) was selected as the next best length according to the KmerGenie output. In both cases, we ran SOAPdenovo-127mer with the options to resolve repeats (-R) and to drop low-frequency k-mers (-d 1). Finally, we constructed two assemblies (Sim_sga_m75 and Sim_sga_m77) using a string overlap assembler, SGA v0.10.14 [9]. For both SGA assemblies, we ran error correction with k-mer length set to 41 (-k 41) and the minimum overlap of 55 (-m 55) for the overlap command. The minimum overlap in the assemble command was set to either 75 or 77 (-m 75 or -m 77). Subsequent scaffolding was performed using default parameters as described in the SGA documentation.

To investigate variant calling for resequencing analysis, we downloaded a short-insert library (SRX144851) of *A. thaliana* Bs-1 genotype from DNA Data Bank of Japan (DDBJ). The reads were sequenced with Illumina HiSeq 2000 and have the insert size of 202 bp with 101 bp read length [21].

### Variant calling

Variants were called with GATK v3.4-0 [22] following the established best practices [23, 24]. Briefly, the corresponding short insert library was aligned against the assembled sequences using BWA v0.7.12 [25]. After marking the duplicates, the reads were locally realigned around insertions/deletions (indels) and variants were called with HaplotypeCaller. The obtained raw calls were filtered using the criteria recommended for cases when variant calibration was not possible (Table 2).

**Table 2:** SNP statistics reported by GATK and thresholds used for filtering

Abbreviation	Rel	Threshold	Full name
QD	<	2.0	Quality by Depth
MQ	<	40.0	Root mean square of Mapping Quality
MQRankSum	<	−12.5	Mapping Quality Rank Sum test
FS	>	60.0	Fisher’s exact test for Strand bias
SOR	>	4.0	Strand bias Odds Ratio
ReadPosRankSum	<	−8.0	Read Position Rank Sum test
DP	>	200.0	Depth of Coverage
GQ	<	20.0	Genotype Quality

SNPs with statistics above or below (Rel) the corresponding threshold were removed from consideration. For detailed description of these statistics and justification for the threshold selection, see Van der Auwera et al. [24] and GATK documentation at https://www.broadinstitute.org/gatk/

To make sure that variants in the regions with excessively high coverage do not affect the results, we separately applied more restrictive coverage filters to the variants called in the fish and snake genomes. The thresholds were set to the expected coverage calculated using the Lander-Waterman equation *C*=*L* *N*/*G* [26], where *C* is the expected coverage, *L* is the read length, *N* is the number of reads, and *G* is the estimated haploid genome length. Based on the reported genome lengths of 1.6 and 1 Gb [17], the expected coverage was 5 × and 8 × for the snake and fish genomes respectively.

### Alternative read alignment and variant calling tools

To exclude the possibility that the bias was caused by the tools we used for read alignment and variant calling (BWA and GATK), we also analysed the variants detected against simulated contig assemblies with alternative tools. We ran GATK on the alignments produced by each NextGenMap [27], GSNAP [28], and Bowtie2 [29]. We accepted the default parameters for each of these read alignment applications. Since we suspected that Bowtie2 might have lower sensitivity than the other aligners, we also ran Bowtie2 with the default parameters to align Bs-1 reads against the simulated contig assemblies.

We relied on BWA alignments to test the FreeBayes [30] and Samtools mpileup [31] variant callers. We ran the multithreaded version of FreeBayes and specified the same maximum coverage threshold (200) as with GATK. Following the recommendations from the FreeBayes documentation, variant calls were subsequently filtered using a minimum quality threshold (QUAL < 20). We also used the default parameters for Samtools mpileup except for the maximum indel coverage, which we set to 200. The resultant data was processed with bcftools [31] to produce a VCF file and filter out the variants with low quality (QUAL < 20) and abnormally high coverage (DP > 200).

### Scaffold position transformation (coordinate mapping)

To transform variant scaffold positions to contig positions in ALLPATHS-LG assemblies, we employed the information from the final.summary file that ALLPATHS-LG generates by default. For each scaffold, the file reports scaffold length, list of included contigs with their respective lengths, and gap sizes. Overlapping contigs have negative gap sizes. We noticed that occasionally a scaffold in the final assembly extends beyond the length specified in the summary file. In such cases, we reported the SNPs located beyond the reported scaffold length as ‘untransformed’ because their coordinates could not be mapped to any contigs.

For the transformation of scaffold positions in SOAPdenovo2 assemblies, we parsed the file with the contigPosInscaff extension. The file is automatically generated by SOAPdenovo2 during scaffolding. For each scaffold, the file lists one or more contig entries. Each entry specifies contig id, starting position within scaffold (origin 0), contig orientation, and ending position within scaffold. We also used the contig length information from the file with contig extension because SOAPdenovo2 often inserted gaps between overlapping contigs in a scaffold. Such a gap would effectively split one of the contigs into two parts making it impossible to derive a transformation map exclusively from the contigPosInscaff file.

Our scaffold coordinate transformation would produce two contig coordinates if a variant is located in a scaffold region where two contigs overlap. We chose this approach because such scaffold variants in principle should have two corresponding contig variants, one on each of the overlapping contigs.

### Identification and filtering of repetitive elements

We identified repetitive elements and low complexity sequences in the simulated assemblies using RepeatMasker v4.0.5 [32] with the library version 20140131 [33], NCBI search engine, and ‘viridiplantae’ species filter. To calculate the number of position *k* SNPs appearing in repetitive elements, we checked the SNP coordinates against the repetitive sequence ranges reported by RepeatMasker. If a repetitive element spanned position *k* at both ends of a contig and contained two position *k* SNPs (one at each end), we counted it as a single occurrence, i.e. for each repetitive element sequence the count was either 0 or 1. To adjust for family frequency, we divided the number of position *k* SNPs appearing within that family by the total number of the family sequences present in the assembly. SNP filtering process entailed the removal of all variants located within any of the identified repetitive elements or low complexity sequences.

## Results

### Positional bias in variant distribution within contigs

In our analysis, we used the publicly available data from the Assemblathon 2 competition [17]. For each of the two analysed species, we randomly selected a single short-insert library among those provided to the teams for assembly and aligned the reads against each of the chosen contig assemblies submitted for the competition. In each case, both the aligned reads and the assembly came from the same individual (species). Hence, any variant calls would be false positives and the distribution of their positions within contigs should be approximately uniform.

**Fig. 1:**
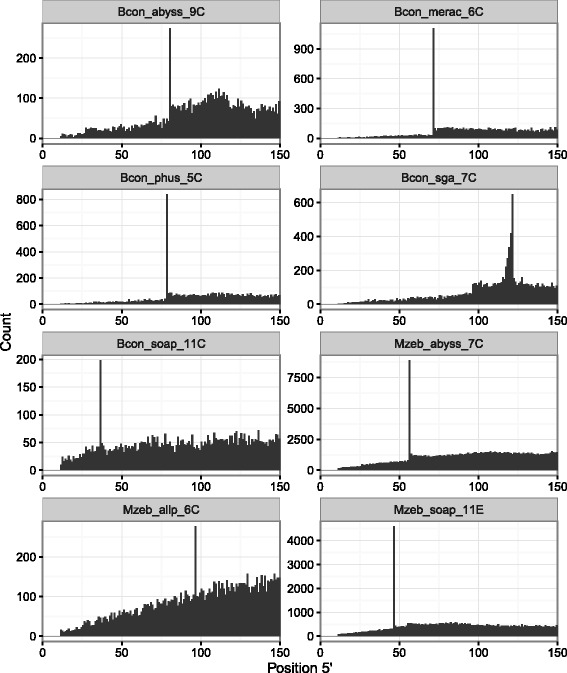
Distribution of SNP positions at the 5^′^ end of contigs. The analysis includes a subset of five *B. constrictor* and three *M. zebra* assemblies submitted to Assemblathon 2 [17]. The description of assembly identifiers is given in Table 1

After calling the variants, we calculated how frequently they appeared at each position counted from both ends of contigs (Table 1). Frequencies were estimated separately for single nucleotide polymorphisms (SNPs) and insertions/deletions (indels). All of the tested assemblies showed positional bias in the distribution of variant calls (Fig. 1; Additional file 1: Figures S1–S3). For some assemblies the bias was evident in the distribution of both SNPs and indels while others exhibited only SNP distribution bias. Since indels are typically less frequent, more difficult to call and, therefore, less reliable than SNPs, we will focus on the SNP distribution bias.

In addition to the Assemblathon 2 entries, we simulated 180 bp paired-end library and 3 kb mate-pair library using chromosomes 1 and 2 of *Arabidopsis thaliana*. We assembled the simulated paired-end library into contigs separately with several assemblers. We called variants and analysed the results using the same approach as with Assemblathon 2 data. The simulated data set provided several advantages. First, it excluded the possibility that aligning additional paired-end libraries used for assembly would affect variant calling. Assemblathon 2 teams had access to several paired-end libraries while we only aligned a single one to call variants against those assemblies. The simulated data set contained only a single short-insert paired-end library, which was subsequently aligned to the de novo assemblies. Second, we knew the exact origin of each simulated read, which helped us explain why some variants were called. Third, available HTS data for a different *A. thaliana* genotype enabled us to explore the effects on variant calling for resequencing analysis. Finally, we were able to run more analyses because of the smaller data set size.

In the literature describing de Bruijn graph approaches to assembly, k-mer length may refer to either the length of sequences at graph vertices [11, 34] or the length of sequence overlaps at graph edges [9, 14]. To avoid the confusion, we will use the first definition and denote such length as *k*. The length of sequence overlaps at graph edges will be denoted as *K*, i.e. *K*=*k*−1 for de Bruijn graphs.

**Fig. 2:**
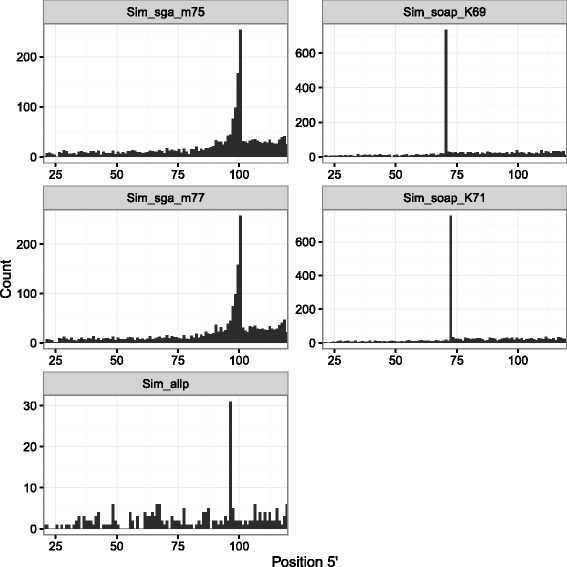
Distribution of SNP positions at the 5^′^ end of contigs in the simulated data set. The description of assembly identifiers is given in Table 1

Only three teams (ABySS, ALLPATHS-LG, and SOAPdenovo2) reported k-mer lengths used for assembly (See Additional file 3 in Bradnam et al. [17]). In all cases, the position where SNPs occurred most frequently matched the reported k-mer length (Table 1). This was independent of the tool and the actual *k* value used in the assembly (ABySS and SOAPdenovo2 teams each specified different *k* values for their respective *B. constrictor* and *M. zebra* assemblies). When assembling our simulated paired-end reads with SOAPdenovo2, we changed the *K* configuration parameter from -K 69 to -K 71 and the most frequent SNP position shifted from 70 to 72 (Fig. 2; Additional file 1: Figure S4).

Among the assemblers we analysed, SGA [9] was based on string graphs rather than de Bruijn graphs. In this assembler, the parameter equivalent to the k-mer length would be the string overlap length used for graph construction. It can be specified as -m parameter for the assemble command. Changing this parameter did not cause a shift in the peak position (Fig. 2; Additional file 1: Figure S4). Instead, the peak position was linked to read lengths. For the Assemblathon 2 entry and the simulated data sets, the read lengths were 121 and 100 bp respectively. Both values matched the most frequent SNP position of their respective assembly (Table 1). Hereafter, we will use the read length to establish the *k* position in SGA assemblies.

To make sure that the positional bias is not limited to the very short contigs with potentially poor quality, we removed all contigs shorter than 500 bp and repeated the analysis. The bias was still clearly visible in all cases (Additional file 1: Figures S5–S6) indicating that such filtering is not effective for bias reduction.

To remove the variants called in the regions with abnormally high coverage, we used the 200 × threshold (DP in Table 2). The value was higher than the expected coverage calculated with Lander-Waterman equation [26]. However, the bias was still apparent even among the variants with coverage that did not exceed the expected levels (Additional file 1: Figures S7–S8).

We also compared the distributions of various quality statistics reported by GATK (Table 2) for SNPs at position *k* to those reported for SNPs at other positions, and found them very similar (Additional file 1: Figures S9–S16). While the differences might be statistically significant due to large sample size, the effect size is minimal and considerable overlap between distributions makes SNP discrimination unfeasible. The only statistic that could be possibly used to reduce the bias without substantial effect on other SNPs is mapping quality (MQ). Even then, the results would be largely dependent on the assembler choice and the underlying data set. In particular, the distribution of MQ for SNPs in non- *k* positions had fairly long left tails in *M. zebra* (especially Mzeb_allp_6C; Additional file 1: Figure S10) while the MQ distributions for *k* and non- *k* SNPs in the simulated data set were hardly separable regardless of the assembler (Additional file 1: Figure S17).

### Scaffolding does not eliminate the positional bias

Assembly pipelines generally include a step to concatenate contigs into longer scaffold sequences based on mate-pair read alignments. We discovered that the positional bias was attenuated but still persisted in the *B. constrictor* and *M. zebra* assemblies after scaffolding (Table 3; Additional file 1: Figures S18–S19). The only assembly where the bias appeared less evident was Mzeb_allp_6C but even there the peaks at the position *k* were notably high. Similar results, including the greatest reduction of the bias appearance in the ALLPATHS-LG assembly, were observed with the simulated data set (Table 3; Additional file 1: Figures S20–S21). Removal of the short scaffolds (less than 500 bp) from the *B. constrictor* and *M. zebra* assemblies did not completely eliminate the bias either (Additional file 1: Figures S22–S23).

**Table 3:** SNP counts at position *k* in the simulated data sets

Reads	Assembler	Contig	Scaffold	Transf	Untransf	Shared	Masked
Sim	allp	57	7	21	1	18	12
Sim	sga_m75	504	463	NA	NA	NA	NA
Sim	sga_m77	513	479	NA	NA	NA	NA
Sim	soap_K69	1481	698	255	451	137	44
Sim	soap_K71	1469	769	NA	NA	NA	NA
Bs-1	allp	66	9	35	0	23	15
Bs-1	sga_m75	899	711	NA	NA	NA	NA
Bs-1	sga_m77	871	687	NA	NA	NA	NA
Bs-1	soap_K69	1916	670	365	429	210	95
Bs-1	soap_K71	1909	692	NA	NA	NA	NA

Reads column indicates the origin of aligned reads: ‘Sim’ refers to the simulated paired-end reads while ‘Bs-1’ denotes the actual *A. thaliana* Bs-1 short-insert library [21]. Contig and Scaffold columns show the number of SNPs at position *k* in the respective contig and scaffold assemblies. ‘Transf’ column shows the number of SNPs at position *k* called against scaffolds after the scaffold coordinates were transformed to contig coordinates. Only Sim_allp and Sim_soap_K69 scaffold coordinates were transformed. ‘Untransf’ column indicates the number of SNPs that failed to transform because of contig length threshold (Sim_soap_K69) or scaffold being extended beyond the length specified in the assembler’s scaffold map (Sim_allp). ‘Shared’ column reports the number of SNPs present in both Contig and Tranformed sets. ‘Masked’ column shows the SNPs that appear in Contig and Transformed but not in Scaffold because of the change in their relative positions. All counts except ‘Untransf’ are for SNPs in position *k*

There are two mechanisms that may cause the bias reduction after scaffolding. First, many contigs will be placed in the middle of scaffolds. Thus, many SNPs that were previously present near contig ends would appear in the middle of scaffold sequences as well. Consequently, some SNPs that contributed to the bias before would emerge as SNPs that do not cause the bias because they would not be in position *k* relative to scaffold ends. This mechanism makes the bias less apparent but it does not actually decrease it because corresponding SNPs still persist in the scaffold assembly (hereafter, we will refer to this phenomenon as “bias masking”). The second mechanism is triggered by alterations in read alignments. Scaffolding typically involves concatenation of overlapping contigs and gap filling between adjacent contigs. Both actions may alter read alignments in the affected regions. In particular, reads that previously caused SNP calls on individual contigs would not align sufficiently well to the same contigs or would align better elsewhere after scaffolding. Thus, the number of SNPs in both *k* and non- *k* positions may diminish. Such bias reduction would be real because it effectively eliminates the bias causing SNPs.

We can measure the effects of these two mechanisms by analysing the intersection between SNPs called before and after scaffolding. To compute the intersection, we have to transform SNP scaffold coordinates into contig coordinates using a scaffold map reported by the assembler. Bias masking occurs when a scaffold SNP from a non- *k* position appears in the position *k* after transformation and there is a contig SNP at the same position. Genuine bias reduction takes place when a contig SNP in position *k* does not have a corresponding scaffold SNP.

We transformed scaffold coordinates into contig coordinates in two assemblies constructed from the simulated reads. In the Sim_allp assembly, scaffolding reduced the number of SNPs in position *k* from 57 to 7 (Table 3). After transforming scaffold coordinates to contig coordinates, the position bias was clearly visible (Additional file 1: Figures S24–S25) as the number of scaffold SNPs in position *k* increased to 21 (Table 3). Out of those, 18 were also called against the contig assembly (shared SNPs; panel ‘Both’ in Additional file 1: Figure S26) while 3 were unique to the scaffold assembly (panel ‘Scaffold Only’ in Additional file 1: Figure S26; Additional file 1: Figure S27). Coordinates for one SNP could not be transformed because the scaffold was longer than specified in the assembler’s scaffold map. Out of the 57 contig SNPs present in position *k*, 12 (21%) emerged in the scaffold assembly at non- *k* positions relative to scaffold ends (‘Masked’ column in Table 3) while 39 (68%) SNPs completely disappeared due to altered read alignments.

In the Sim_soap_K69 assembly, scaffolding also reduced the number of position *k* SNPs (Table 3; Additional file 1: Figures S28–S29) but the underlying processes were different. A large number of SNP coordinates (451 at position *k*) were not transformed (‘Untransf’ column in Table 3) because they corresponded to locations on contigs shorter than 200 bp. Those contigs were excluded from the Sim_soap_K69 contig assembly for SNP calling while the assembler still used them for scaffolding. Therefore, the ‘untransformed’ scaffold SNPs could not overlap with the contig SNPs in principle and we ignored them when calculating the overlap between contig and transformed scaffold SNPs (Additional file 1: Figures S28–S29). We also noticed that SOAPdenovo2 tends to introduce gaps within overlapping contigs. A gap is placed between the end of one contig and the non-overlapping part of the other contig, which breaks the second contig into two non-contiguous parts. This leads to considerable changes in read alignments and consequently yields many SNPs unique to the contig assembly (Table 3; Additional file 1: Figures S28–S29). These two reasons also explain the large difference between the number of contig and coordinate-transformed scaffold SNPs (‘transf’ column) but much smaller difference between contig and scaffold SNP counts (Table 3). It also explains why the bias masking level is so low; only 44 (3%) scaffold SNPs in non- *k* positions could be matched to contig SNPs in position *k*.

### Positional bias with alternative tools

To make sure that the positional bias was not caused by one of the selected tools (BWA and GATK), we executed variant calling pipelines with alternative read alignment or variant discovery applications on the simulated contig assemblies. Running GATK with either NextGenMap [27] or GSNAP [28] still resulted in clearly visible peaks at the expected locations (Additional file 1: Figures S30–S33). When using GATK with Bowtie2 [29], the peaks were much smaller in SGA and SOAPdenovo2 assemblies and the bias was completely absent in the ALLPATHS-LG assembly (Additional file 1: Figures S34–S35). However, the number of variants at other positions was much smaller as well.

Previous reports indicated that Bowtie2 was less sensitive to sequence mismatches [35, 36] and may have higher error rates [37]. To check the sensitivity of Bowtie2 on our simulated data set, we ran the Bowtie2 – GATK pipeline using resequencing data (see the next section for additional results). Compared to the BWA – GATK pipeline, we saw a considerable reduction in the total number of variant calls (Table 4; Additional file 1: Figures S36–S37). This is in contrast to Cornish and Guda [38] who reported only a minor decrease in SNPs between similar pipelines. These results should be interpreted with caution as we do not know the actual number of errors in each case.

**Table 4:** SNPs called by GATK when using BWA or Bowtie2 (Bt2) to align Bs-1 reads against simulated contig assemblies

Assembly	Bt2 Total	Bt2 Pos k	BWA Total	BWA Pos k
Sim_allp	97,020	3	488,839	69
Sim_sga_m75	97,740	28	500,829	948
Sim_sga_m77	97,727	27	501,055	917
Sim_soap_K69	96,485	35	497,002	2016
Sim_soap_K71	96,003	44	497,486	2004

Total columns show the total number of SNPs in any position while ‘Pos k’ columns show the number of SNPs at position *k*

Variants detected by each FreeBayes [30] and Samtools mpileup [31] using BWA alignments also exhibited strong positional bias (Additional file 1: Figures S38–S41). Since Samtools skips anomalous read pairs and orphan reads by default, filtering out improperly-paired reads would not remove the positional bias.

### Positional bias is present in resequencing analysis

In the previous subsections, reads used for variant calling came from the same individual as the reads used for the reference genome assembly. Therefore, any called variants would be considered false positives. In this subsection, we investigate the alignment of reads from a different individual to the reference genome. In this case, some of the called variants should be real but there could also be false positives that would potentially manifest themselves as the positional bias described in this study. This analysis imitates the practical use of draft de novo assemblies in resequencing projects that focus on non-model species.

To verify that the positional bias would still be present in variant calling performed with reads from a different individual, we aligned a short-insert library of *A. thaliana* Bs-1 genotype [21] against our assemblies constructed from the simulated read data. The bias was present in all contig assemblies (Additional file 1: Figures S42–S43). It was also clearly visible in both Sim_sga and both Sim_soap scaffold assemblies at the expected locations (Additional file 1: Figures S44–S45). Meanwhile, the bias essentially disappeared in the Sim_allp scaffold assembly, probably because of the assembly’s high quality (Additional file 1: Figures S44–S45). As before, we transformed scaffold positions into contig positions for all scaffold SNPs and observed partial recovery of the bias in Sim_allp (Table 3; Additional file 1: Figures S46–S47). We traced a large number of SNPs on Sim_soap_K69 scaffolds to contigs shorter than 200 bp (‘Untransf’ column in Table 3). We also found that 15 and 95 SNPs (23 and 5%) from non- *k* positions in Sim_allp and Sim_soap_K69 scaffolds respectively corresponded to position *k* SNPs in the contig assemblies (bias masking). These results are consistent with those uncovered through the alignment of our simulated reads.

We tested whether the variant positions in the Bs-1 alignments overlapped with the variant positions in the simulated read alignments. If they overlap well, removing these positions may provide a useful solution for the bias reduction. Despite the partial overlap (Additional file 1: Figures S48–S49; ‘Both’ row in Additional file 1: Figures S50–S51), Sim_allp and Sim_soap_K69 data sets possessed abnormally high number of position *k* SNPs that were unique to the Bs-1 alignments (‘Bs-1’ row in Additional file 1: Figures S50–S51). These SNPs comprise the variants that would remain after the filtering of the shared SNPs. The remaining bias was particularly strong in case of Sim_soap_K69. Similar pattern appeared when positions of scaffold SNPs were transformed into contig coordinates (Additional file 1: Figures S52–S55). Even though the filtering essentially eliminated the bias from the 5^′^ end of the Sim_allp assembly (‘Bs-1’ row in Additional file 1: Figure S54), the results were not universal and other solutions would be needed.

### Considerable reduction in bias achieved by repetitive element filtering

For each assembly created from the simulated read data, we traced the origins of all reads that had primary alignments to SNPs at position *k* with the mapping quality of at least 40 and without any insertions or deletions compared to the reference (CIGAR = 100M). Such conservative filtering ensured that the analysed reads aligned well only to a single location in the assembly and the alignments were not coerced by read clipping. If all such reads had a single origin per variant, it would suggest that the corresponding variants were caused by either simulated sequencing errors or poorly aligned reads from remote genomic regions with modest similarity. If the reads had multiple origins, the variants were likely caused by highly similar repeated or homologous sequences in the genome.

Fewer than 6% of the SNPs had the selected reads coming from the same origin. It suggests that the bias is not caused by poor alignments or sequencing errors but rather by repetitive or homologous sequences. Such sequences would lead to multiple potential path extensions through de Bruijn or string graphs. The extensions would also have equally high support and may form either a junction or a so-called “bubble” in the path [11]. Further investigation is needed to determine why the assemblers tend to terminate the path extension shortly after a bubble or a junction is formed.

**Fig. 3:**
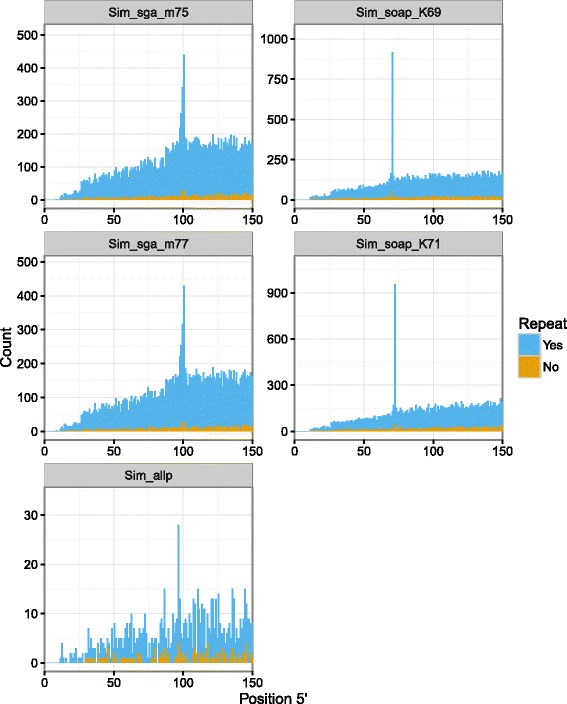
Distribution of SNP positions at the 5^′^ end of contigs in the Bs-1 data set with repetitive element annotation. Colour indicates whether the SNPs are within repetitive sequences (*blue*) or not (*orange*). SNPs were called from the Bs-1 read alignments. Repetitive elements included all sequences reported by RepeatMasker

We used RepeatMasker [32] to identify repetitive elements in the assemblies constructed from the simulated reads and discovered that the vast majority of SNPs near contig or scaffold ends were within those sequences (Fig. 3; Additional file 1: Figures S56–S66). The pattern persisted whether the SNPs were called against contigs (Additional file 1: Figures S56–S57) or scaffolds (Additional file 1: Figures S58–S61) and whether the SNPs were called from the alignment of our simulated reads (Additional file 1: Figures S56–S61) or from the alignment of the actual Bs-1 reads (Fig. 3; Additional file 1: Figures S62–S66).

In the simulated contig data set, position *k* SNPs appeared within diverse repetitive elements. The largest number of SNPs were in the DNA and long terminal repeat (LTR) families (Additional file 1: Figure S67), which had the highest representation in the *A. thaliana* genome [39]. When adjusted for family frequency, SNPs were more likely to appear in the satellites (ALLPATHS and SOAPdenovo2 assemblies) or DNA repeats (SGA assemblies). However, none of the families were strongly overrepresented.

**Fig. 4:**
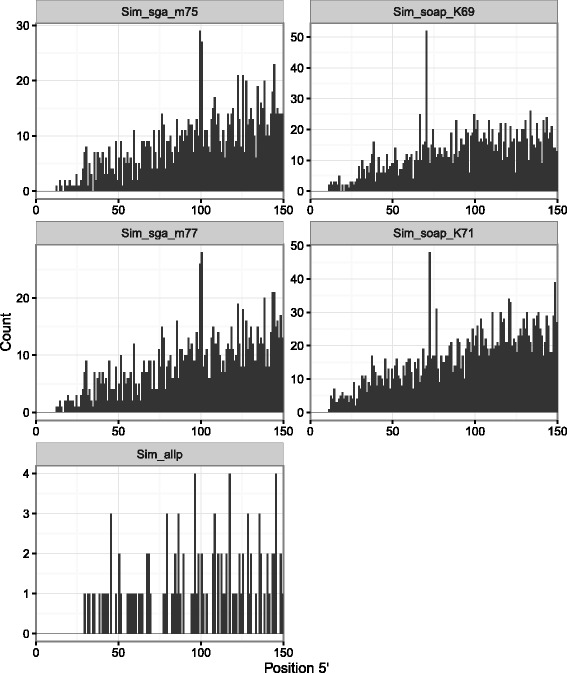
Distribution of SNP positions at the 5^′^ end of contigs in the Bs-1 data set after repetitive element filtering. SNPs were called from the Bs-1 read alignments and SNPs located in the annotated repetitive elements were removed

When the SNPs located in repetitive elements were filtered out from the set obtained through the alignment of the simulated reads, the positional bias was either completely eliminated as in the case of Sim_allp or reduced to negligible levels as in the case of Sim_soap and Sim_sga assemblies (Additional file 1: Figures S68–S73). The remaining bias might be due to unannotated repetitive elements or other homologous sequences. The same tendency was observed with the SNPs called from the alignment of the actual Bs-1 reads (Fig. 4; Additional file 1: Figures S74–S78) except that more SNPs remained overall. This is expected because the alignment of the simulated reads can only produce false positives while the alignment of Bs-1 reads should additionally yield real SNPs.

Finally, we checked whether it would be possible to reduce the bias even further by removing both SNPs in repetitive elements and SNPs produced with the simulated read alignments from the Bs-1 SNP set. Since the bias after the repetitive element filtering was already absent in Sim_allp and barely detectable in Sim_sga assemblies, we only report the results for Sim_soap_K69. After transforming scaffold coordinates to contig coordinates, there were 25 SNPs called at position *k* from Bs-1 read alignments. Out of them, 6 SNPs were also produced by the simulated read alignments (Additional file 1: Figure S79). Overall effect of the additional filtering is fairly minor (row ‘Both’ in Additional file 1: Figure S80) but the bias almost entirely disappears (row ‘Bs-1 Only’ in Additional file 1: Figure S80).

## Discussion

Resequencing projects rely on a wide range of complex applications that often need to be tuned for the best performance. To achieve high quality results, it is essential to know limitations and biases inherent to each employed application. We have shown that variants obtained from the alignment of short reads against assemblies constructed with de Bruijn or string graphs suffer from positional bias. While the degree of bias varied depending on the input data and setup, it was clearly visible in most tested configurations that encompassed several popular aligners and assemblers. If not addressed, the bias may trigger confounding effects in downstream analyses.

To confirm the bias, we designed two types of analyses. First, we called variants after aligning the reads from a short-insert library that was previously used to construct the reference assembly. In this case, all variants would be false positives. However, they would also be likely to appear when aligning reads from a different individual as long as the corresponding regions are conserved. This type of analyses was performed on both publicly available assemblies and our assemblies constructed from simulated reads. Second, we performed variant analyses with actual reads coming from a genotype different from the reference. In this case, the variants would contain a mix of true positives and false positives. Due to the limited availability of data, we only performed these analyses with the assemblies constructed from the simulated reads.

Our results suggest that the positional bias was caused by the alignment of reads from repetitive or homologous sequences that often had fewer copies included in the assembly compared to the actual genome. The most effective method to reduce the bias is to remove the variants located in repeated sequences. Such variants are very likely to be false positives even when they are not located at position *k*. Depending on the assembler configuration and input data, such filtering may either eliminate the bias completely or reduce it to almost negligible level. The remaining bias-causing SNPs probably reside in unannotated repetitive elements or other homologous sequences that have multiple copies in the genome but only a single copy in the assembly. While some of the variants could have been caused by heterozygosity in *B. constrictor* and *M. zebra* genomes, the simulated data set essentially represented a haploid individual without any heterozygous regions. Therefore, heterozygosity is unlikely to constitute a major factor in positional bias.

We also observed the positional bias while using alternative variant callers (FreeBayes and Samtools) and read aligners (NextGenMap and GSNAP). The only exception was Bowtie2 whose read alignments yielded very weak bias in the SGA and SOAPdenovo2 assemblies and no bias in the ALLPATHS-LG assembly when calling variants with GATK. However, the alignment of the resequencing data with Bowtie2 against the simulated contig assemblies generated considerably fewer SNPs in all positions compared to BWA (Table 4) suggesting decreased sensitivity of the aligner.

Previous reports also showed the reduced sensitivity of Bowtie2 [35, 36] while others revealed only minor [38] or inconclusive [40] differences between Bowtie2 and BWA. Interestingly, Li attributes the differences between variants called from BWA and Bowtie2 alignments to lower mapping scores that Bowtie2 assigns to reads with additional suboptimal alignments [36]. Since similar repetitive sequences are likely to appear multiple times in the assembly, the respective primary alignments would have very low scores and they would rarely produce variants. On the other hand, such drastic reduction in the number of SNPs between BWA and Bowtie2 alignments is unlikely to stem exclusively from the false positives called by BWA. Therefore, further research is needed to determine whether Bowtie2 actually outperforms BWA in terms of both sensitivity and specificity when calling variants against draft genome assemblies.

Theoretically, it may be beneficial for resequencing projects to align reads previously used to construct the reference assembly, call variants and exclude them from the variants called in resequenced individuals. If the involved regions are completely conserved between the reference individual and a resequenced individual, those SNPs would be called for the resequenced individual as well and they would be false positives. However, this type of filtering produced very minor effects on the positional bias in the data sets we analysed, especially after the removal of SNPs located in repetitive elements.

The extent of positional bias obviously depends on the quality of the reference genome assembly. High quality assemblies have reduced number of contigs or scaffolds. Thus, they will have fewer locations to call the bias-causing SNPs. For many projects dealing with non-model organisms, it may not be realistic to construct genome assemblies with adequate quality in sufficiently short time. Moreover, even when a high quality assembly does not readily exhibit a strong positional bias, it may still appear after mapping scaffold coordinates to contig coordinates. We found the evidence for such bias masking in our analyses. Increased number of repetitive elements in a genome would also yield higher positional bias while lowering assembly quality. Therefore, regardless of the assembly quality it would be important to filter SNPs that appear in repetitive elements in order to avoid potential complications.

A more conservative approach would involve filtering out all variants near contig ends. When using a sufficiently large threshold (greater than read length), this would remove the positional bias completely. However, any true variants in those regions will also be lost. To apply such a filter, it would be necessary to know contig coordinates within scaffolds and this information may not be readily available. In any case, this kind of filtering should be done in addition to the filtering of variants in repetitive elements because some repetitive sequences would not be located near contig ends.

## Conclusions

This study describes positional bias in variant calls that are made against draft genomes constructed with several popular assemblers. The variant calls that cause the bias are mostly false positives that arise from aligning the reads originated in spatially remote repeated sequences or homologous regions. The degree of the bias depends on the choice of tools, configuration, and underlying data set. However, the bias is likely to affect many projects that rely on de novo draft assemblies generated from short read data. The bias can be mitigated by removing variants located in repetitive elements that can be identified by programs such as RepeatMasker [32]. More conservatively, the bias can also be removed by filtering out all variants located near contig ends. Our findings will help researchers who work on resequencing projects to recognise and reduce the position bias, which will result in higher quality variant data sets.

## Additional file

Additional file 1 Supplementary figures. (PDF 1499 kb)

## Abbreviations

DDBJ: DNA Data Bank of Japan
DP: depth of coverage
HTS: high-throughput sequencing
indel: insertion or deletion
LTR: long terminal repeat
MQ: mapping quality
NCBI: National Center for Biotechnology Information
QUAL: variant quality
SNP: single nucleotide polymorphism
TAIR: The Arabidopsis Information Resource
VCF: variant call format
