# Supplementary material for: Positional bias in variant calls against draft reference assemblies
Source: BMC Genomics. 2017 Mar 28;18:263. doi: 10.1186/s12864-017-3637-2 (PMC5368935; doi:10.1186/s12864-017-3637-2)
Supplement: Additional file 1 — Supplementary figures. (PDF 1499 kb) [file 12864_2017_3637_MOESM1_ESM.pdf]

Positional bias in variant calls against draft reference assemblies

Roman V. Briskine[1] and Kentaro K. Shimizu[1,2]

[1]Department of Evolutionary Biology and Environmental Studies, University of Zurich, Winterthurerstrasse 190, Zurich, CH-8057, Switzerland
[2]Kihara Institute for Biological Research, Yokohama City University, 641-12 Maioka, Totsuka-ward, Yokohama, 244-0813, Japan

Supplementary Figures

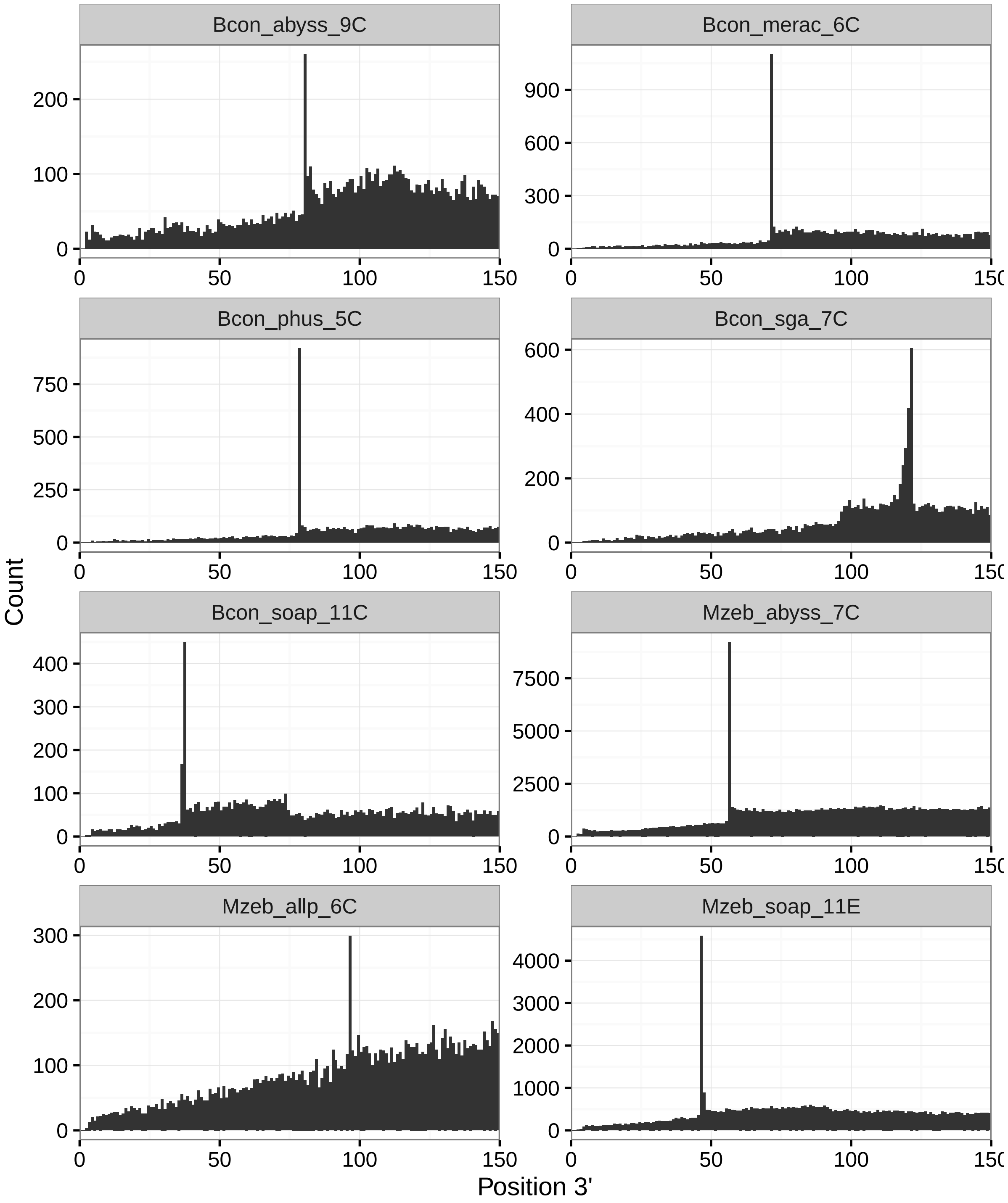

Figure S1: Distribution of SNP positions at the 3´ end of **contigs** in *B. constrictor* and *M. zebra* assemblies.

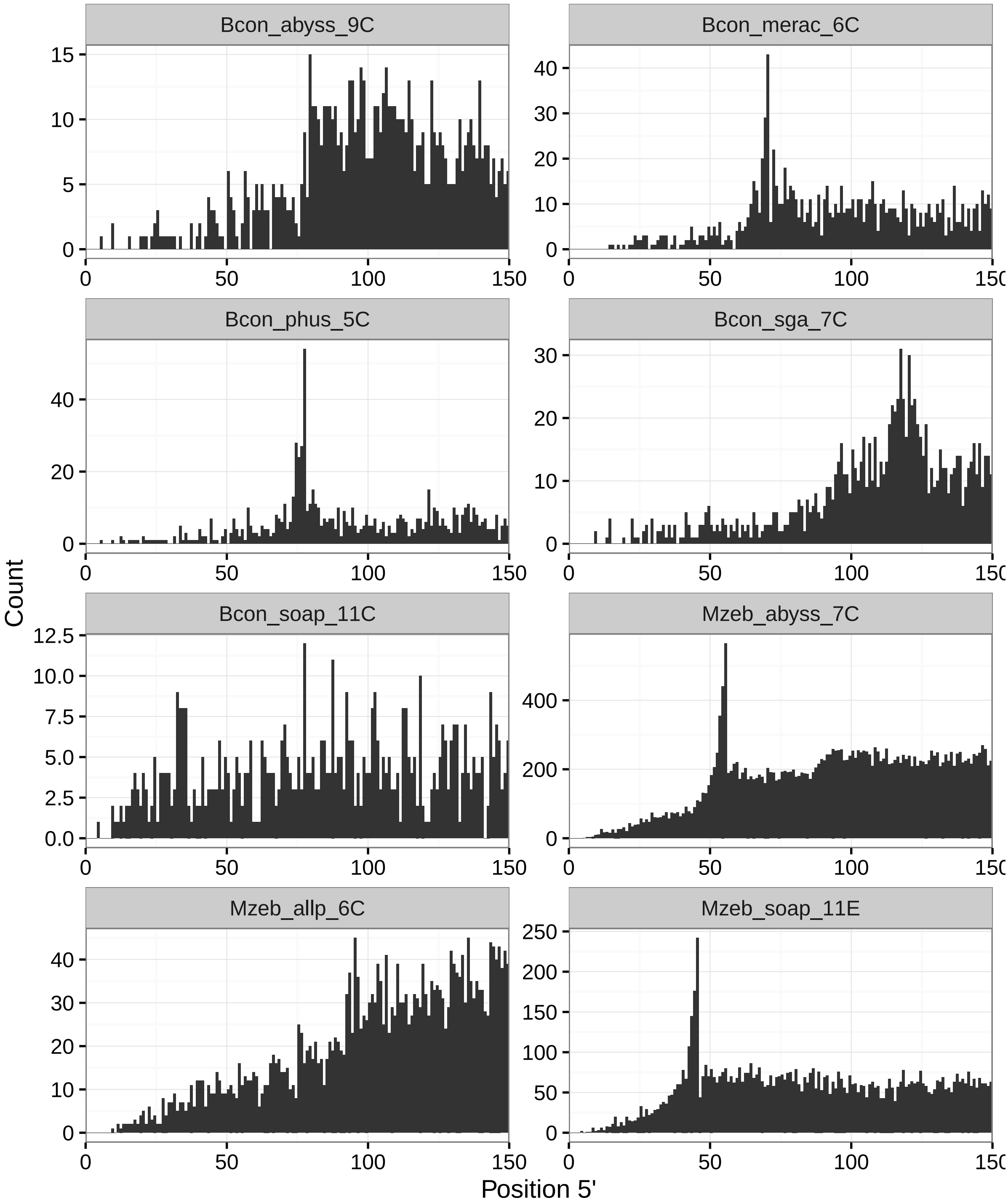

Figure S2: Distribution of indel positions at the 5´ end of **contigs** in *B. constrictor* and *M. zebra* assemblies.

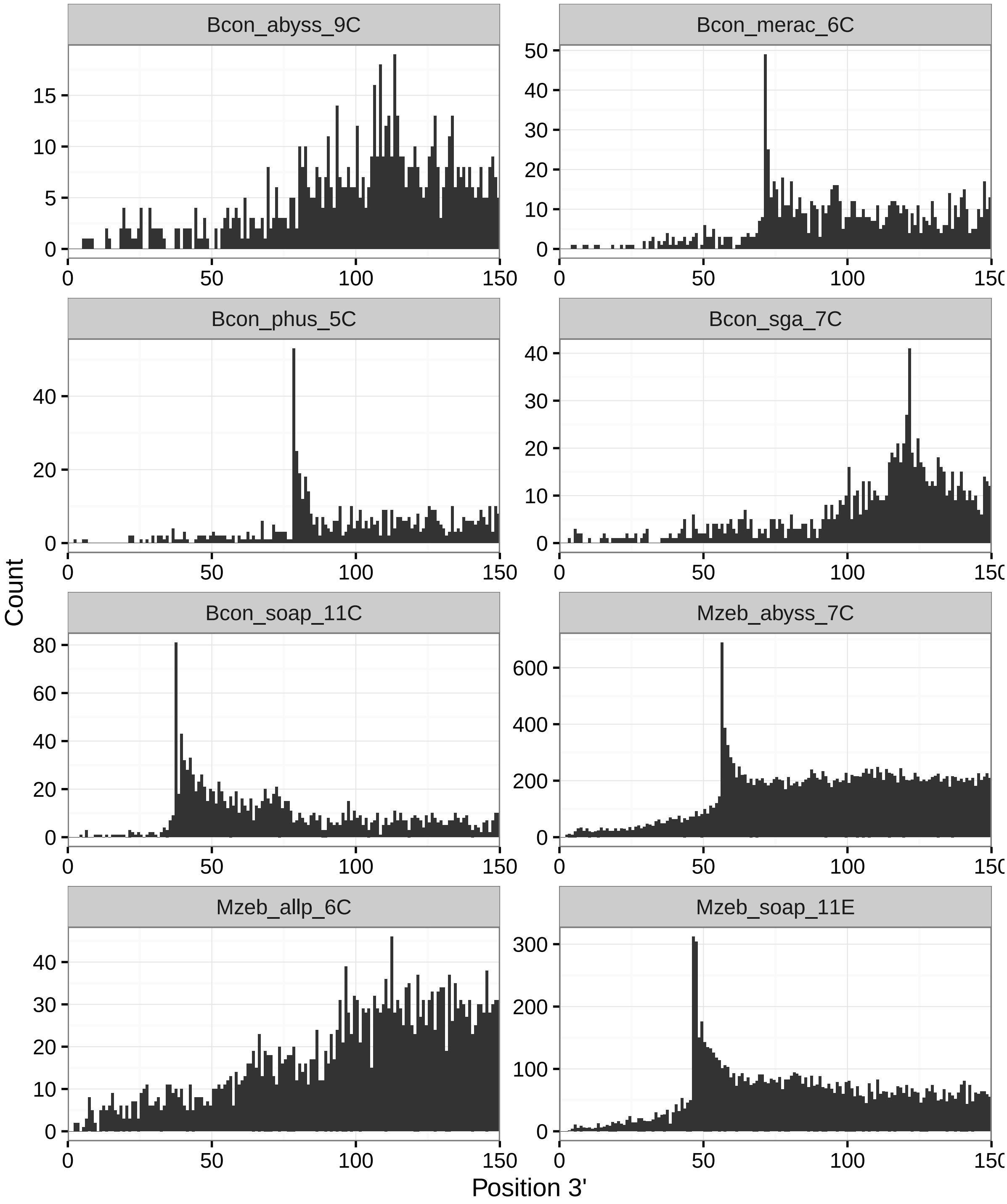

Figure S3: Distribution of indel positions at the 3´ end of **contigs** in *B. constrictor* and *M. zebra* assemblies.

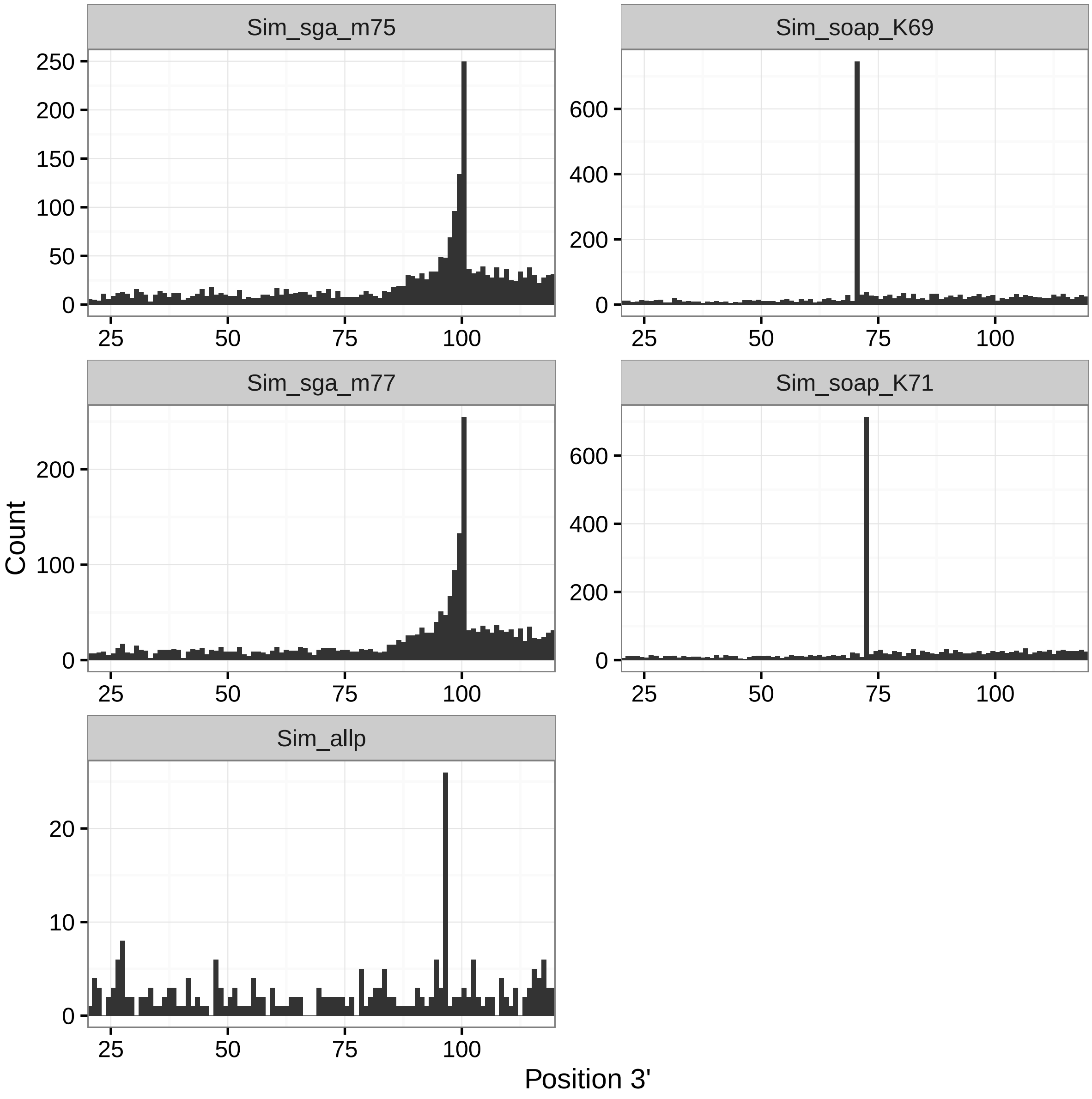

Figure S4: Distribution of SNP positions at the 3´ end of **contigs** in the simulated data set.

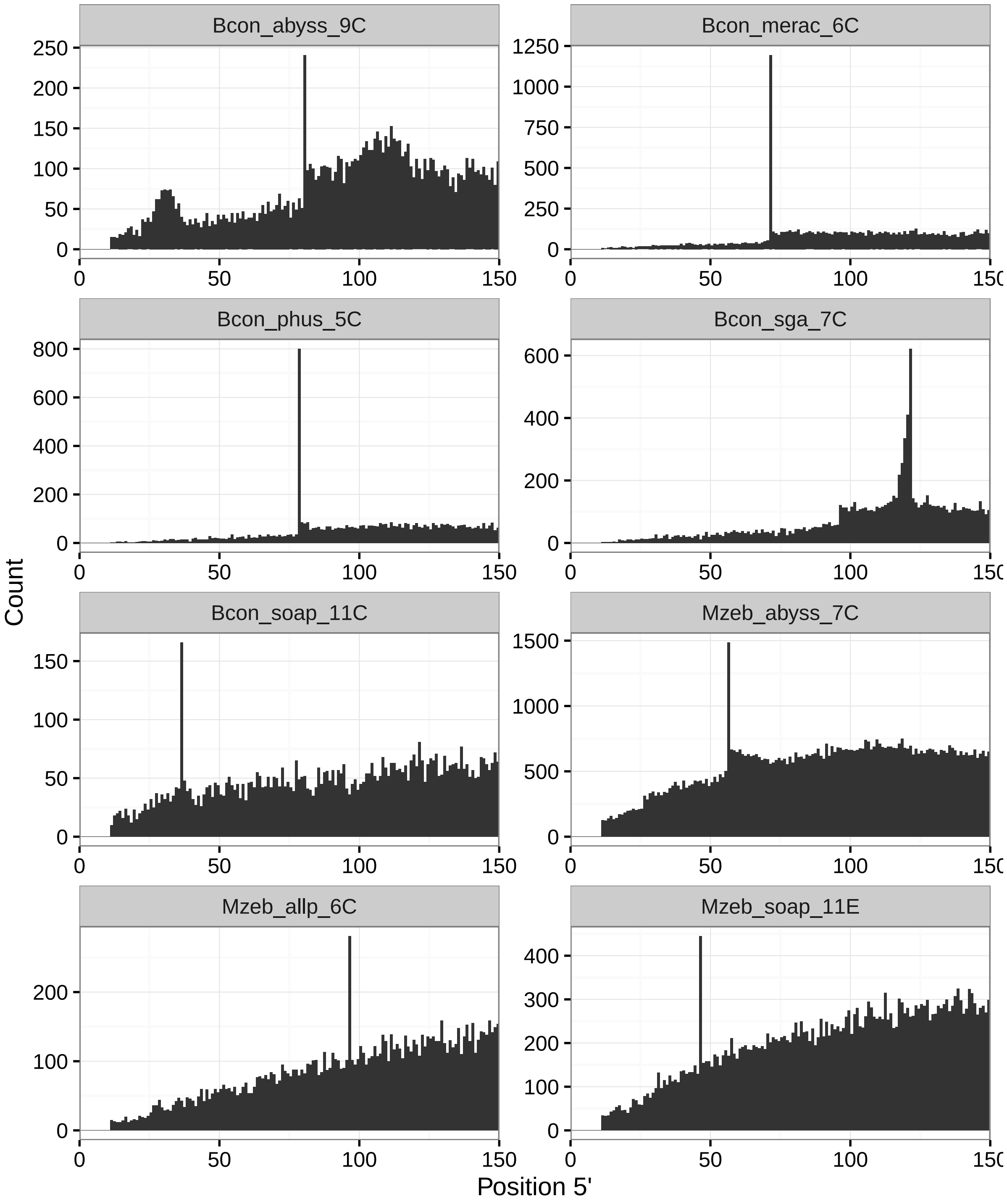

Figure S5: Distribution of SNP positions at the 5´ end of **long contigs** (>= 500 bp) in *B. constrictor* and *M. zebra* assemblies.

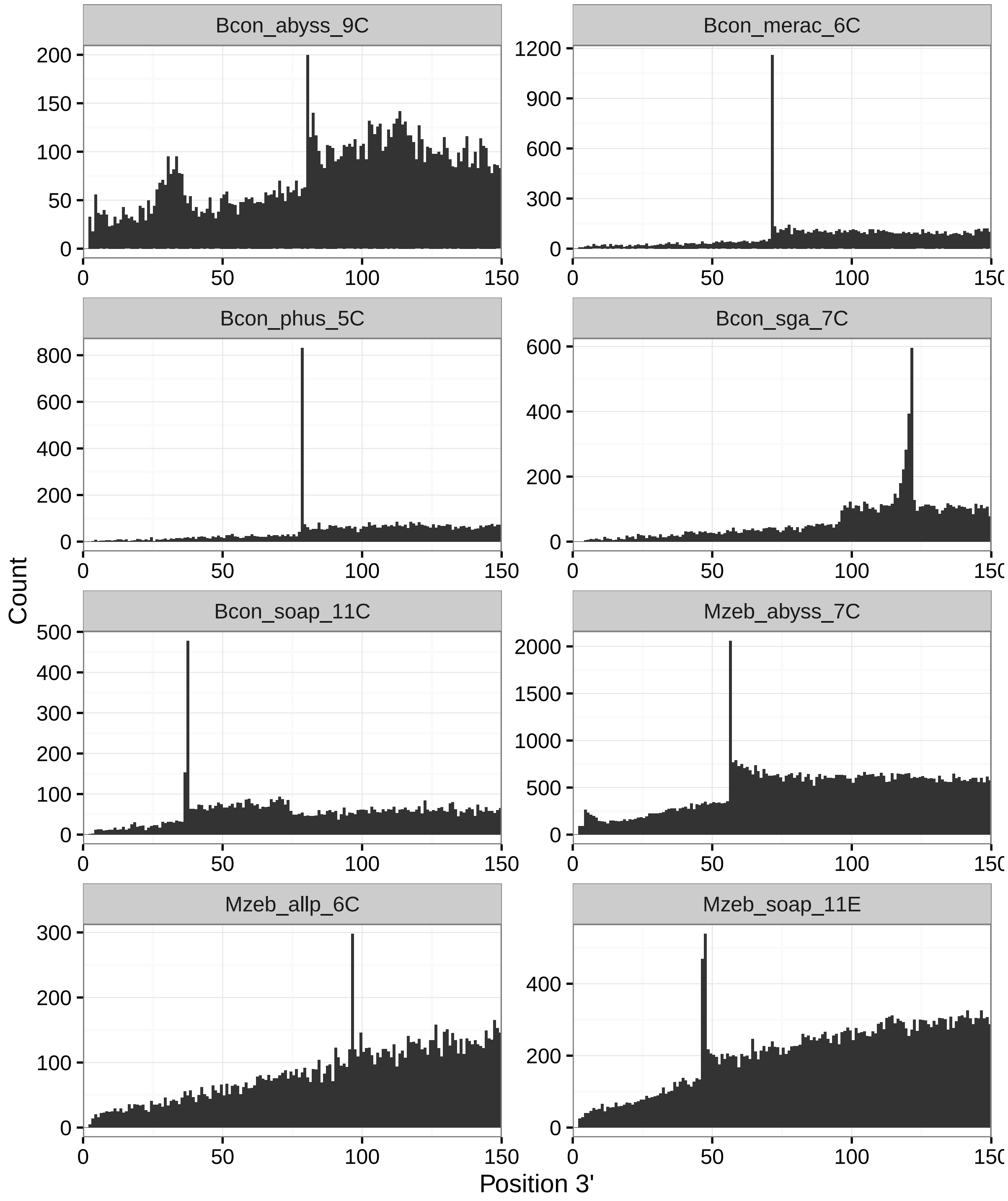

Figure S6: Distribution of SNP positions at the 3´ end of **long contigs** (>= 500 bp) in *B. constrictor* and *M. zebra* assemblies.

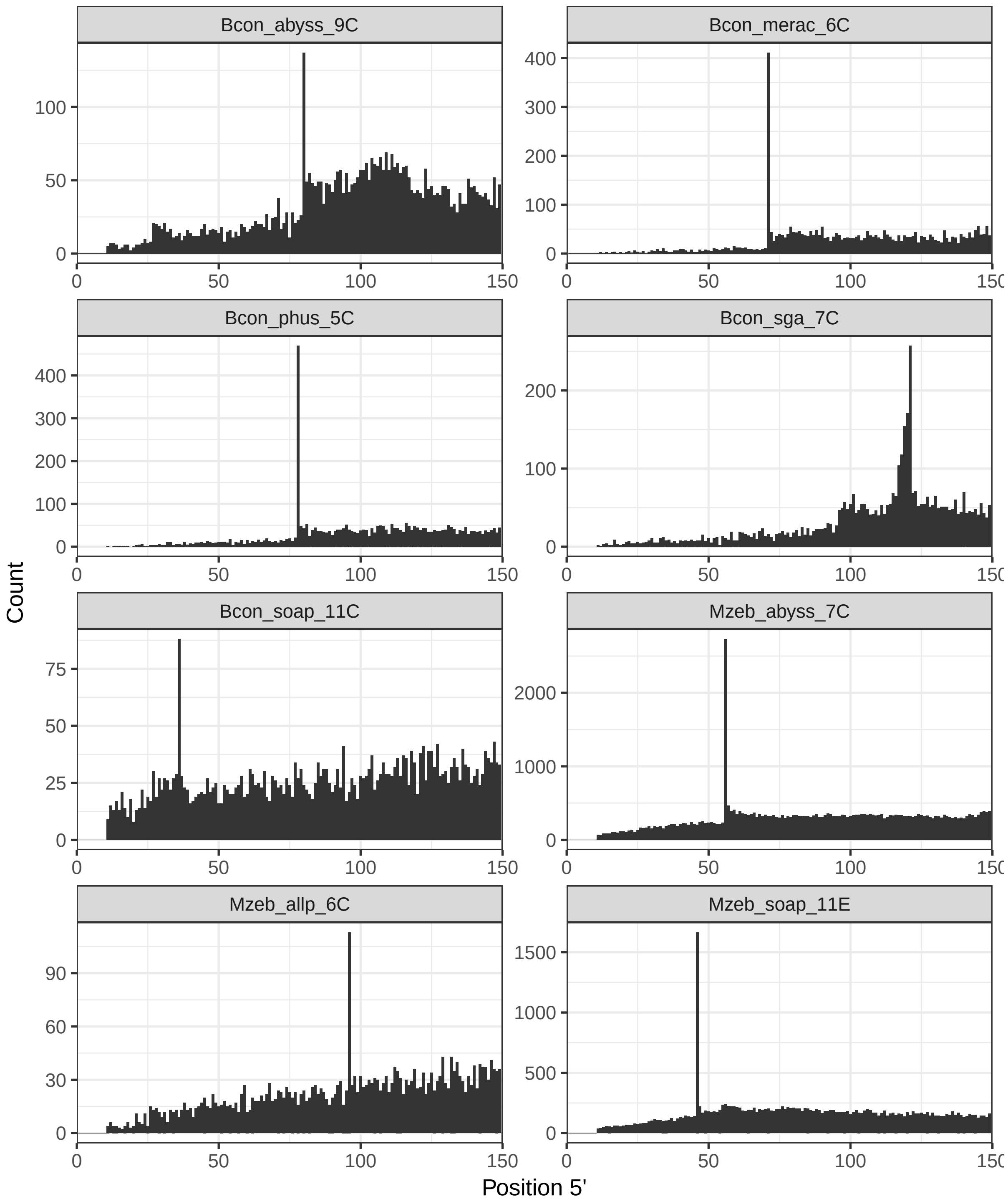

Figure S7: Distribution of SNP positions at the 5´ end of **contigs** in regions where the coverage does not exceed expected levels in *B. constrictor* and *M. zebra* assemblies.

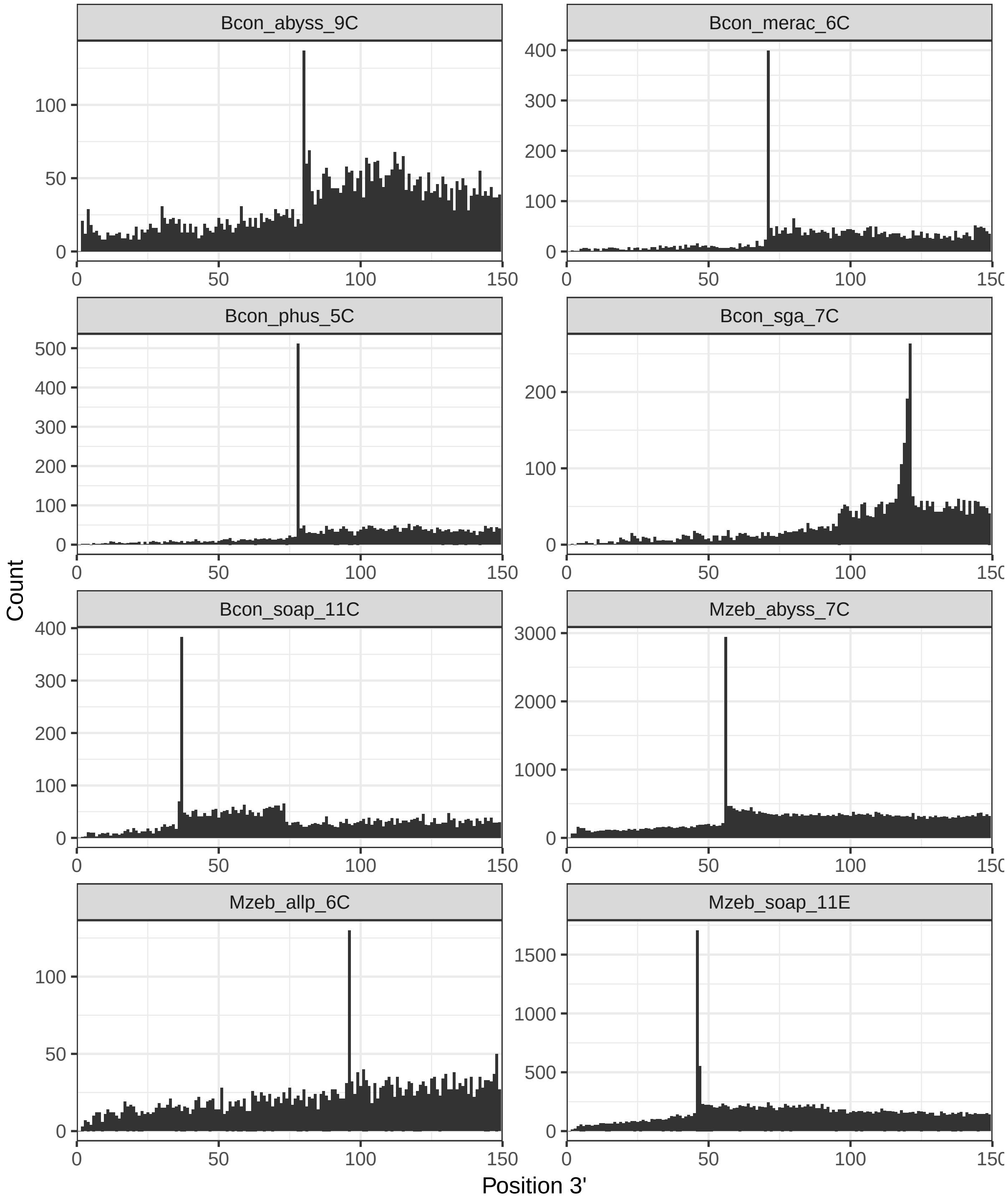

Figure S8: Distribution of SNP positions at the 3´ end of **contigs** in regions where the coverage does not exceed expected levels in *b. constrictor* and *M. zebra* assemblies.

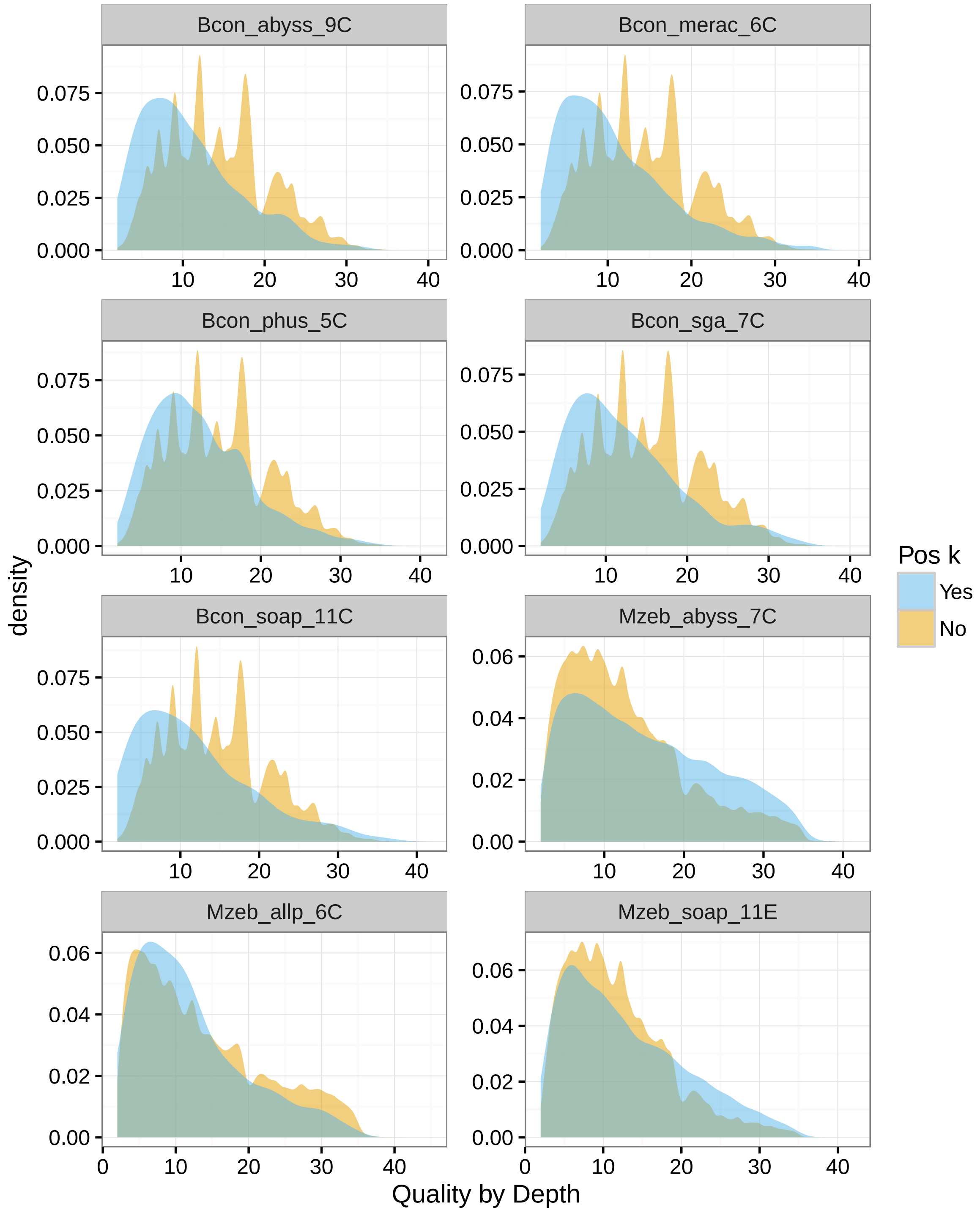

Figure S9: Distribution of QD values (quality by depth) for SNPs at position *k* and SNPs at other positions in *B. constrictor* and *M. zebra* assemblies. SNPs with QD below 2.0 were removed as per GATK best practices.

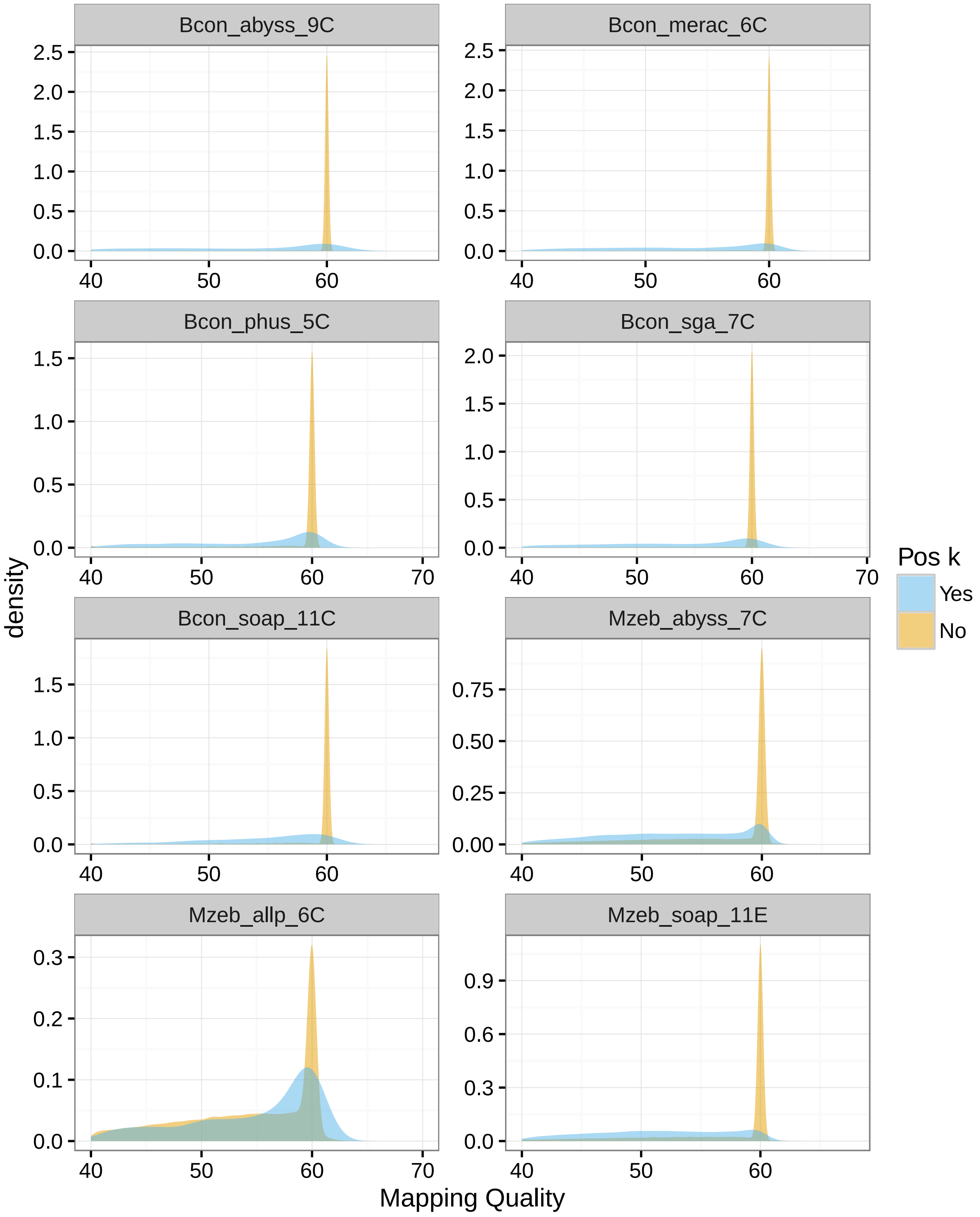

Figure S10: Distribution of MQ values (root mean square of the mapping quality) for SNPs at position *k* and SNPs at other positions in *B. constrictor* and *M. zebra* assemblies. SNPs with MQ below 40 were removed as per GATK best practices.

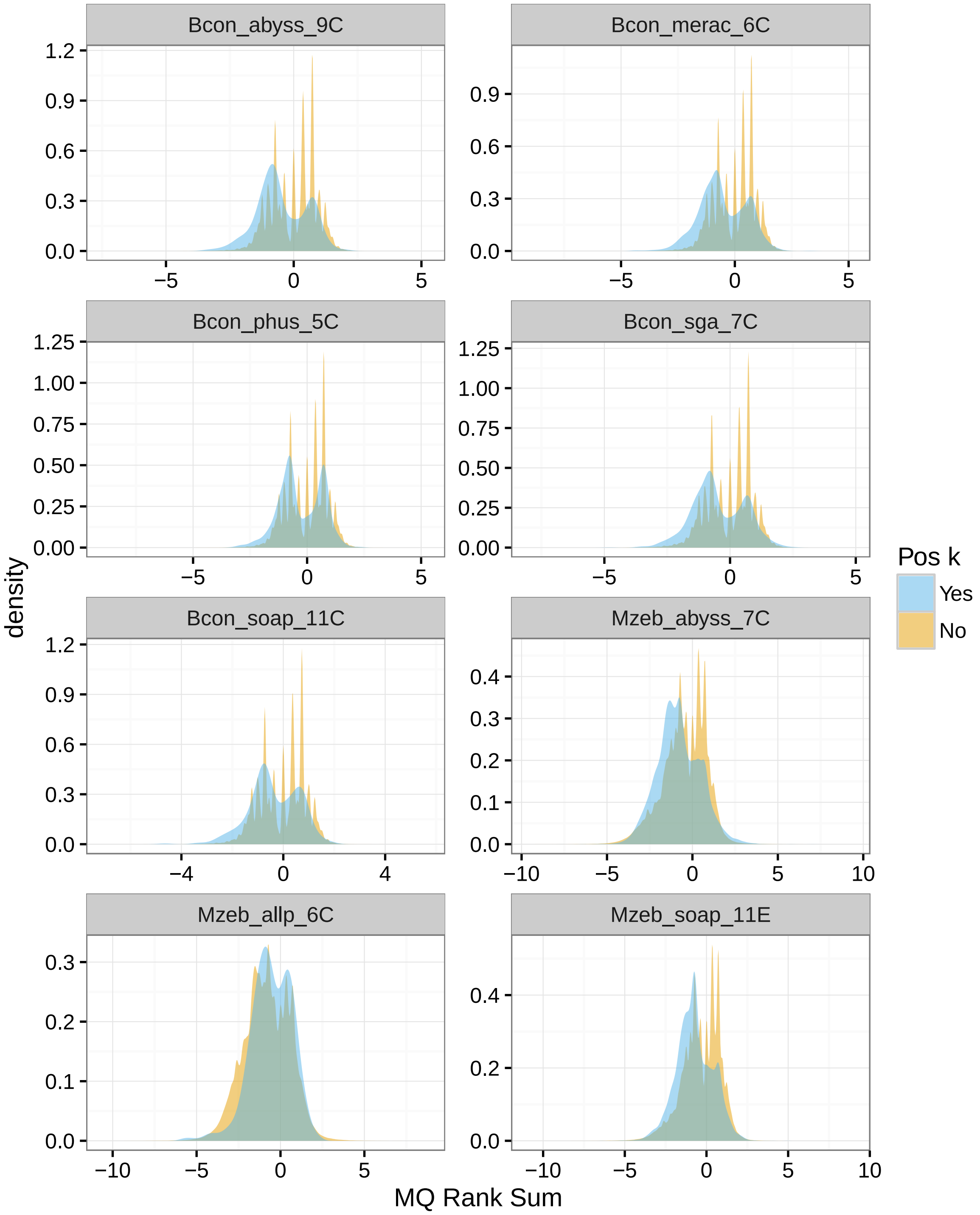

Figure S11: Distribution of MQRankSum values (mapping quality ranksum test) for SNPs at position *k* and SNPs at other positions in *B. constrictor* and *M. zebra* assemblies. SNPs with MQRankSum below -12.5 were removed as per GATK best practices.

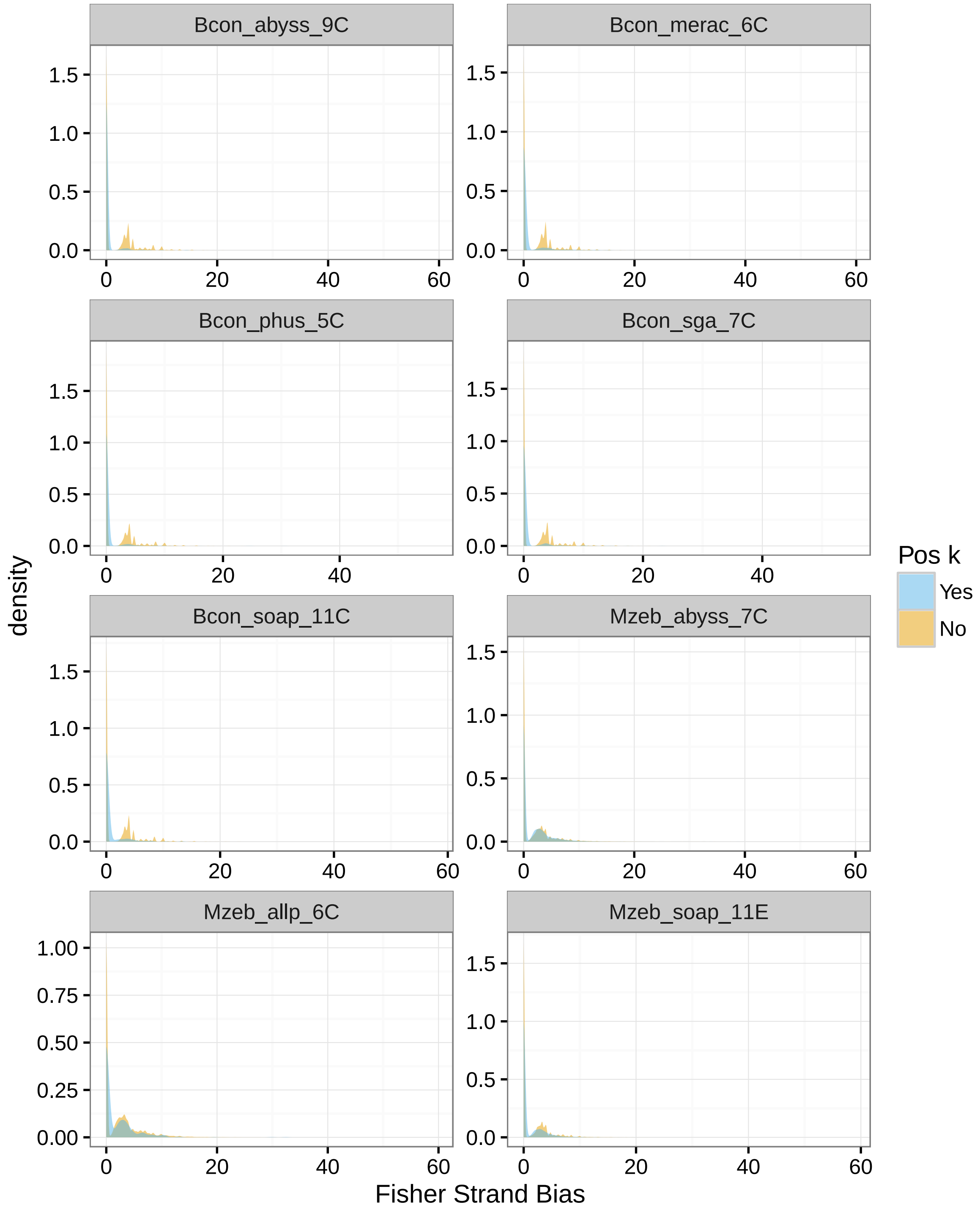

Figure S12: Distribution of FS values (Fisher's exact test to measure strand bias) for SNPs at position *k* and SNPs at other positions in *B. constrictor* and *M. zebra* assemblies. SNPs with FS over 60 were removed as per GATK best practices.

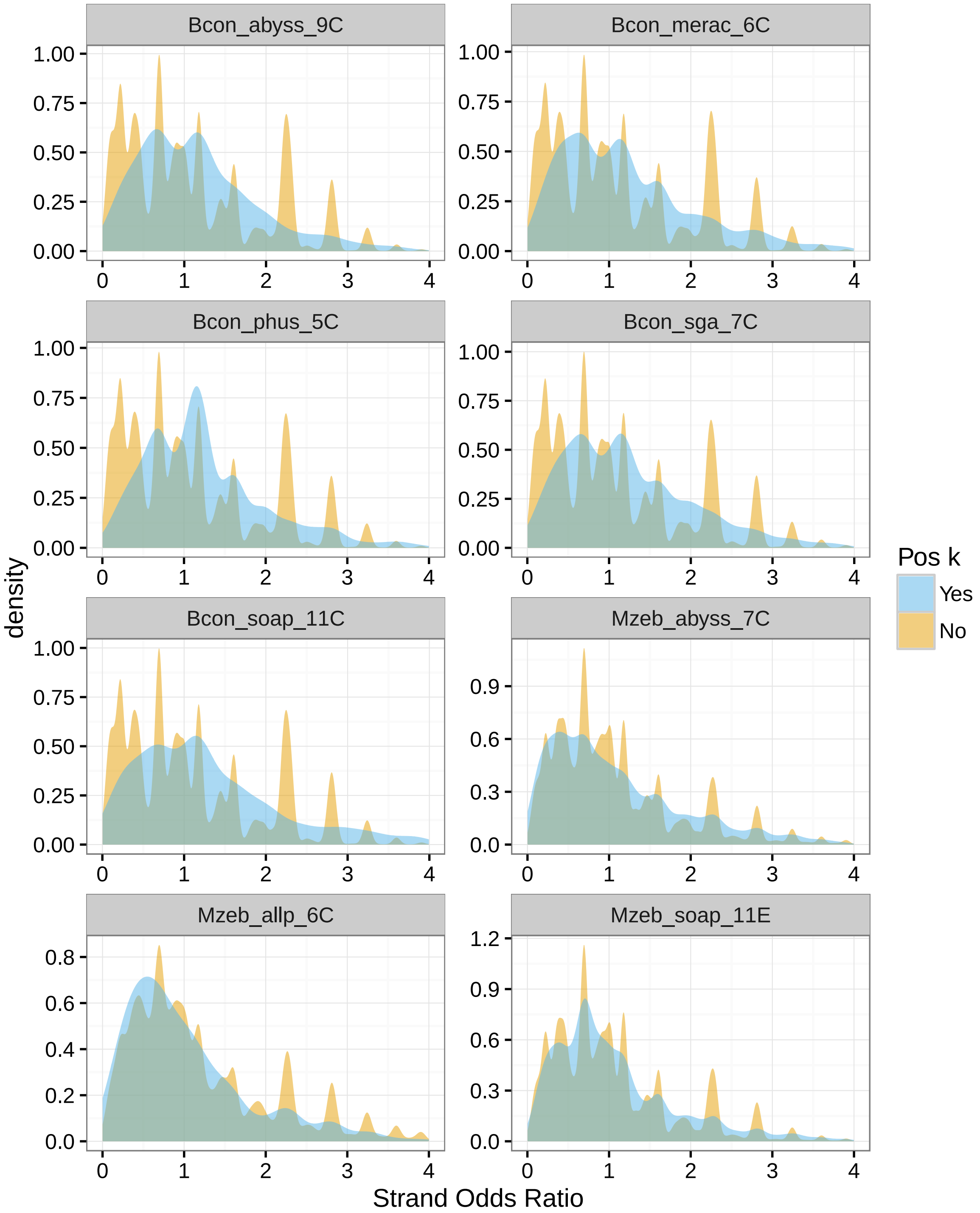

Figure S13: Distribution of SOR values (strand bias odds ratio) for SNPs at position *k* and SNPs at other positions in *B. constrictor* and *M. zebra* assemblies. SNPs with SOR over 4.0 were removed as per GATK best practices.

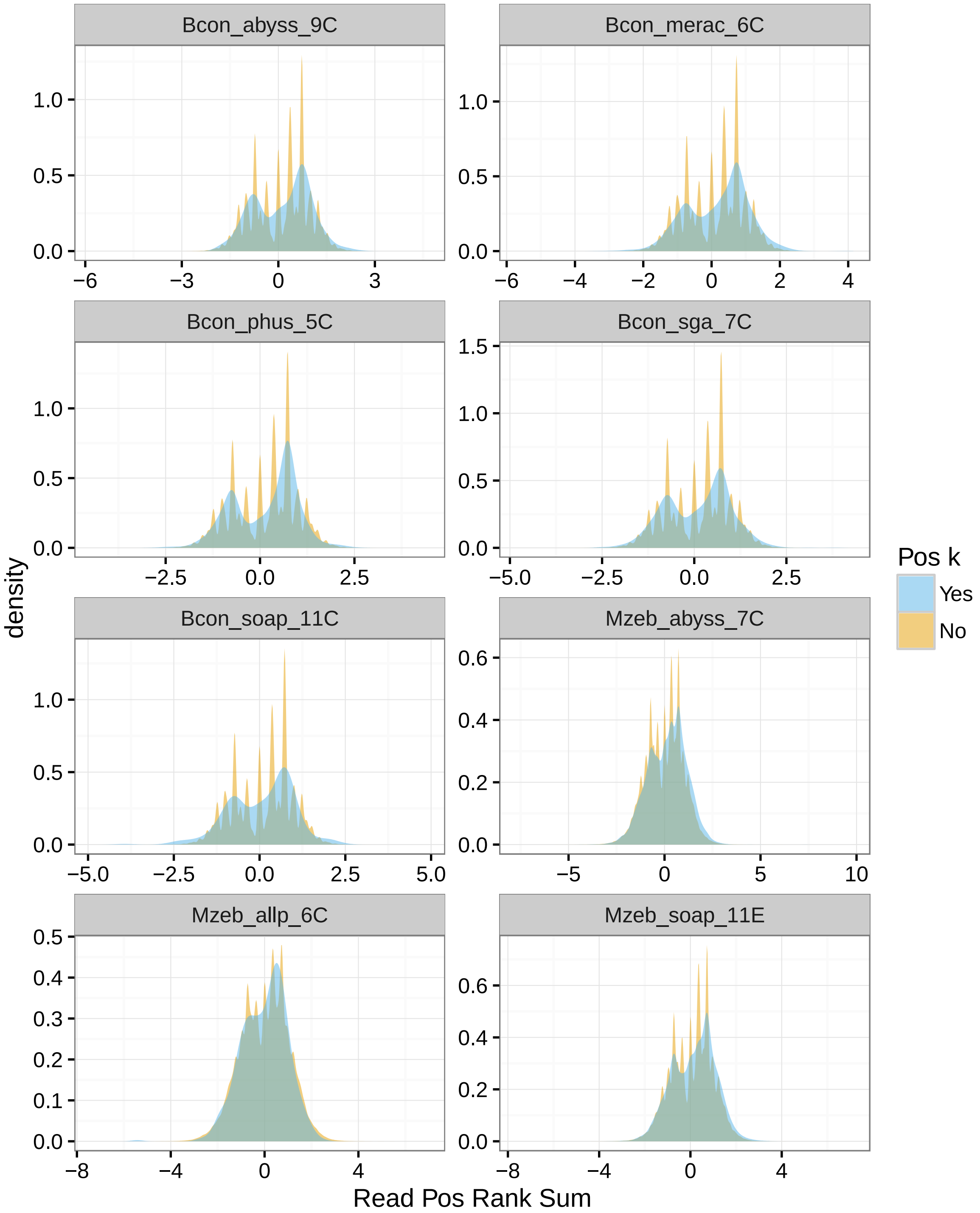

Figure S14: Distribution of ReadPosRankSum values (read position rank sum test) for SNPs at position *k* and SNPs at other positions in *B. constrictor* and *M. zebra* assemblies. SNPs with ReadPosRankSum below -8.0 were removed as per GATK best practices.

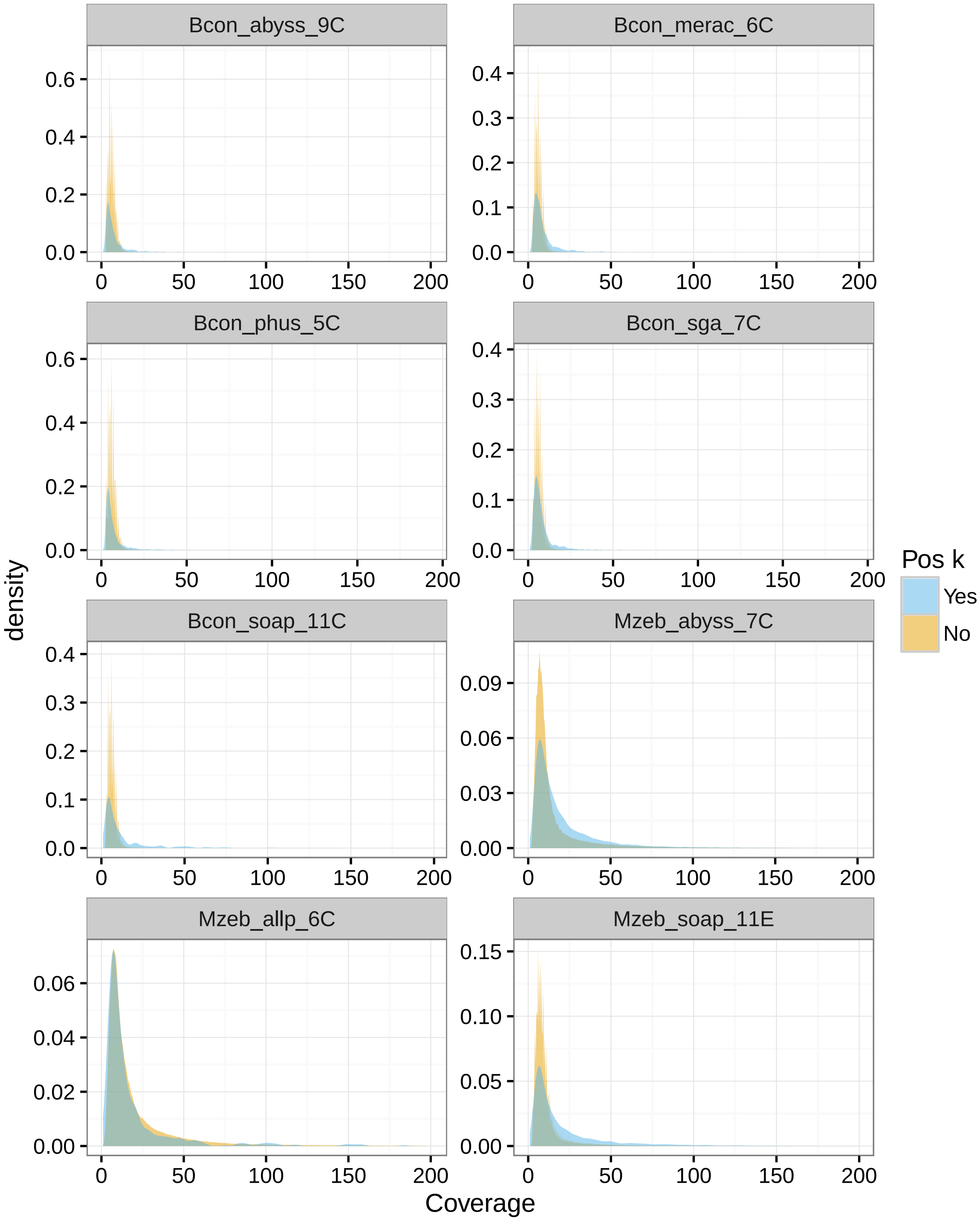

Figure S15: Distribution of DP values (depth of coverage) for SNPs at position *k* and SNPs at other positions in *B. constrictor* and *M. zebra* assemblies. SNPs with DP over 200 were removed as per GATK best practices.

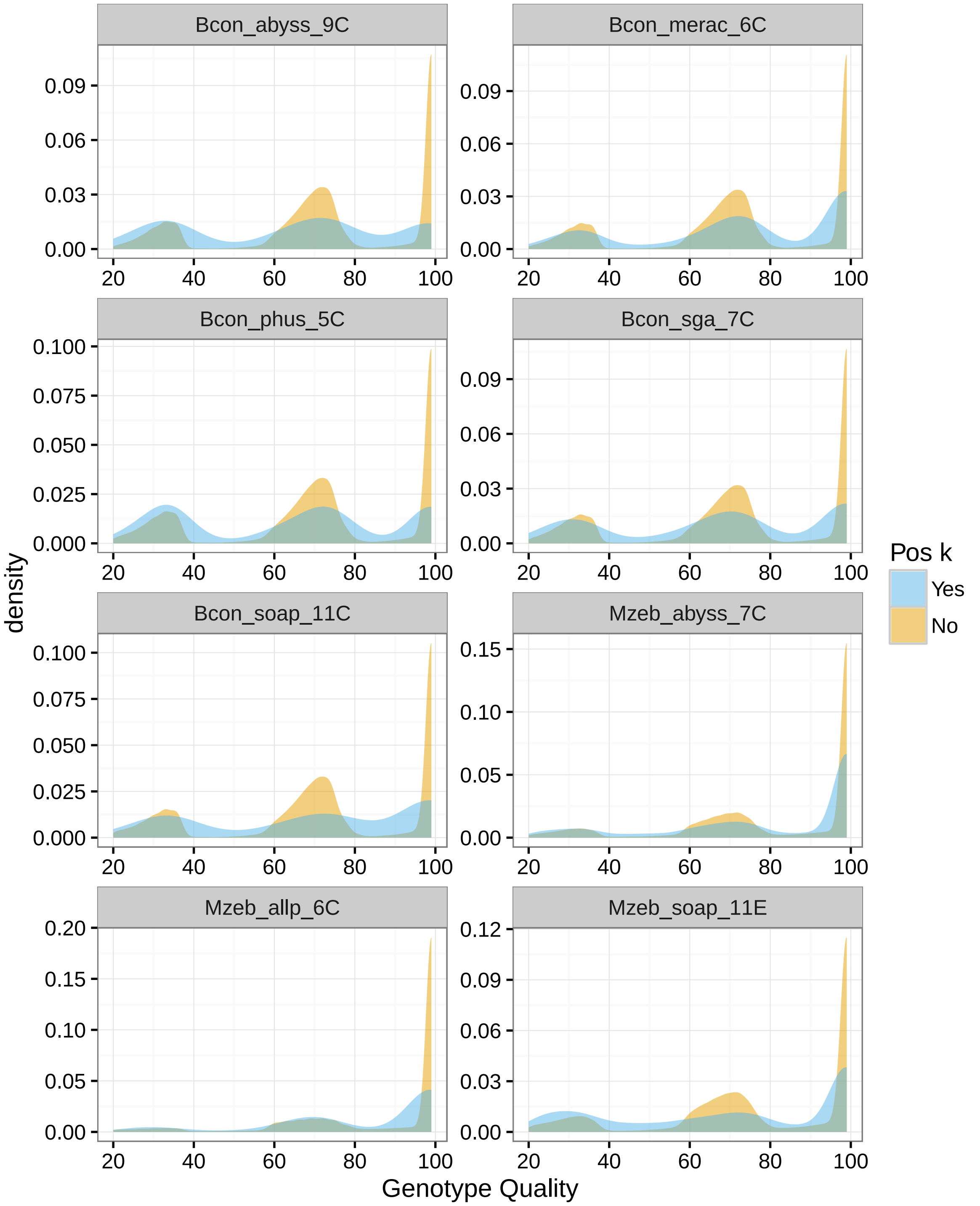

Figure S16: Distribution of GQ values (genotype quality) for SNPs at position *k* and SNPs at other positions in *B. constrictor* and *M. zebra* assemblies. SNPs with GQ below 20 were removed as per GATK best practices.

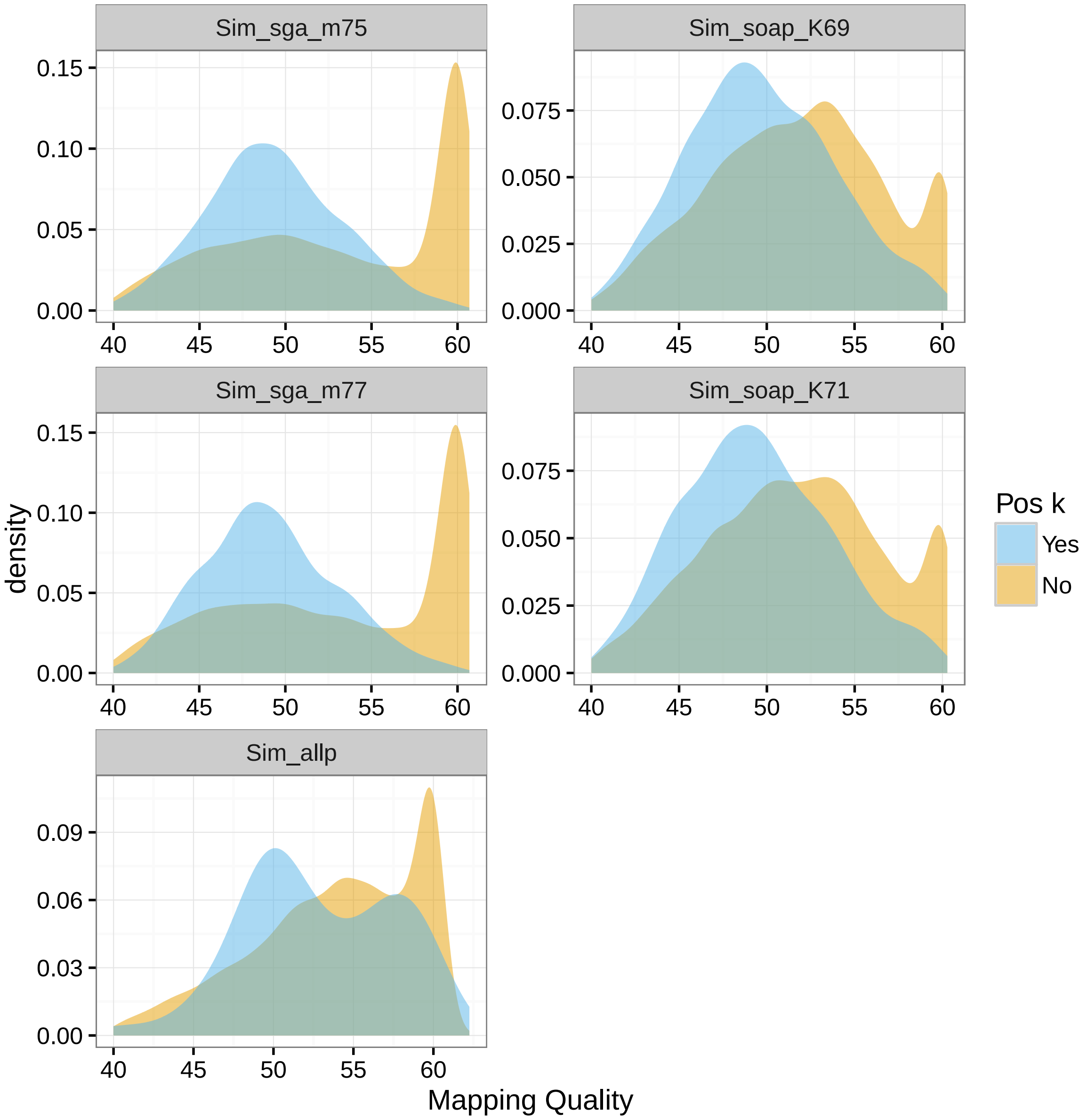

Figure S17: Distribution of MQ values (root mean square of the mapping quality) for SNPs at position *k* and SNPs at other positions in the simulated data set. SNPs with MQ below 40 were removed as per GATK best practices.

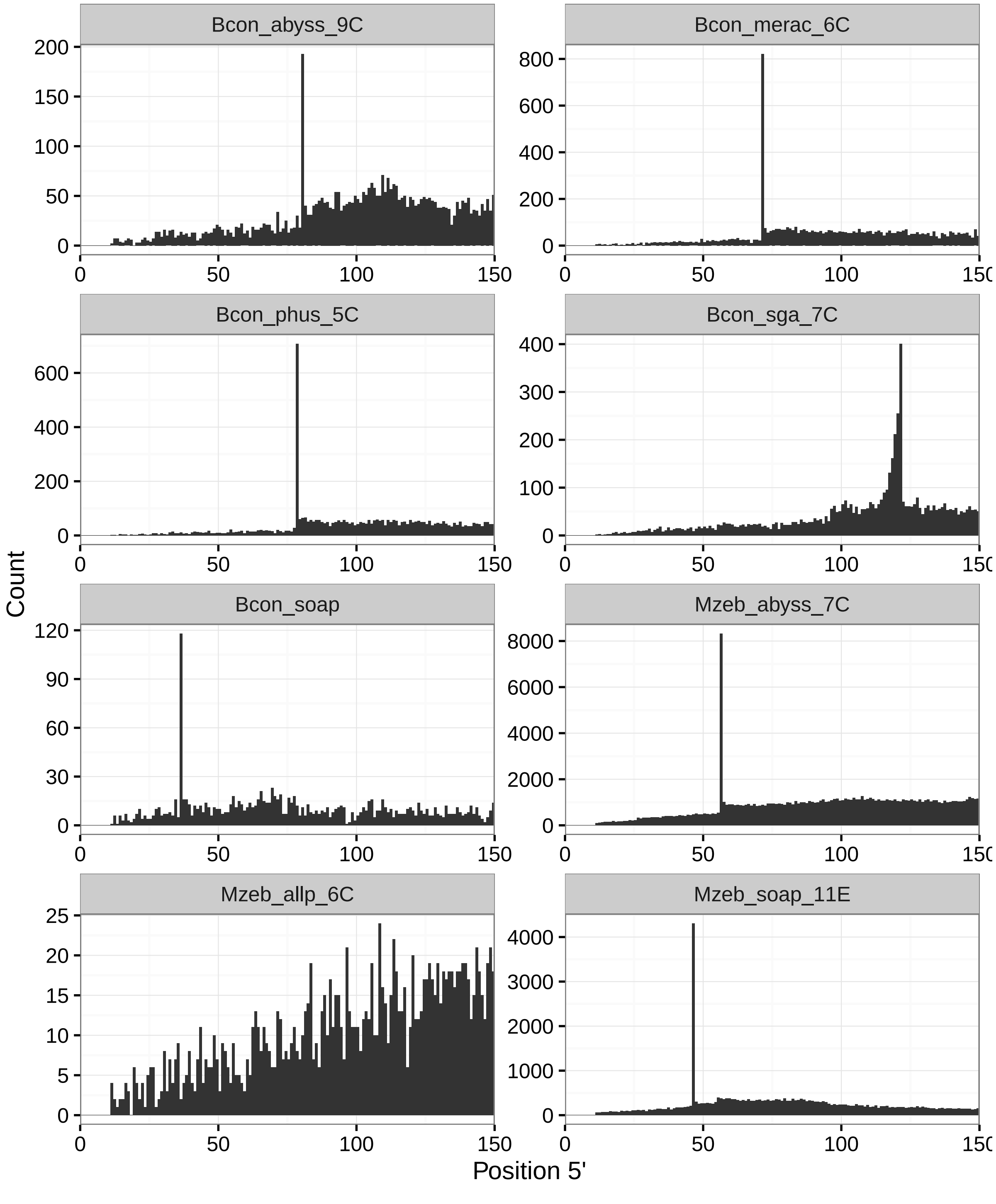

Figure S18: Distribution of SNP positions at the 5´ end of **scaffolds** in *B. constrictor* and *M. zebra* assemblies.

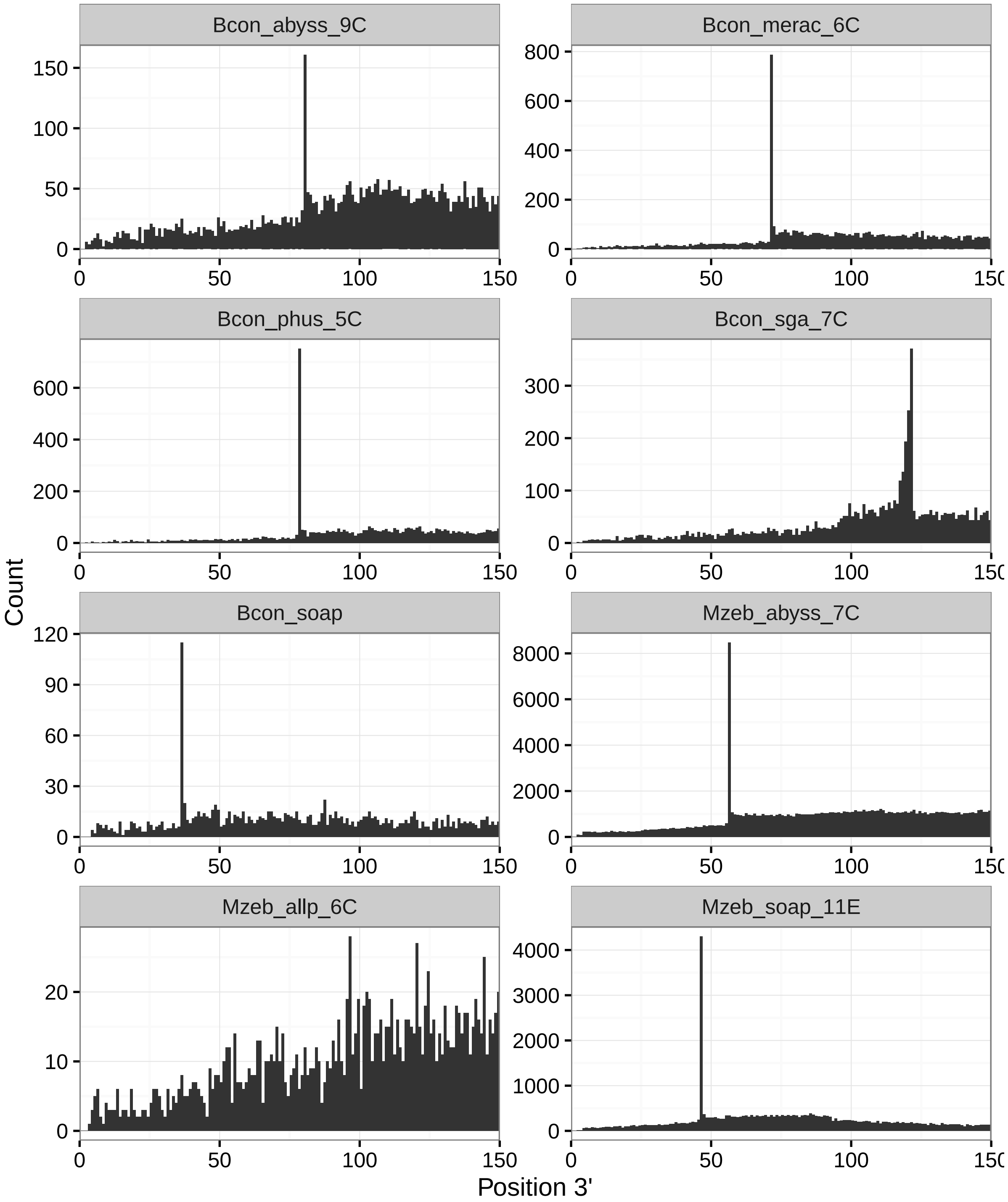

Figure S19: Distribution of SNP positions at the 3´ end of **scaffolds** in *B. constrictor* and *M. zebra* assemblies.

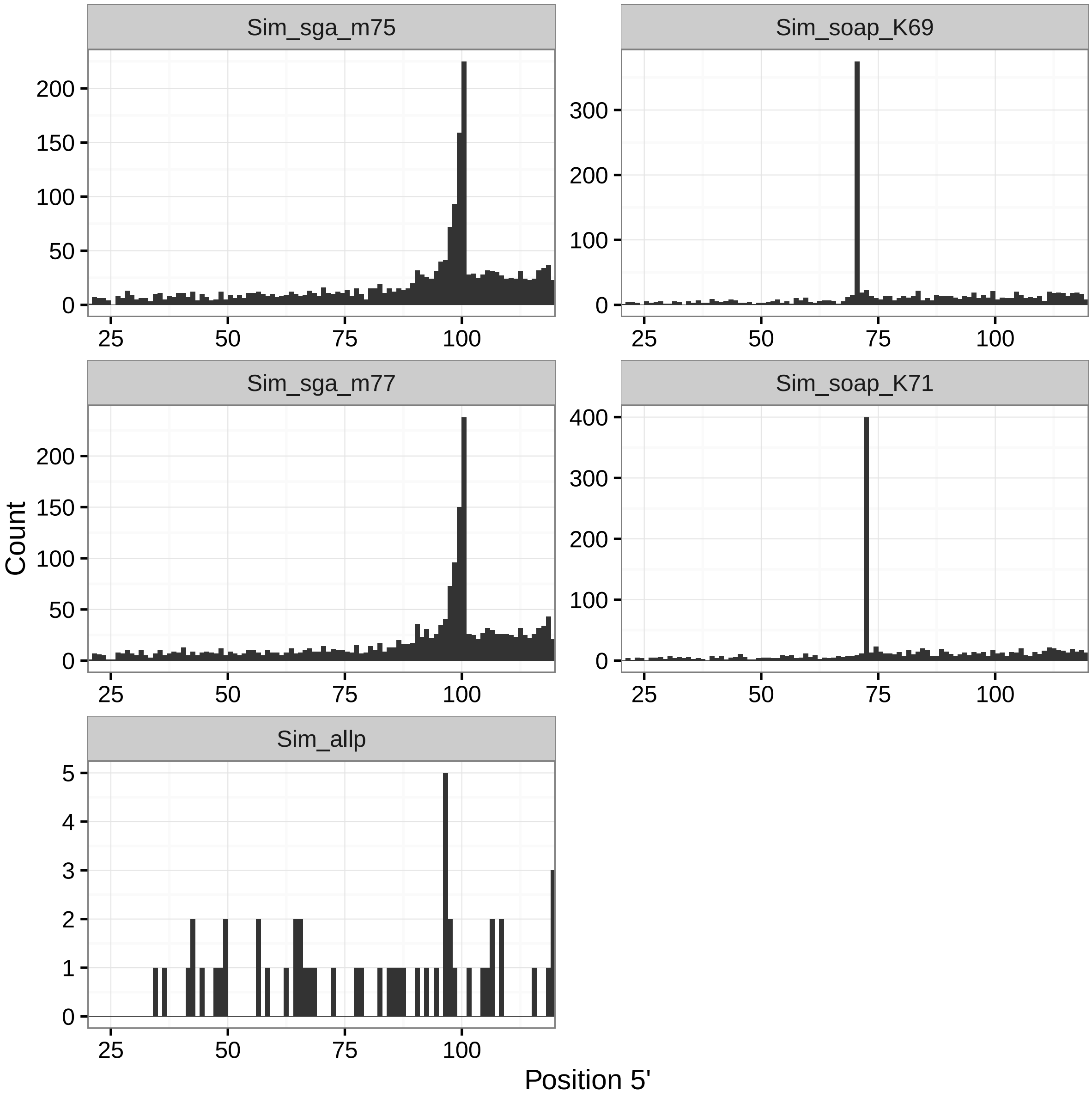

Figure S20: Distribution of SNP positions at the 5´ end of **scaffolds** in the simulated data set.

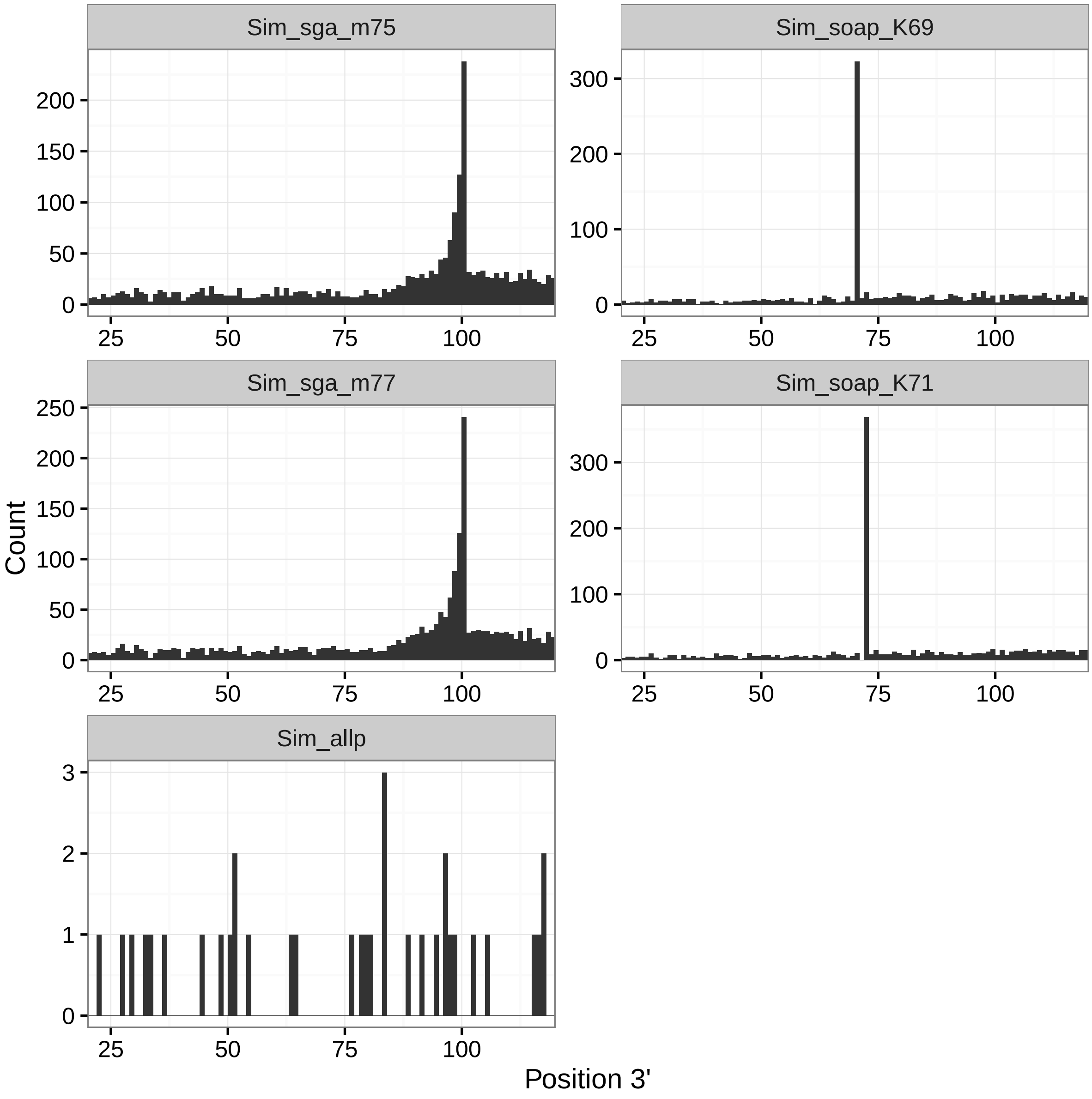

Figure S21: Distribution of SNP positions at the 3´ end of **scaffolds** in the simulated data set.

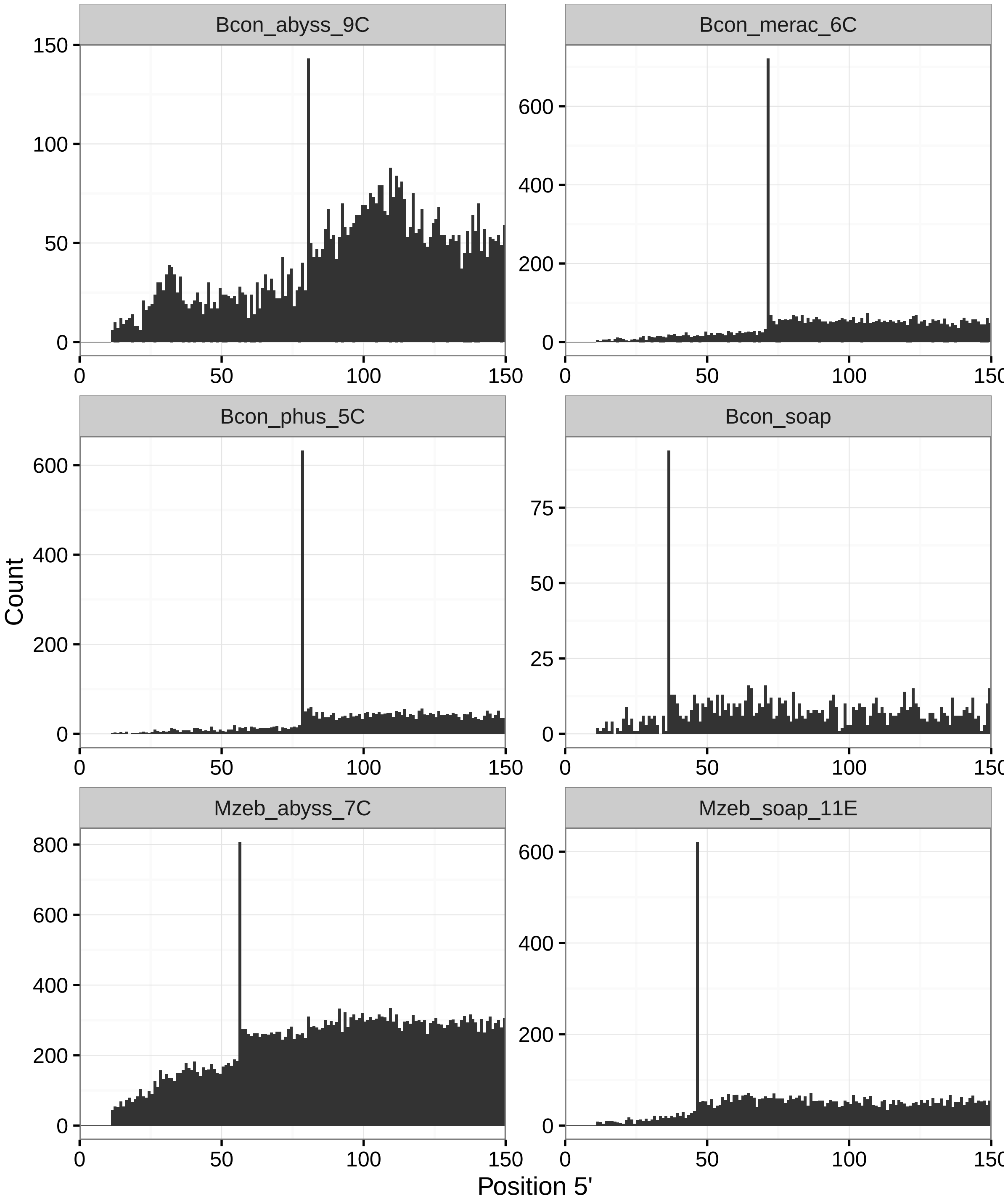

Figure S22: Distribution of SNP positions at the 5´ end of **long scaffolds** (>500 bp) in *B. constrictor* and *M. zebra* assemblies. The Mzeb_allp_6C and Bcon_sga_7C assemblies were not included because they had already contained only scaffolds 500 bp or longer.

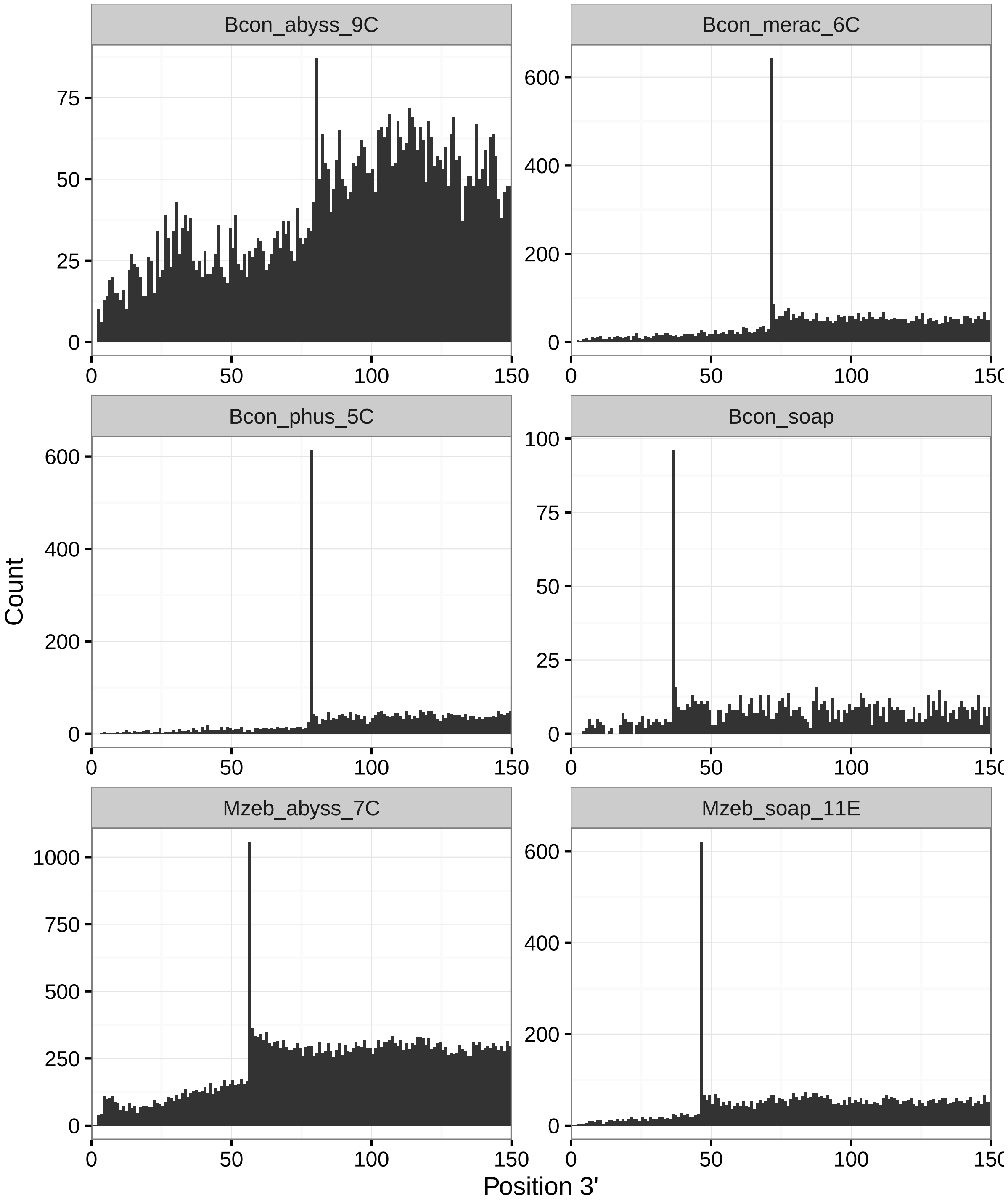

Figure S23: Distribution of SNP positions at the 3´ end of **long scaffolds** (>500 bp) in *B. constrictor* and *M. zebra* assemblies. The Mzeb_allp_6C and Bcon_sga_7C assemblies were not included because they had already contained only scaffolds 500 bp or longer.

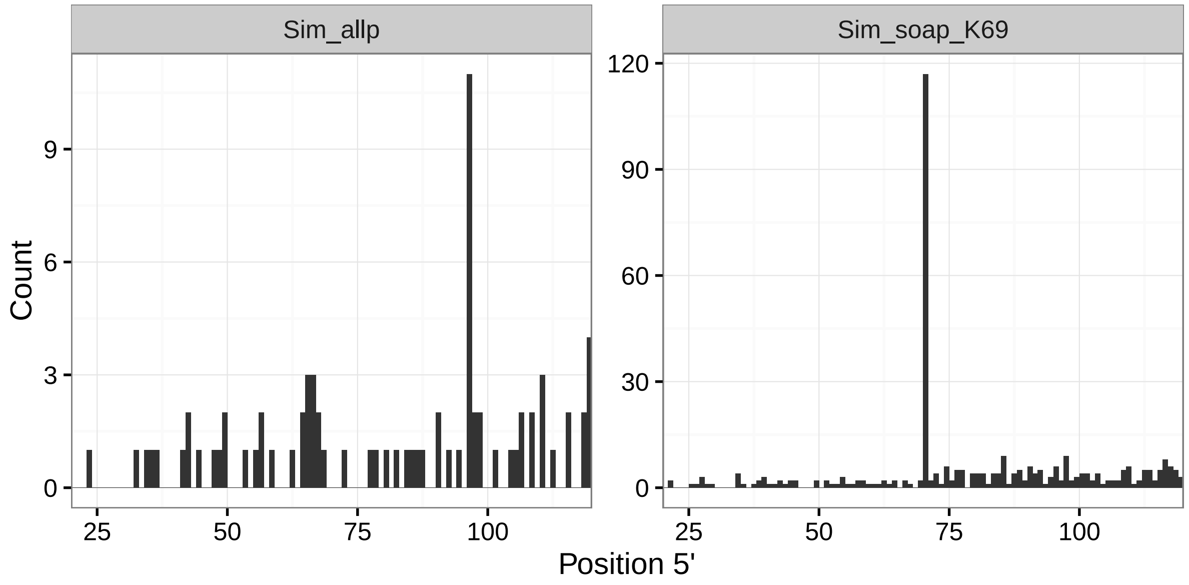

Figure S24: Distribution of SNP positions **transformed** from scaffold to contig coordinates at the 5´ end of contigs.

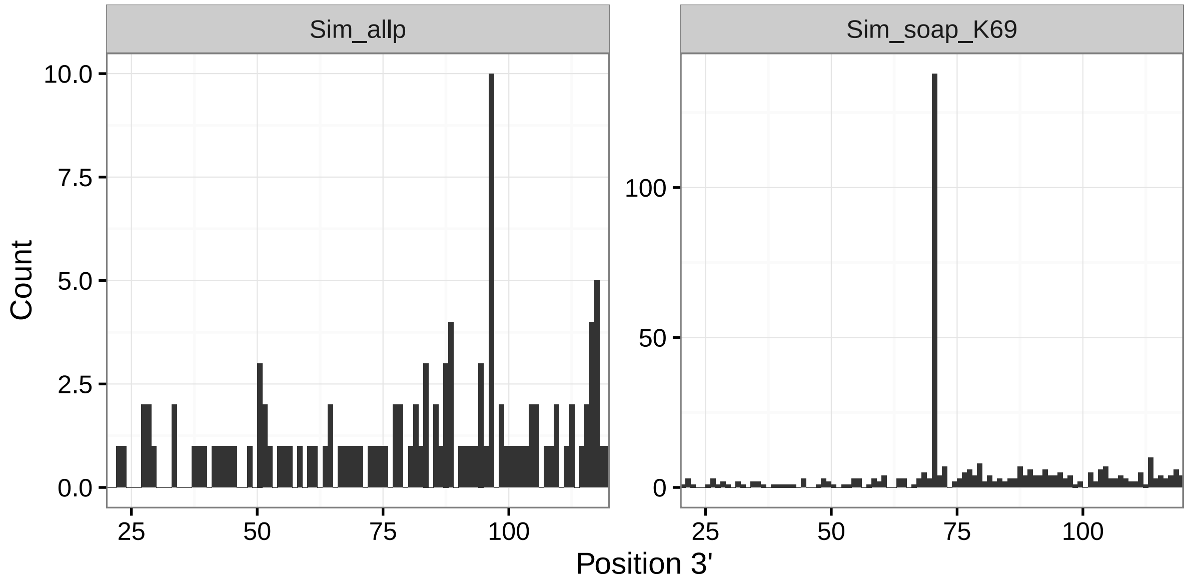

Figure S25: Distribution of SNP positions **transformed** from scaffold to contig coordinates at the 3´ end of contigs.

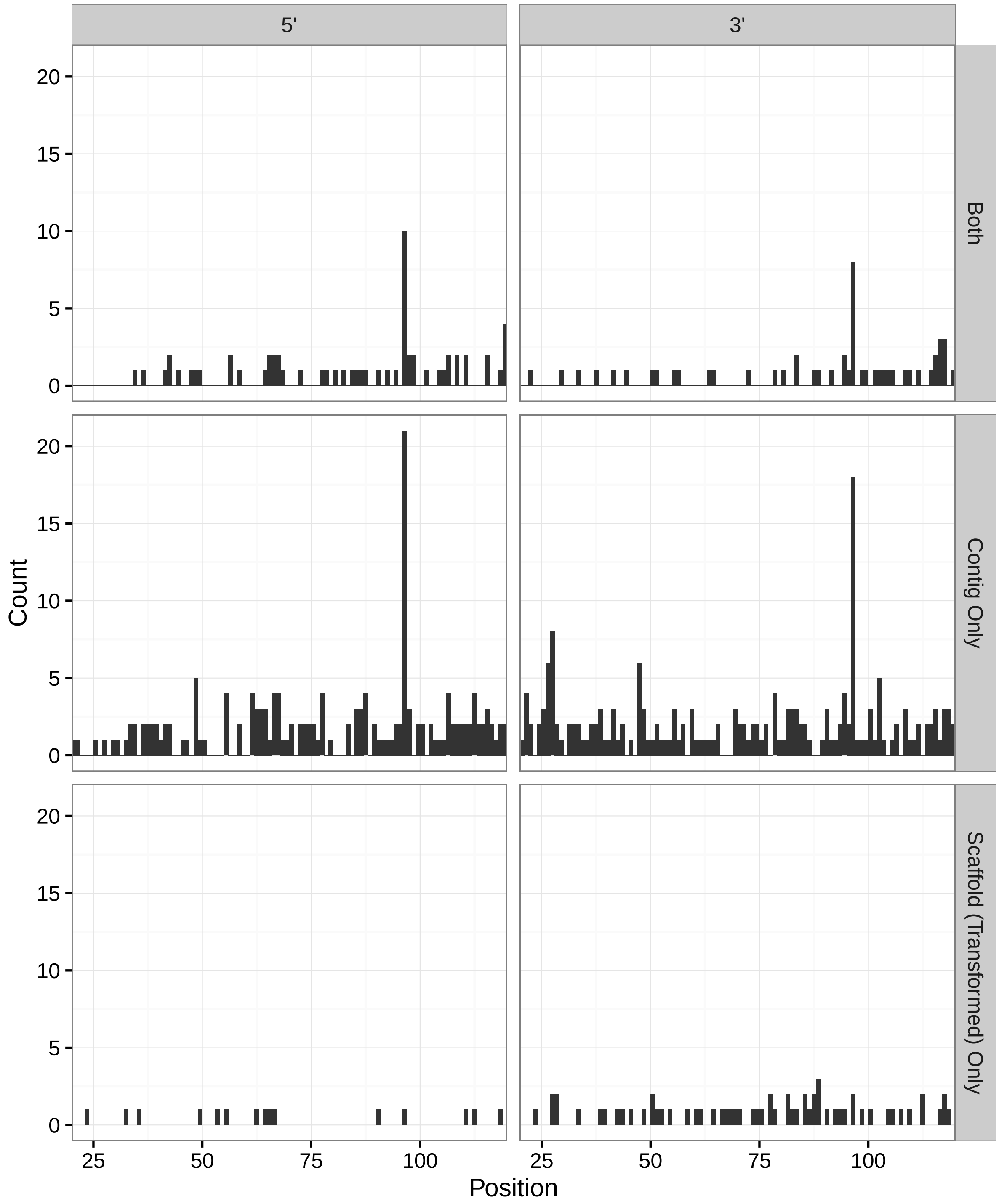

Figure S26: Distribution of positions for SNPs called against contigs only, scaffolds only, or both in the **Sim_allp** assembly. The coordinates of scaffold SNPs were transformed into contig coordinates.

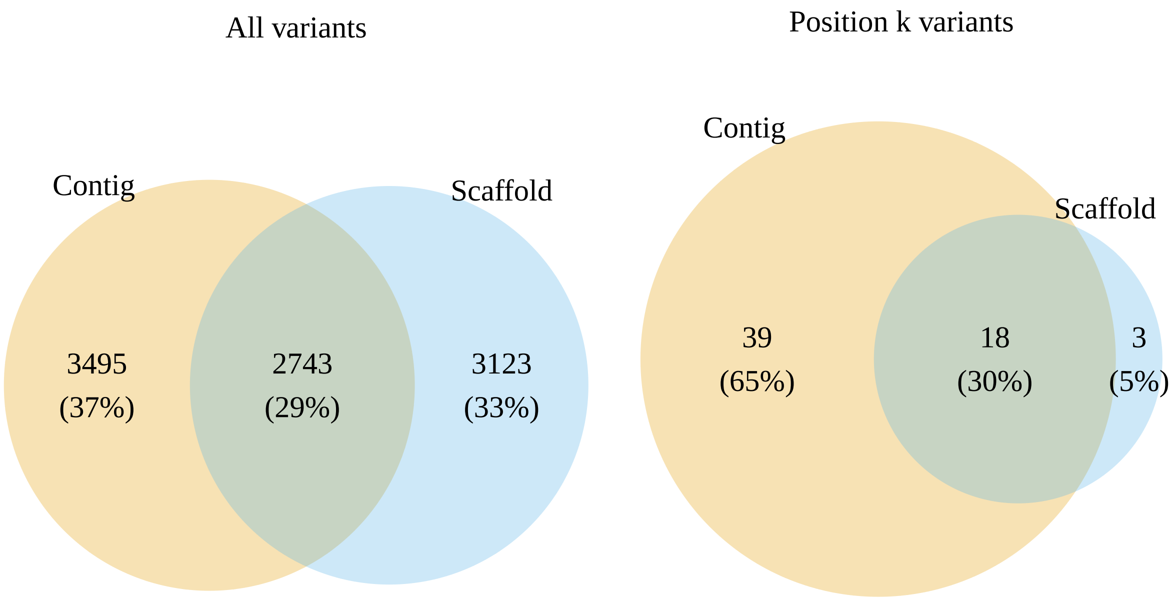

Figure S27: Intersection between SNPs called against **Sim_allp** contigs and scaffolds. The coordinates of scaffold SNPs were transformed into contig coordinates.

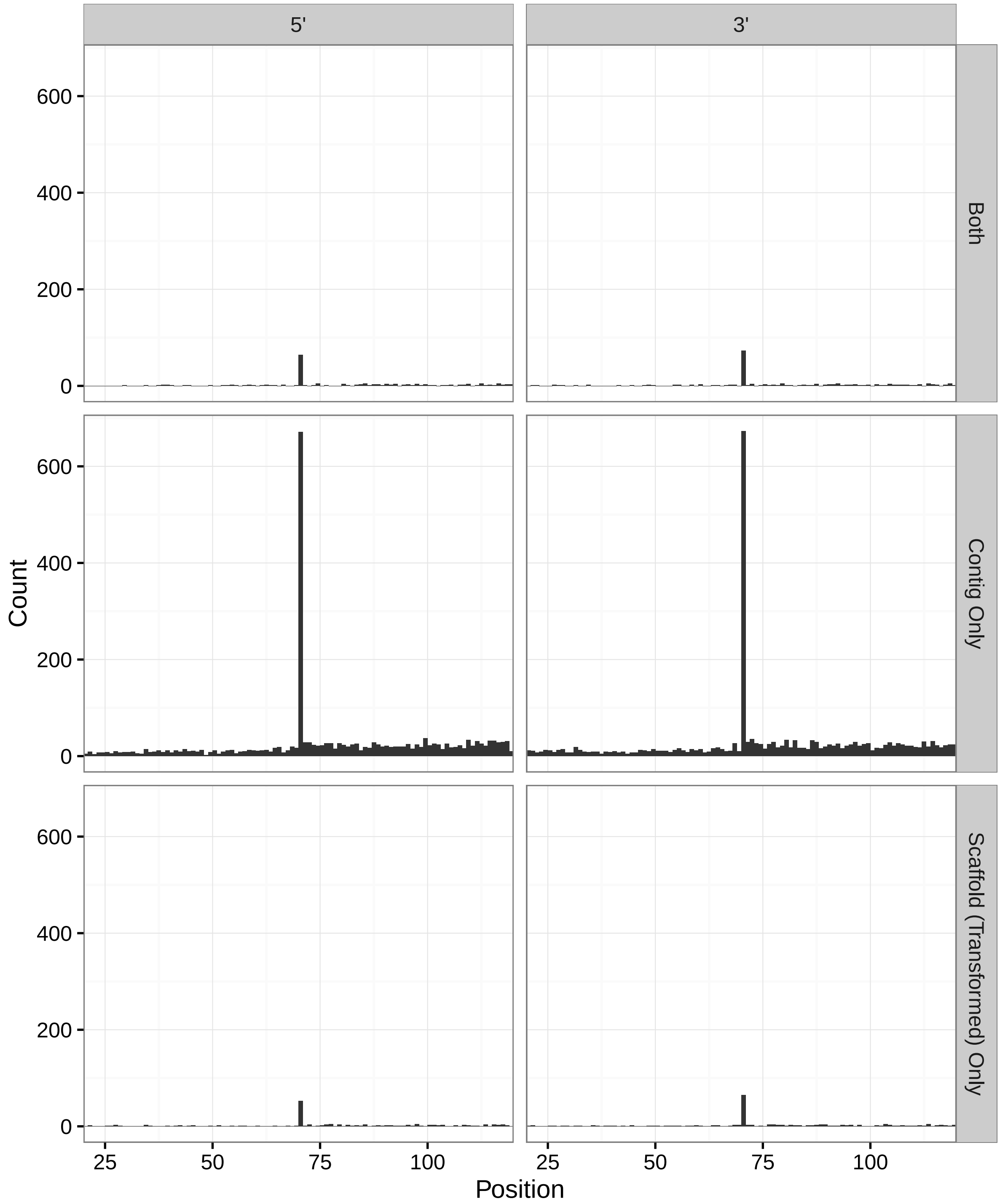

Figure S28: Distribution of positions for SNPs called against contigs only, scaffolds only, or both in the **Sim_soap_K69** assembly. The coordinates of scaffold SNPs were transformed into contig coordinates.

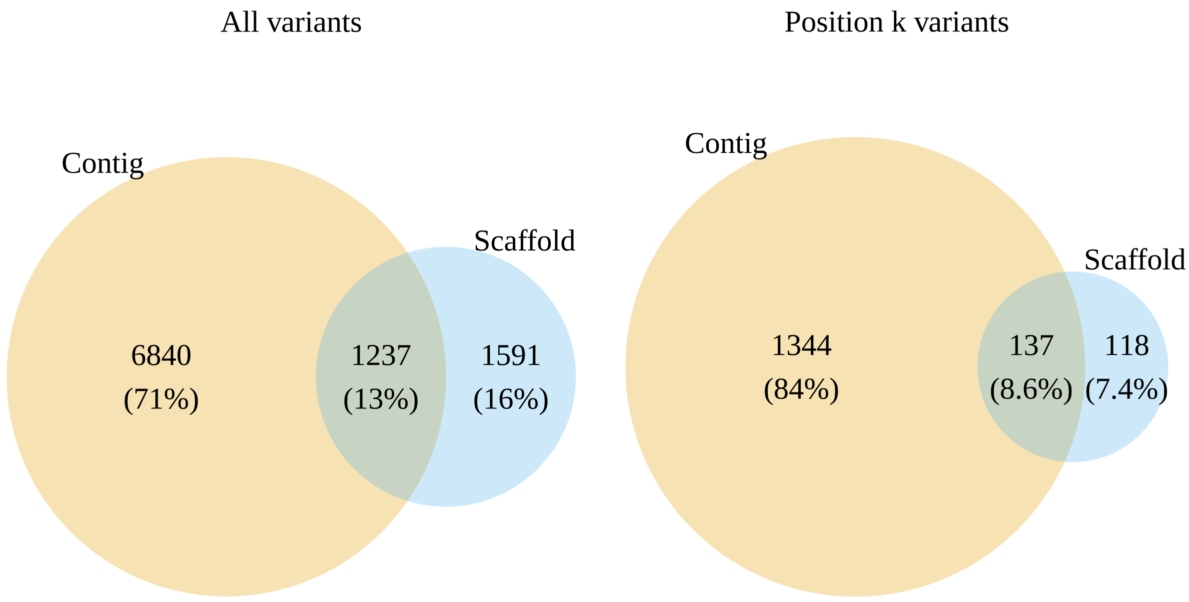

Figure S29: Intersection between SNPs called against **Sim_soap_K69** contigs and scaffolds. The coordinates of scaffold SNPs were transformed into contig coordinates.

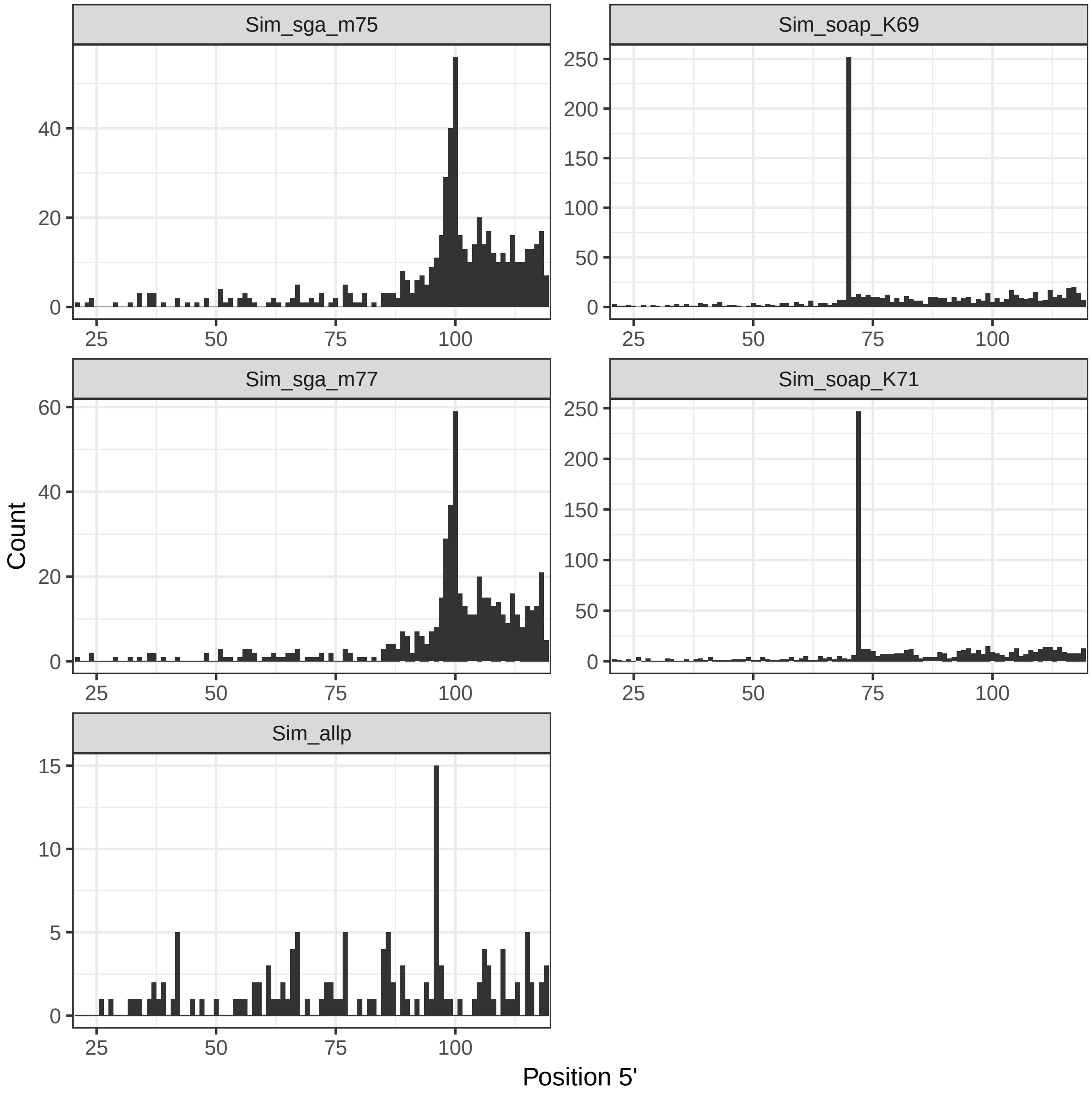

Figure S30: Distribution of SNP positions obtained with NextGenMap and GATK at the 5´ end of **contigs** in the simulated data set.

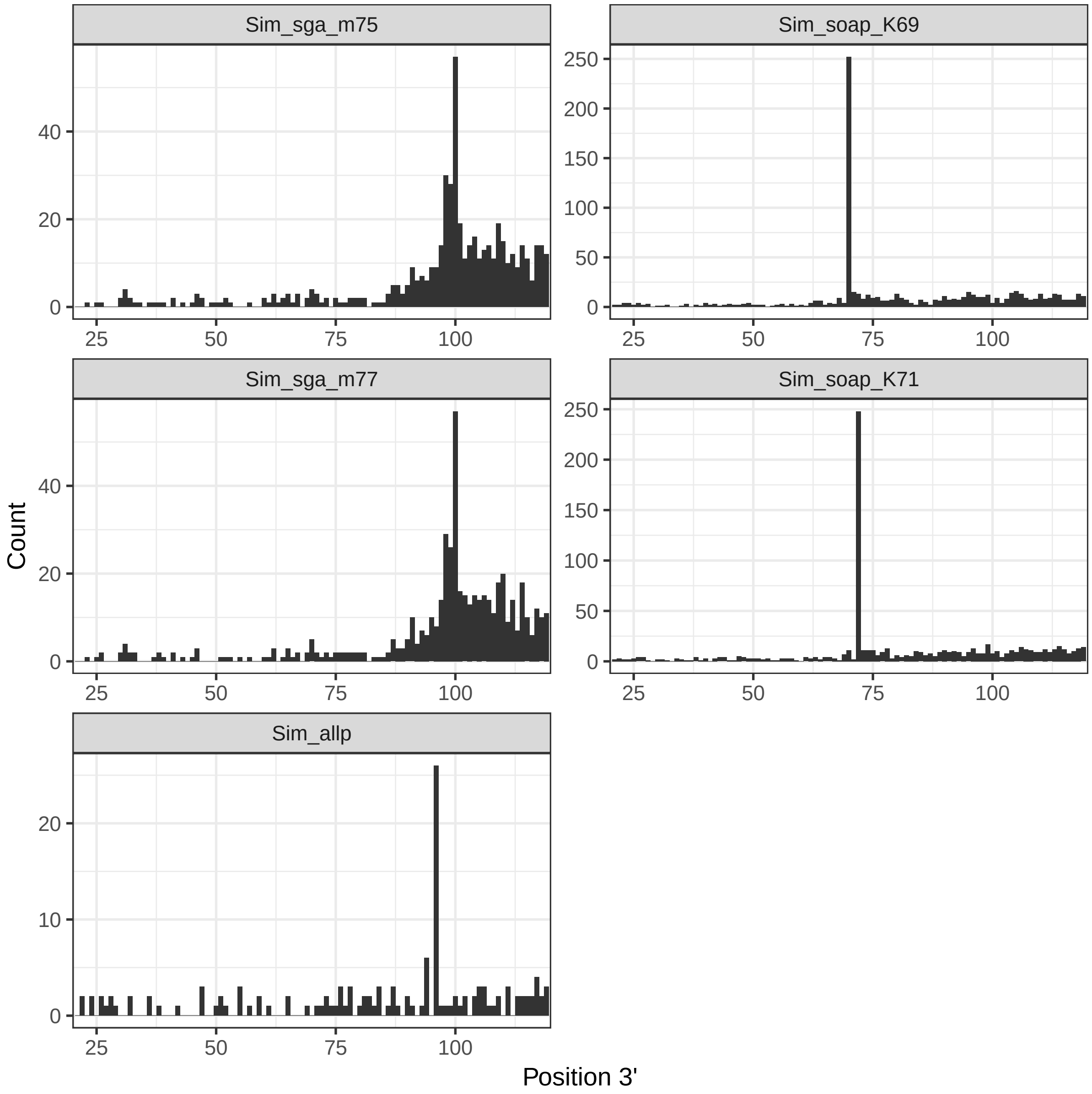

Figure S31: Distribution of SNP positions obtained with NextGenMap and GATK at the 3´ end of **contigs** in the simulated data set.

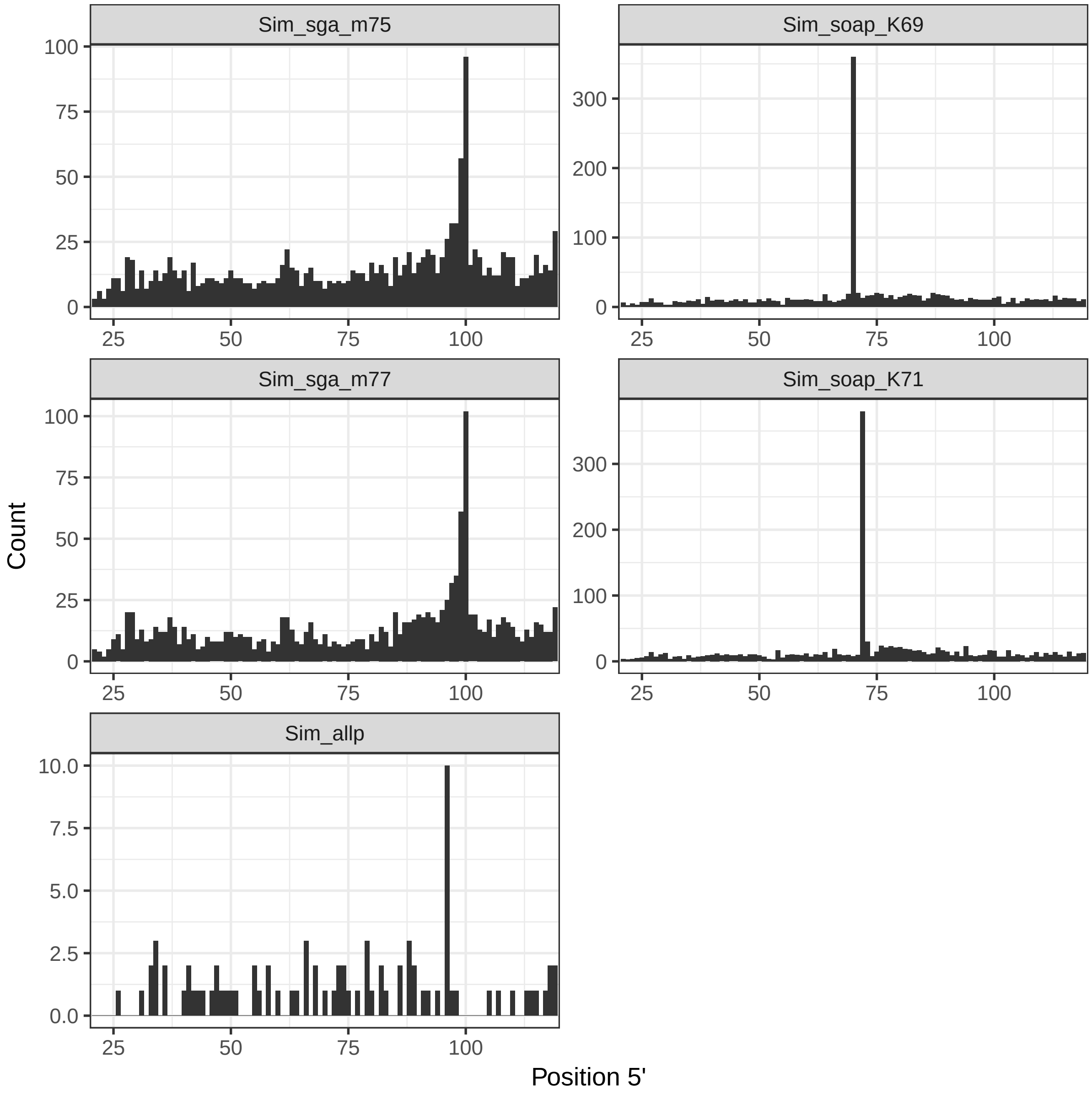

Figure S32: Distribution of SNP positions obtained with GSNAP and GATK at the 5´ end of **contigs** in the simulated data set.

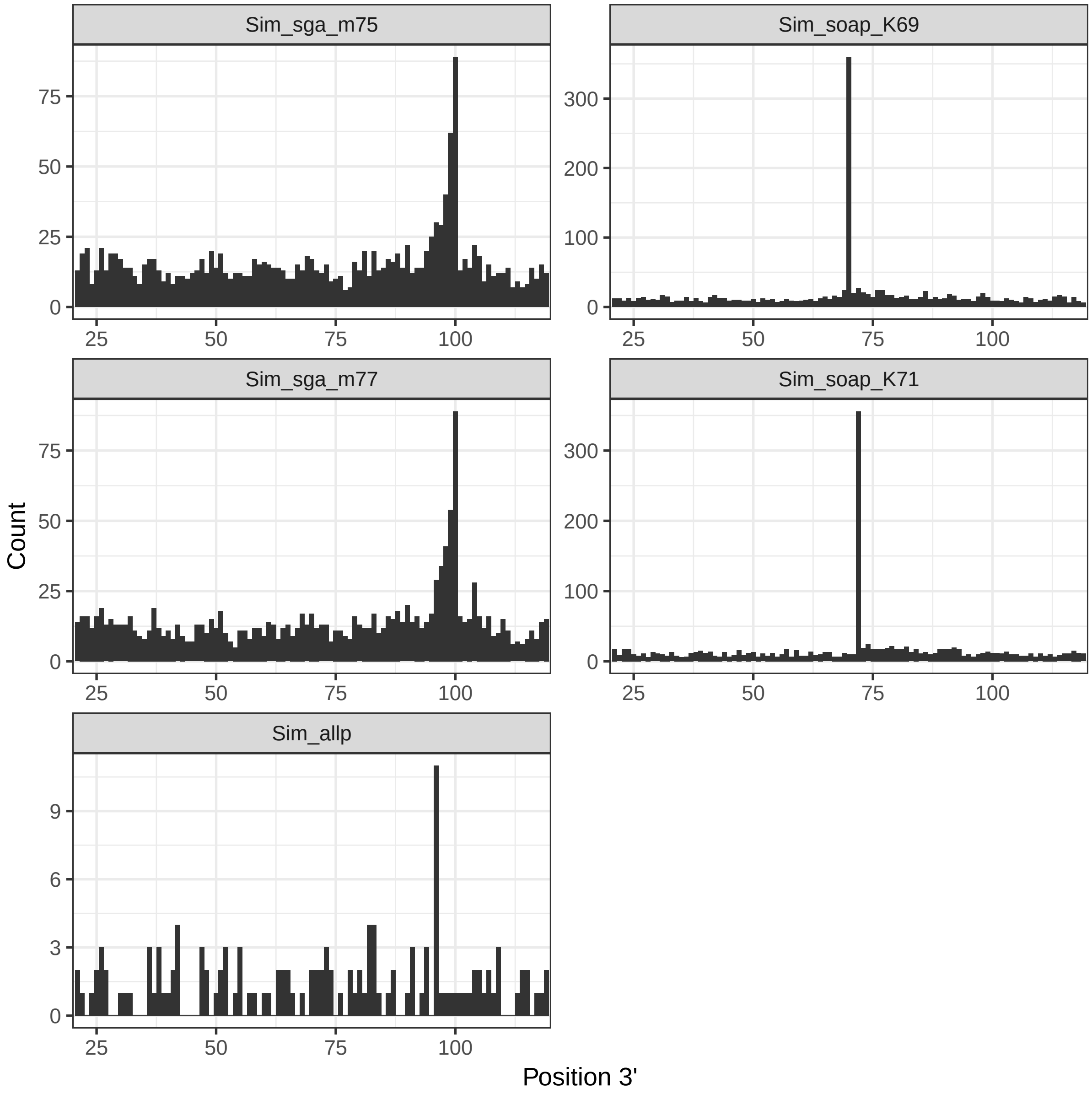

Figure S33: Distribution of SNP positions obtained with GSNAP and GATK at the 3´ end of **contigs** in the simulated data set.

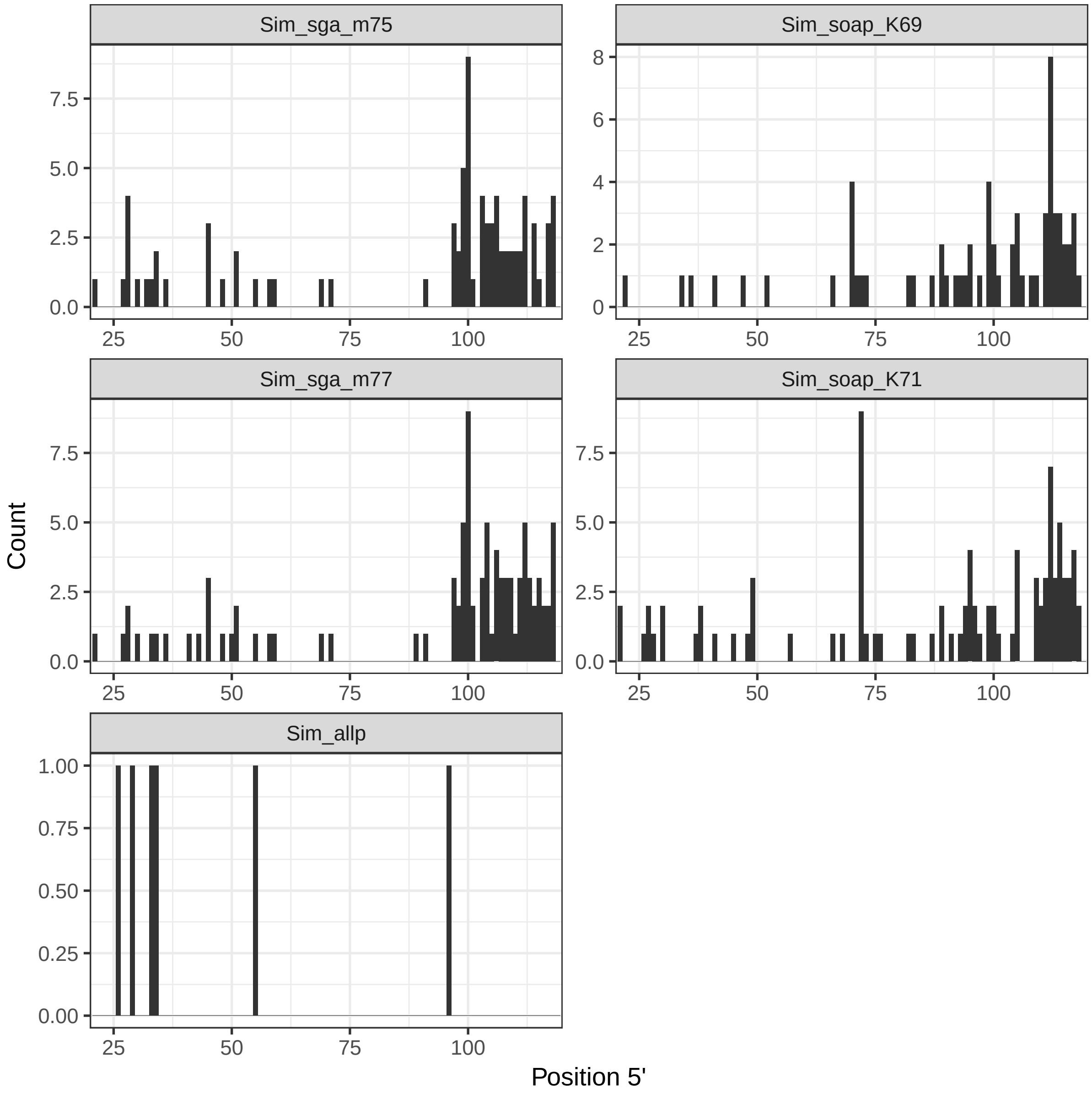

Figure S34: Distribution of SNP positions obtained with Bowtie2 and GATK at the 5´ end of **contigs** in the simulated data set.

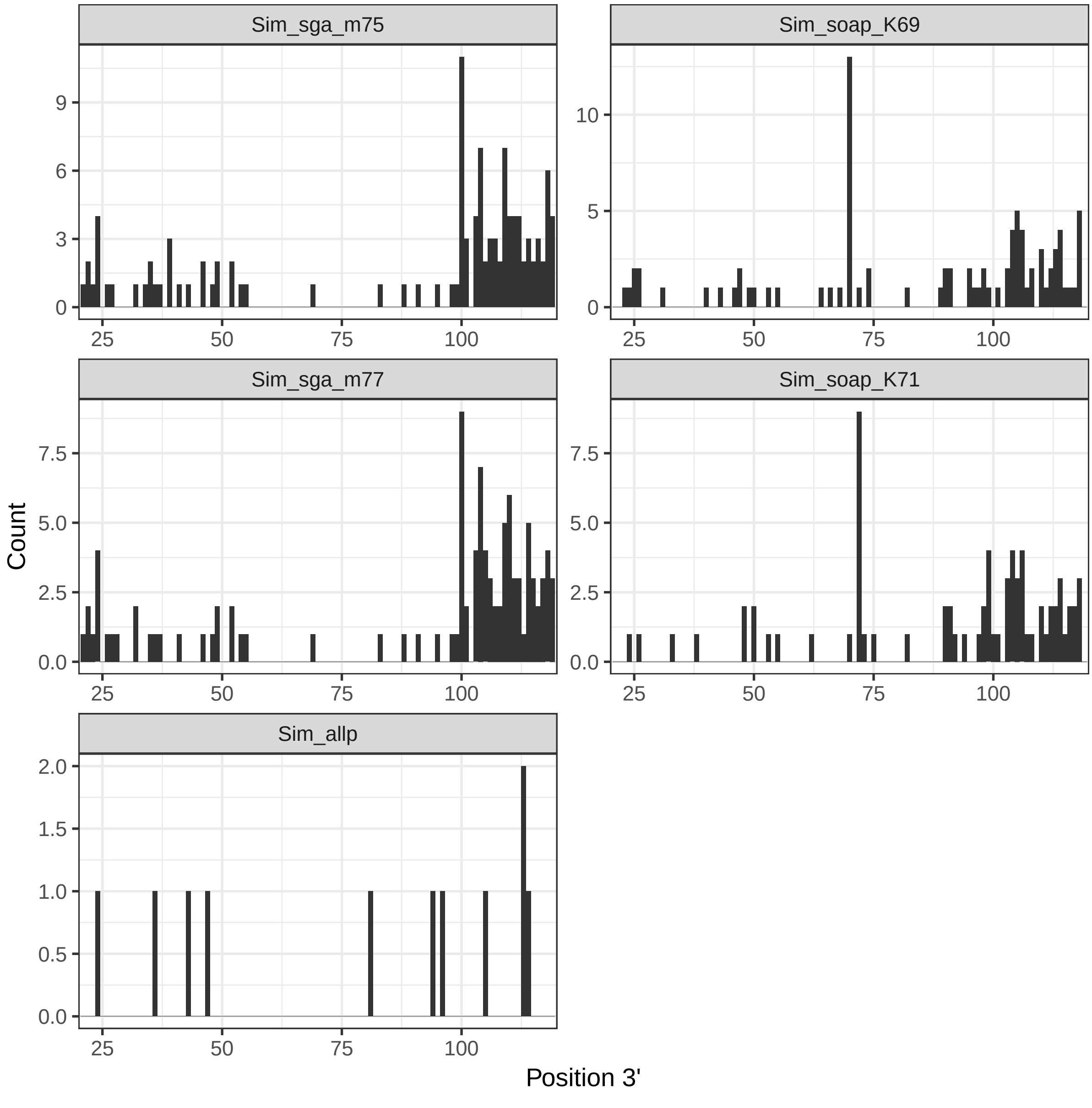

Figure S35: Distribution of SNP positions obtained with Bowtie2 and GATK at the 3´ end of **contigs** in the simulated data set.

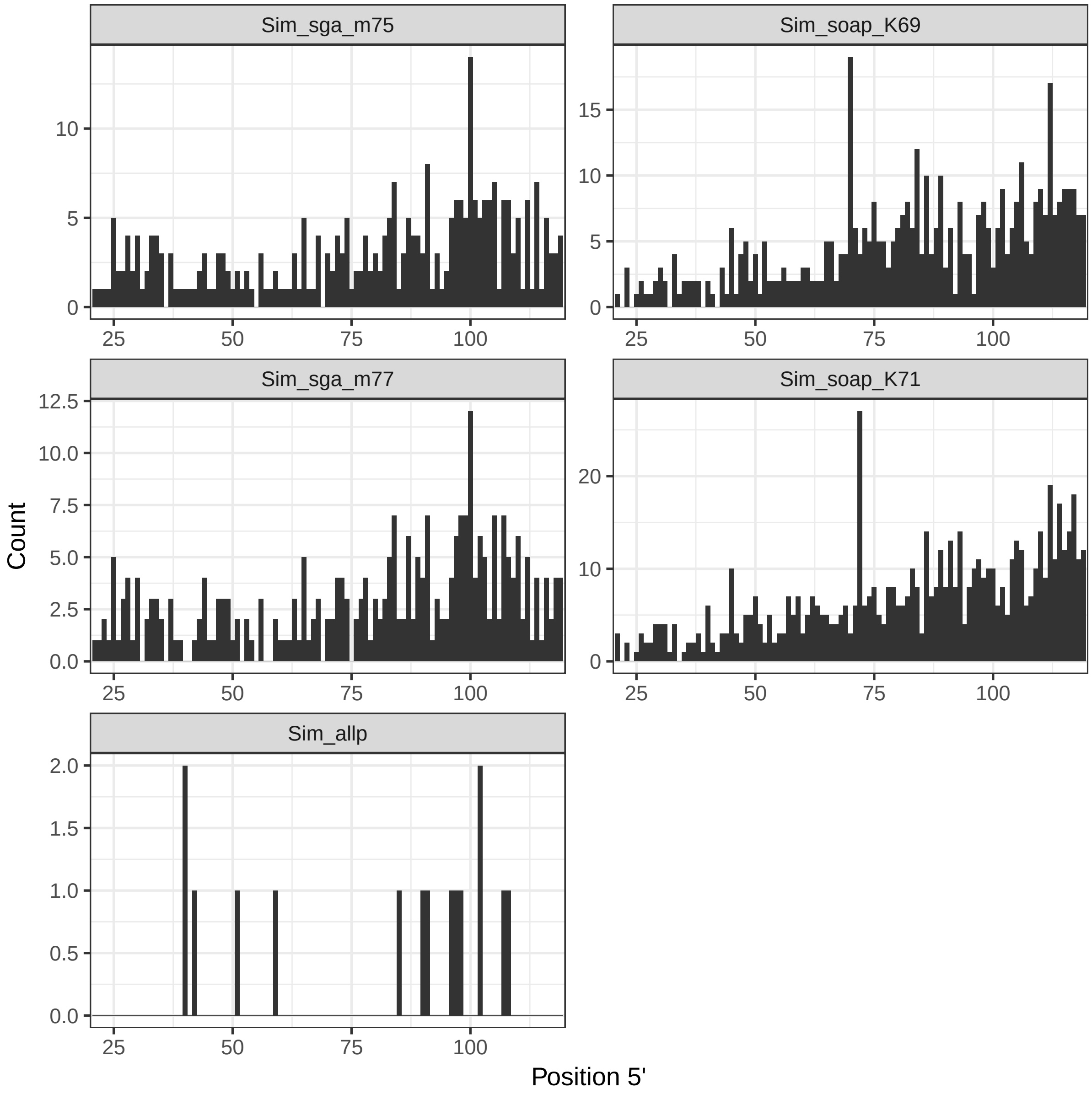

Figure S36: Distribution of SNP positions obtained with Bowtie2 and GATK at the 5´ end of **contigs** in the simulated resequencing analysis.

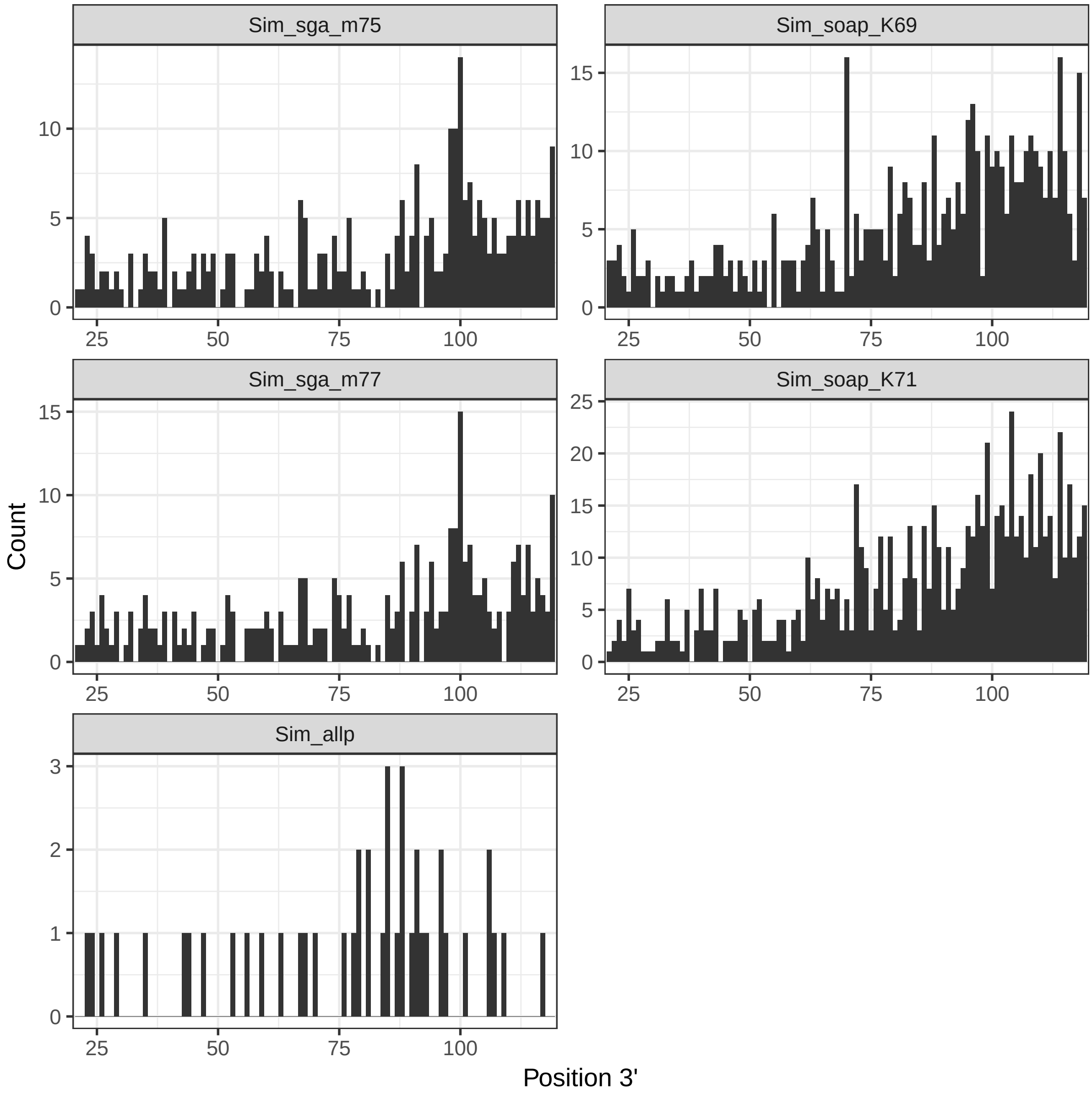

Figure S37: Distribution of SNP positions obtained with Bowtie2 and GATK at the 3´ end of **contigs** in the simulated resequencing analysis.

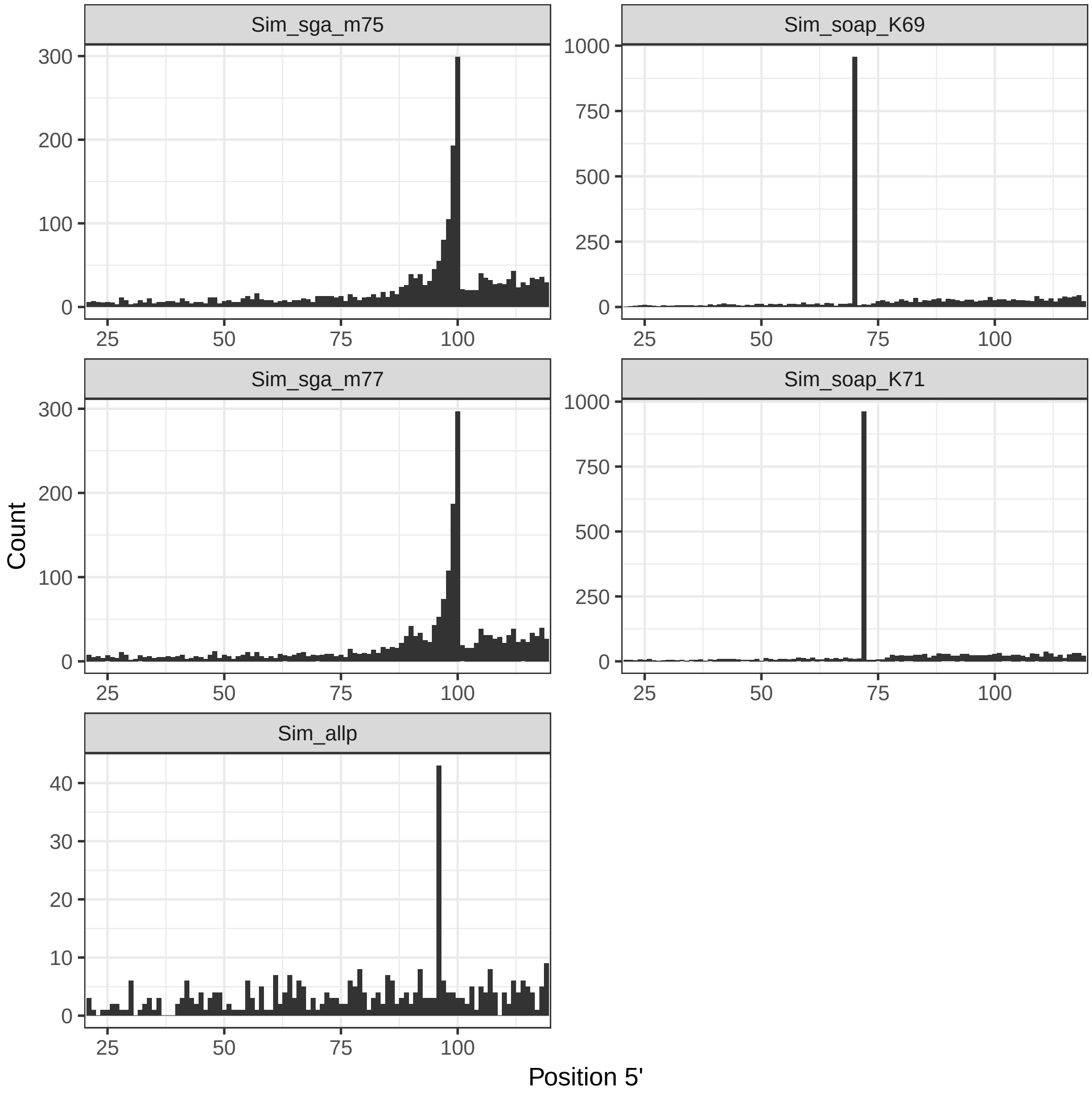

Figure S38: Distribution of SNP positions obtained with BWA and FreeBayes at the 5´ end of **contigs** in the simulated data set.

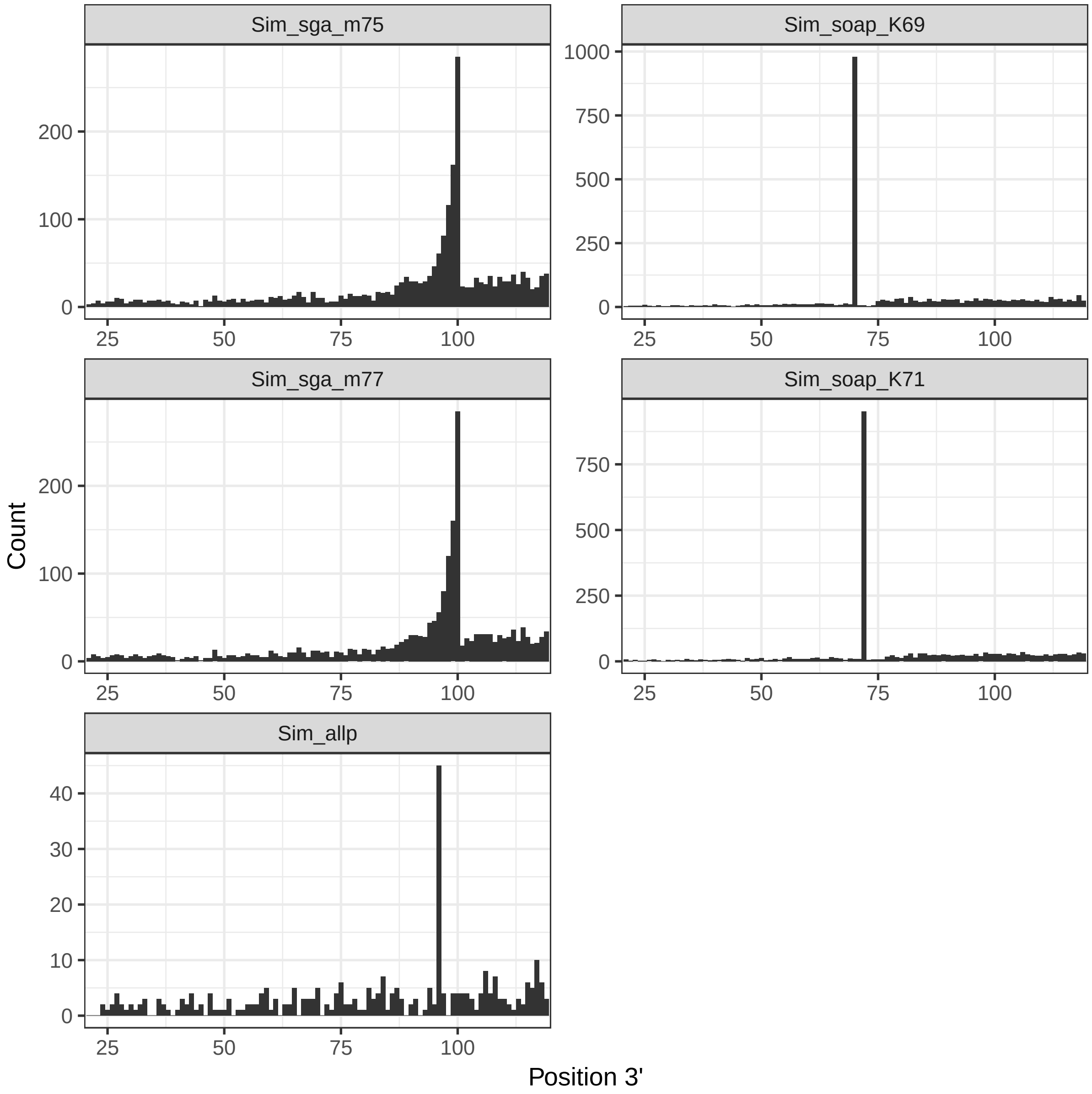

Figure S39: Distribution of SNP positions obtained with BWA and FreeBayes at the 3´ end of **contigs** in the simulated data set.

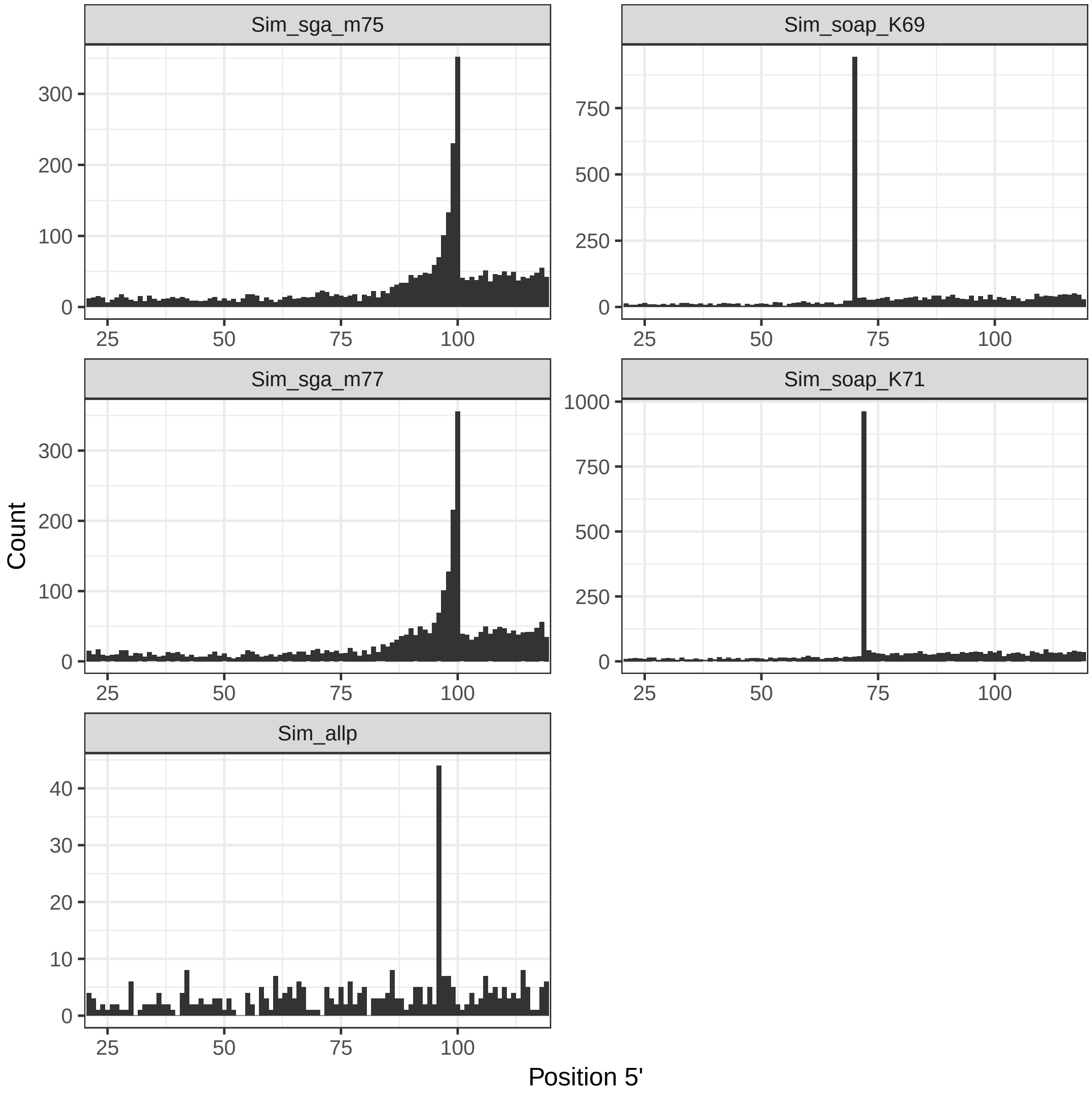

Figure S40: Distribution of SNP positions obtained with BWA and Samtools mpileup at the 5´ end of **contigs** in the simulated data set.

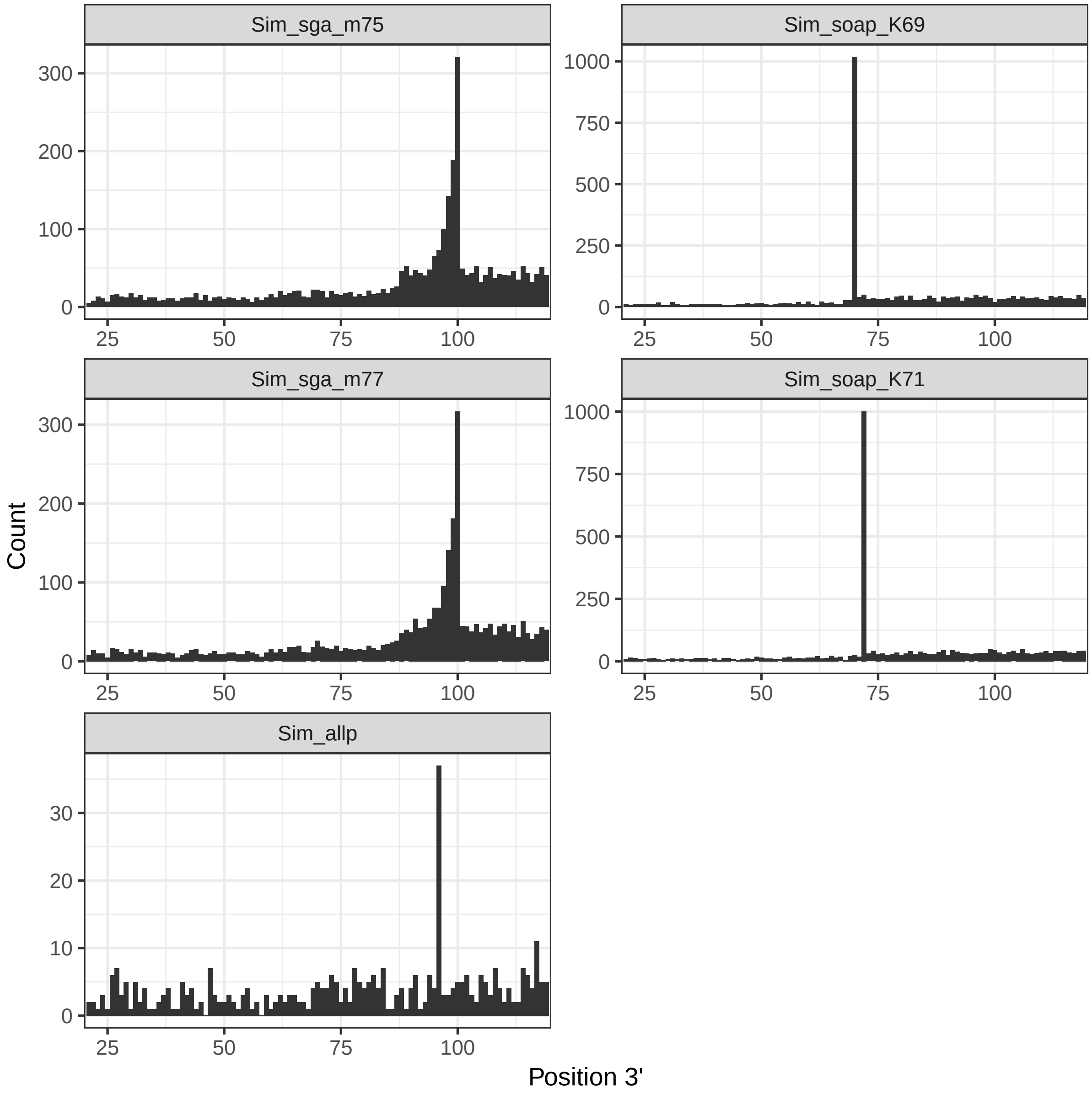

Figure S41: Distribution of SNP positions obtained with BWA and Samtools mpileup at the 3´ end of **contigs** in the simulated data set.

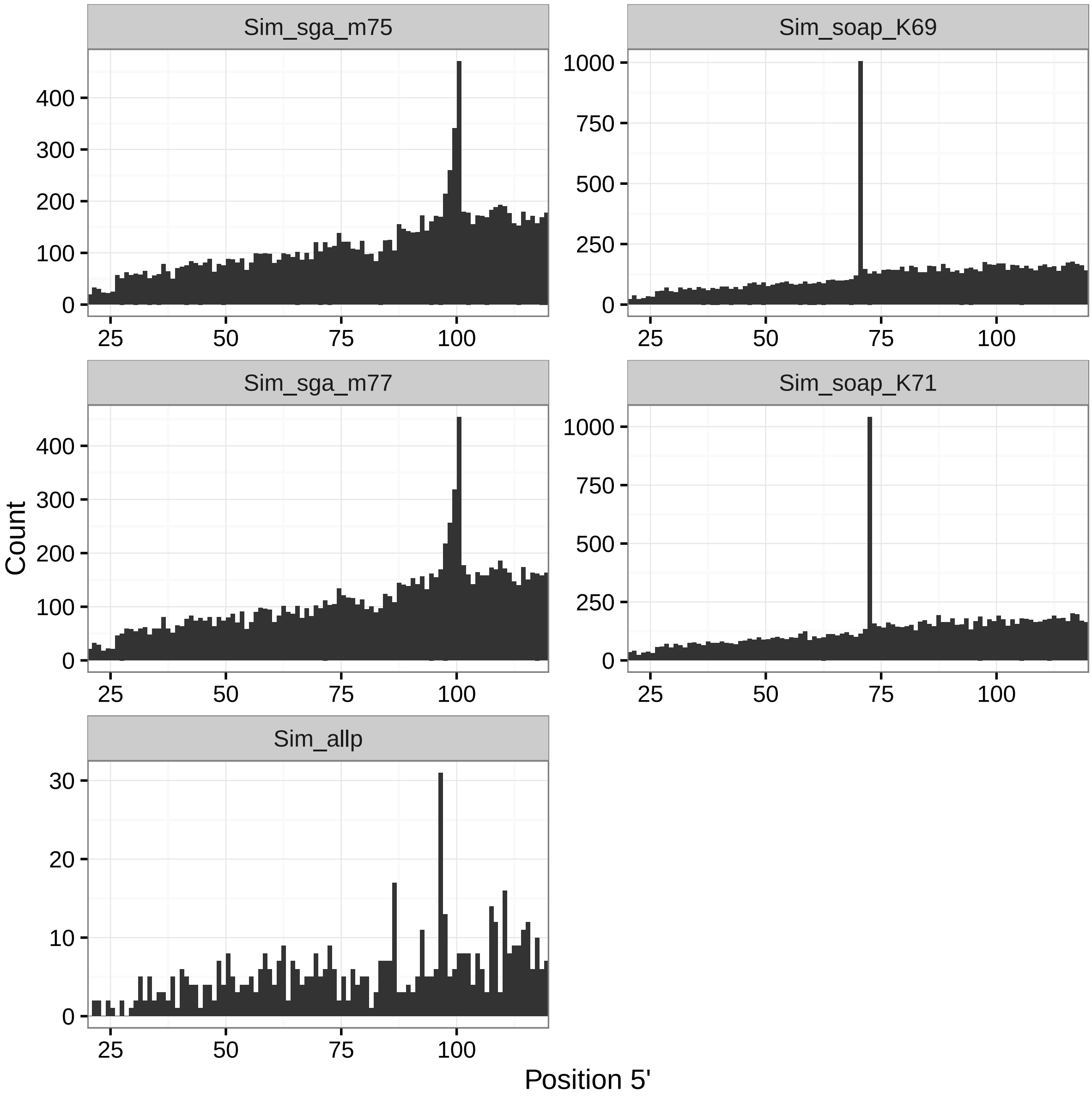

Figure S42: Distribution of SNP positions at the 5´ end of **contigs** in the simulated resequencing analysis.

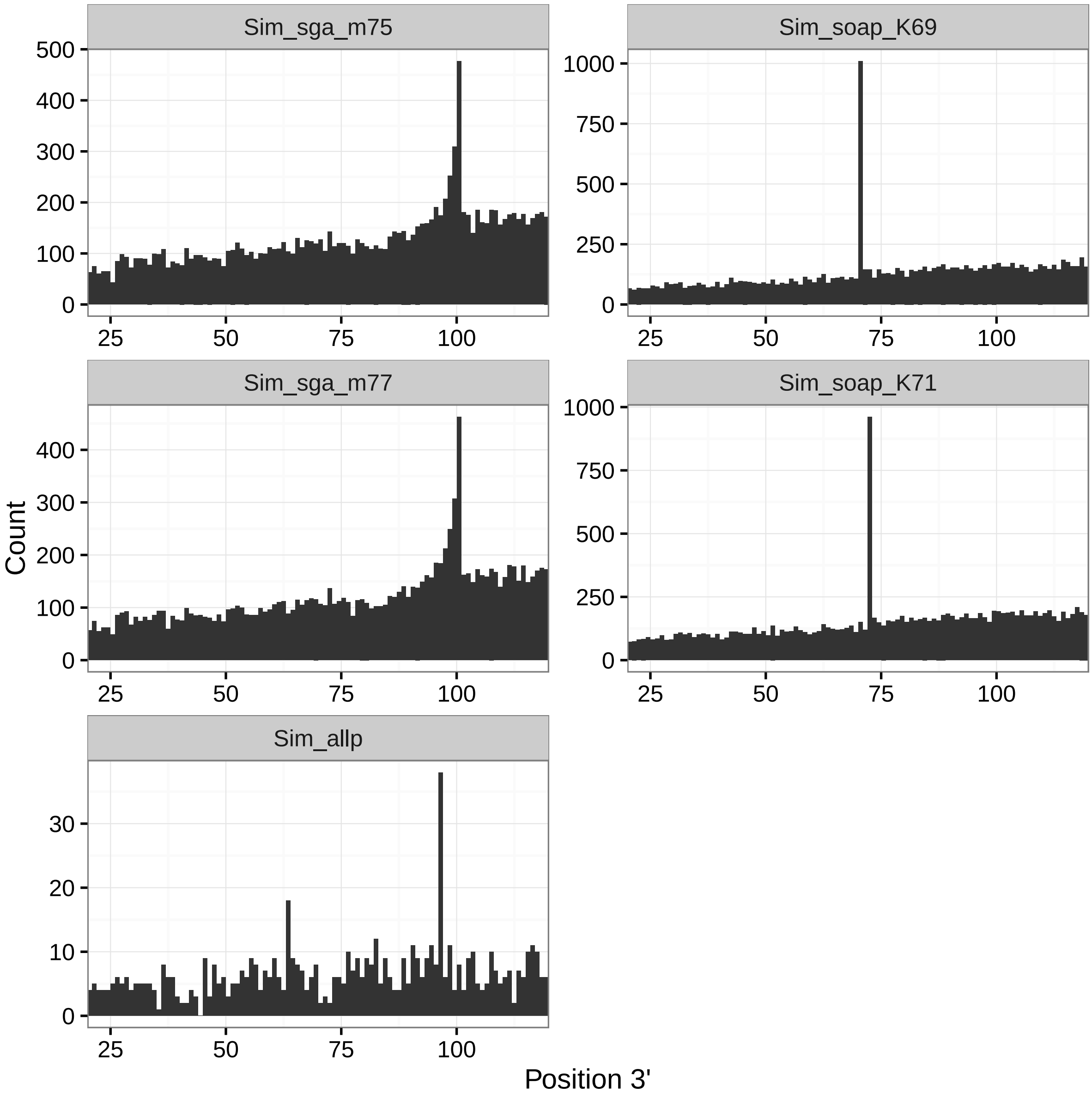

Figure S43: Distribution of SNP positions at the 3´ end of **contigs** in the simulated resequencing analysis.

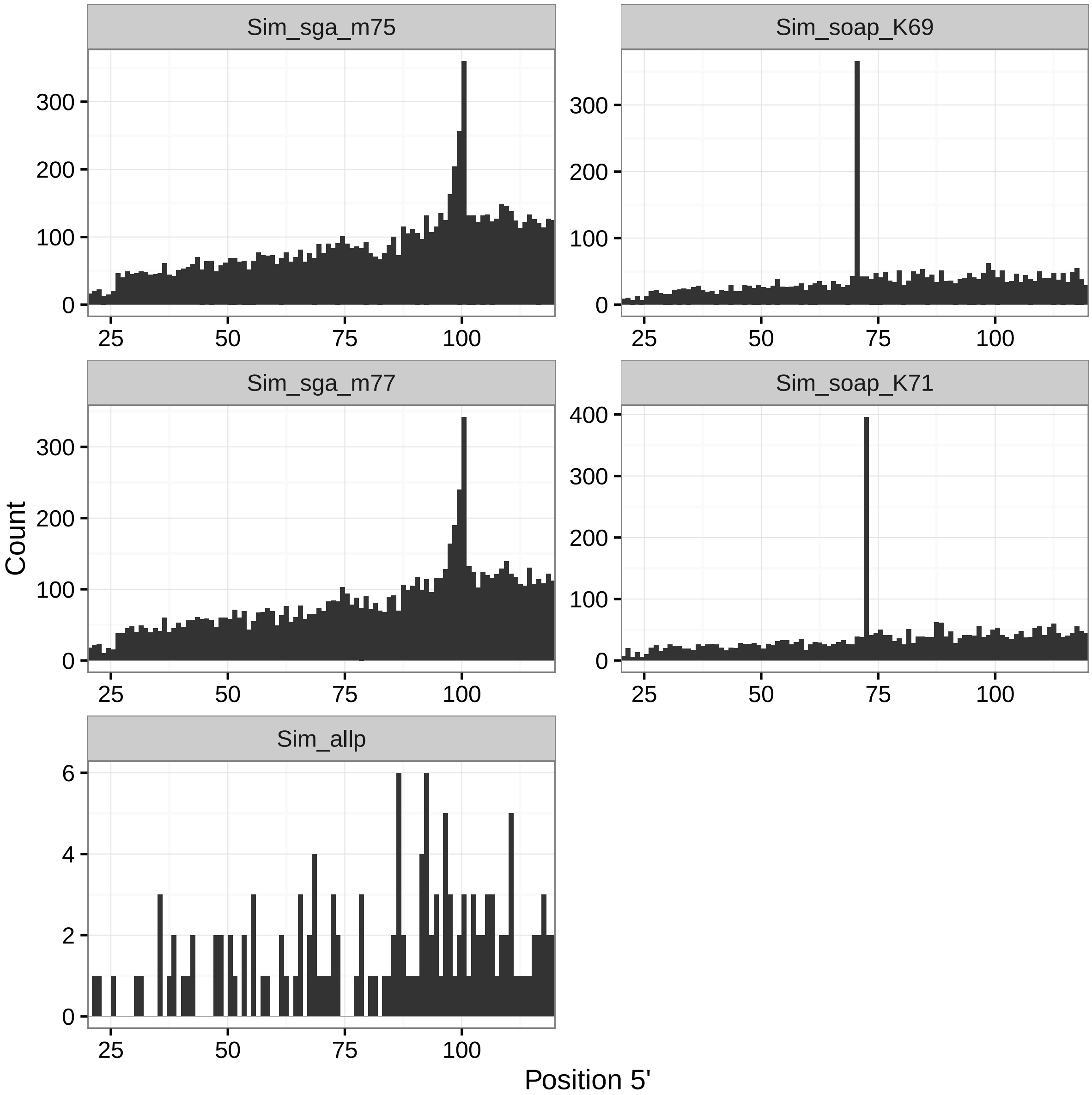

Figure S44: Distribution of SNP positions at the 5´ end of **scaffolds** in the simulated resequencing analysis.

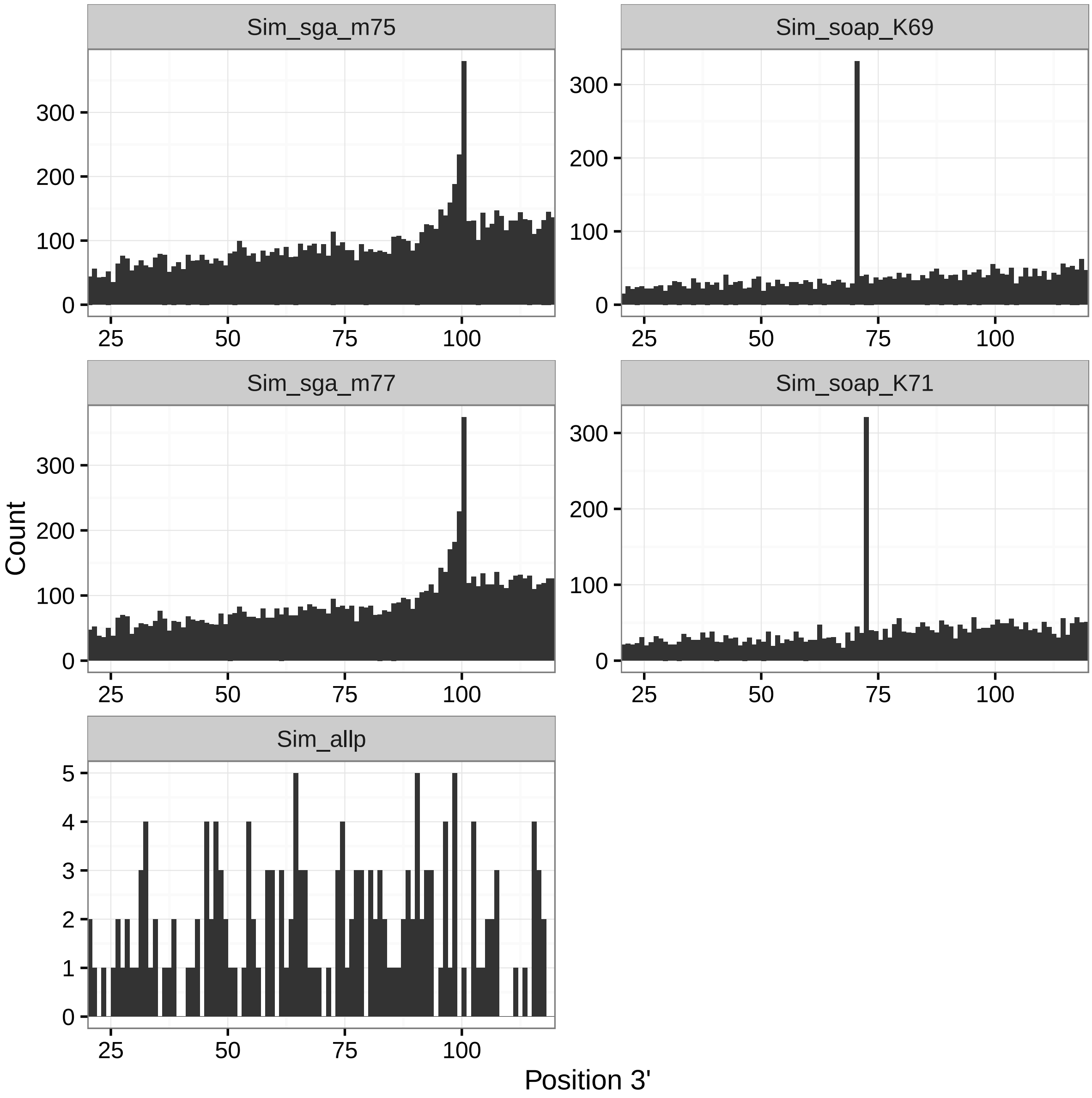

Figure S45: Distribution of SNP positions at the 3´ end of **scaffolds** in the simulated resequencing analysis.

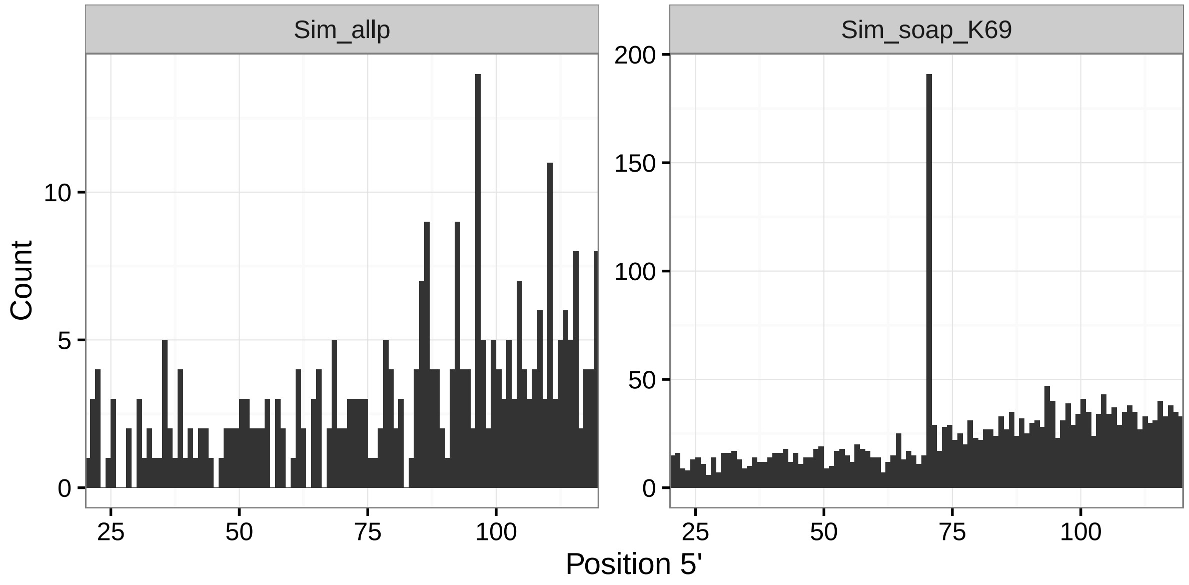

Figure S46: Distribution of SNP positions **transformed** from scaffold to contig coordinates at the 5´ end of contigs in the simulated resequencing analysis.

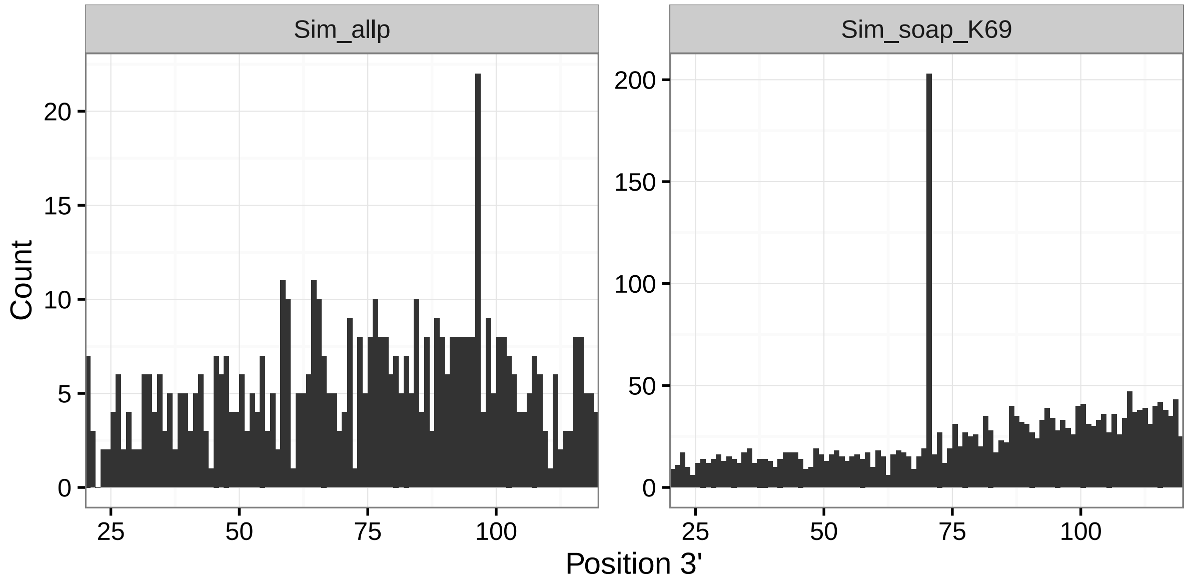

Figure S47: Distribution of SNP positions **transformed** from scaffold to contig coordinates at the 3´ end of contigs in the simulated resequencing analysis.

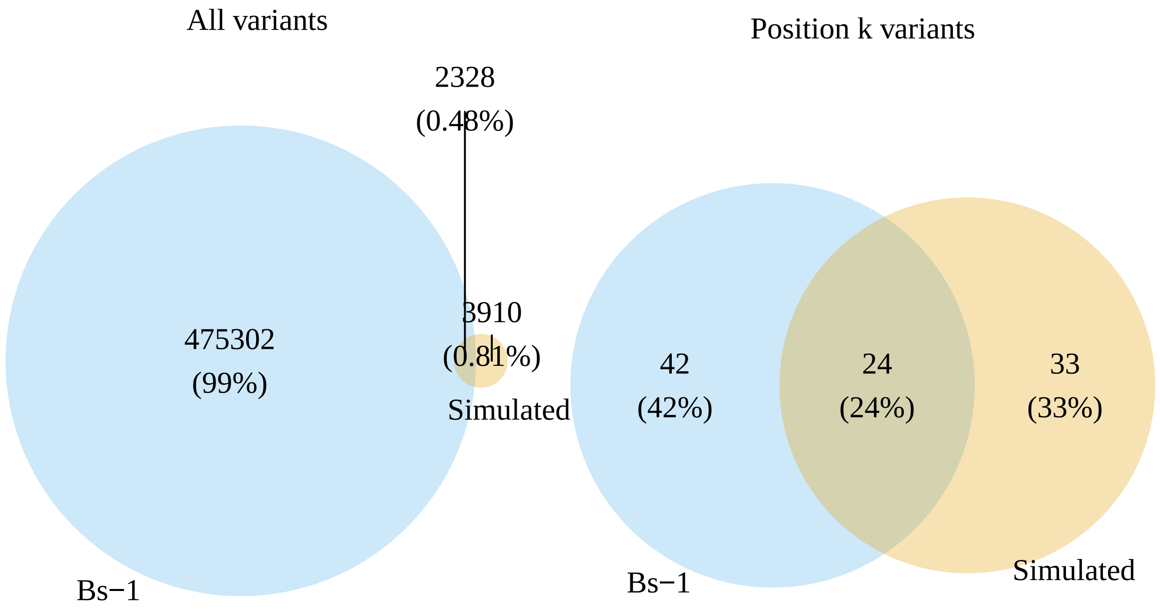

Figure S48: Intersection between SNPs called against **Sim_allp contigs** with the actual *A. thaliana* Bs-1 reads or simulated reads.

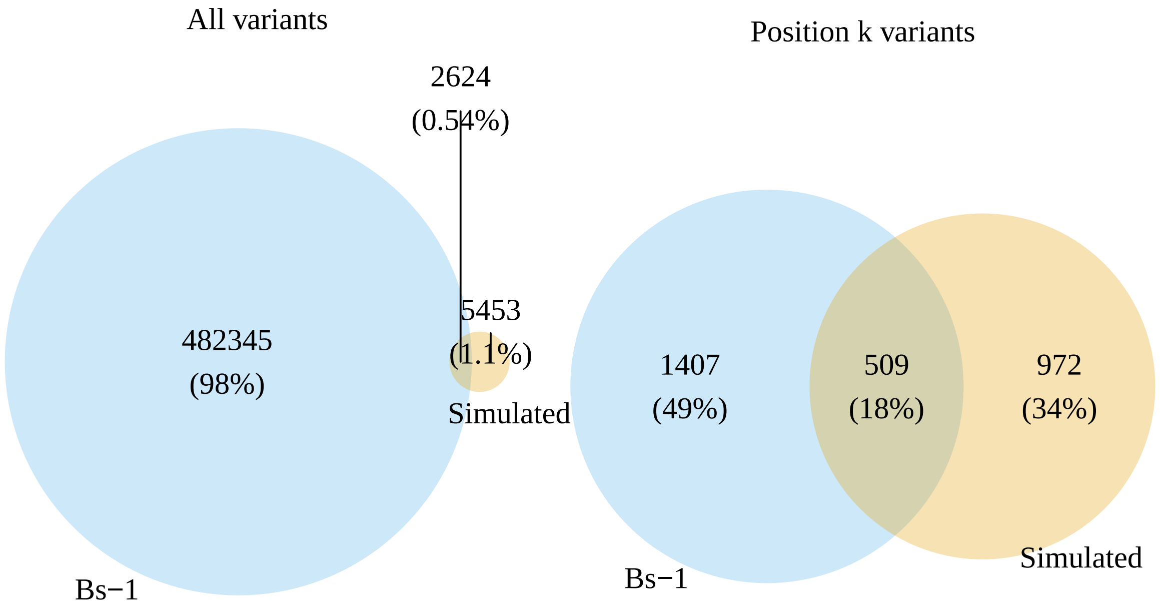

Figure S49: Intersection between SNPs called against **Sim_soap_K69 contigs** with the actual *A. thaliana* Bs-1 reads or our simulated reads.

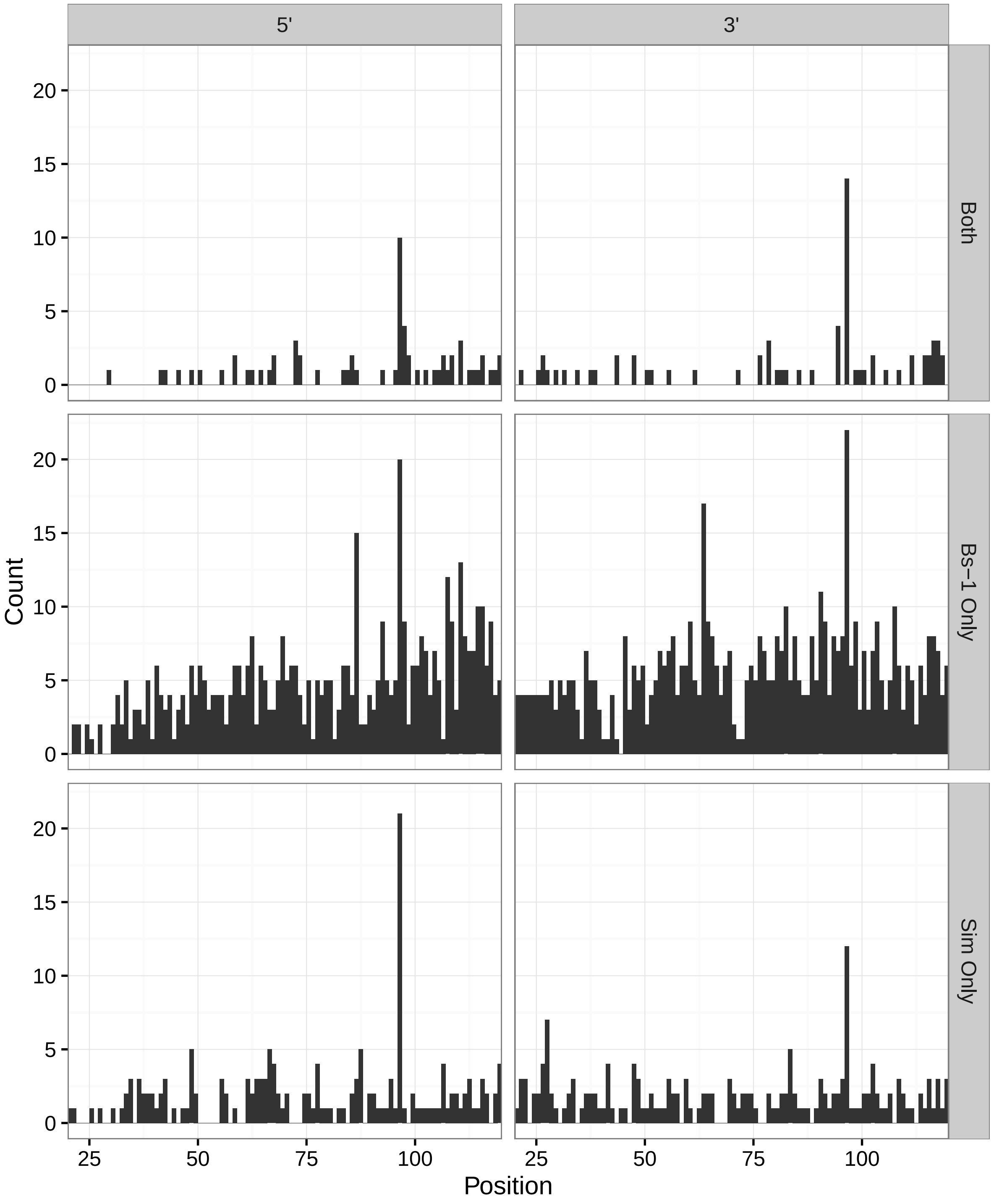

Figure S50: Distribution of positions for SNPs called against **Sim_allp contigs** using the actual *A. thaliana* Bs-1 reads or our simulated reads. The ‘Sim only’ row shows SNPs called only with the simulated reads, ‘Bs-1 only’ row exhibits SNPs unique to the Bs-1 alignments, while ‘Both’ row displays SNPs called individually with both Bs-1 and simulated reads.

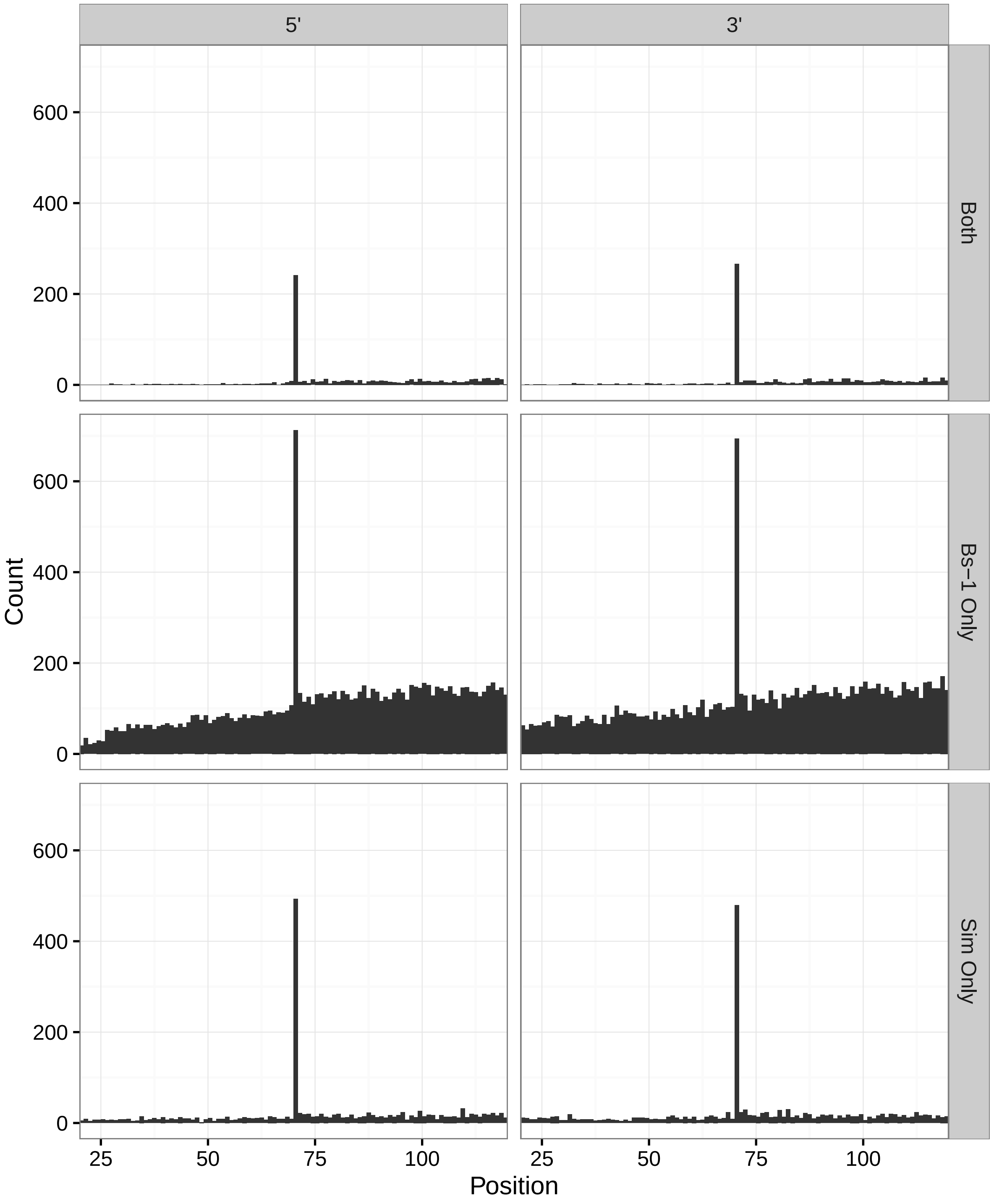

Figure S51: Distribution of positions for SNPs called against **Sim_soap_K69 contigs** using the actual *A. thaliana* Bs-1 reads or our simulated reads. The 'Sim only' row shows SNPs called only with the simulated reads, 'Bs-1 only' row exhibits SNPs unique to the Bs-1 alignments, while 'Both' row displays SNPs called individually with both Bs-1 and simulated reads.

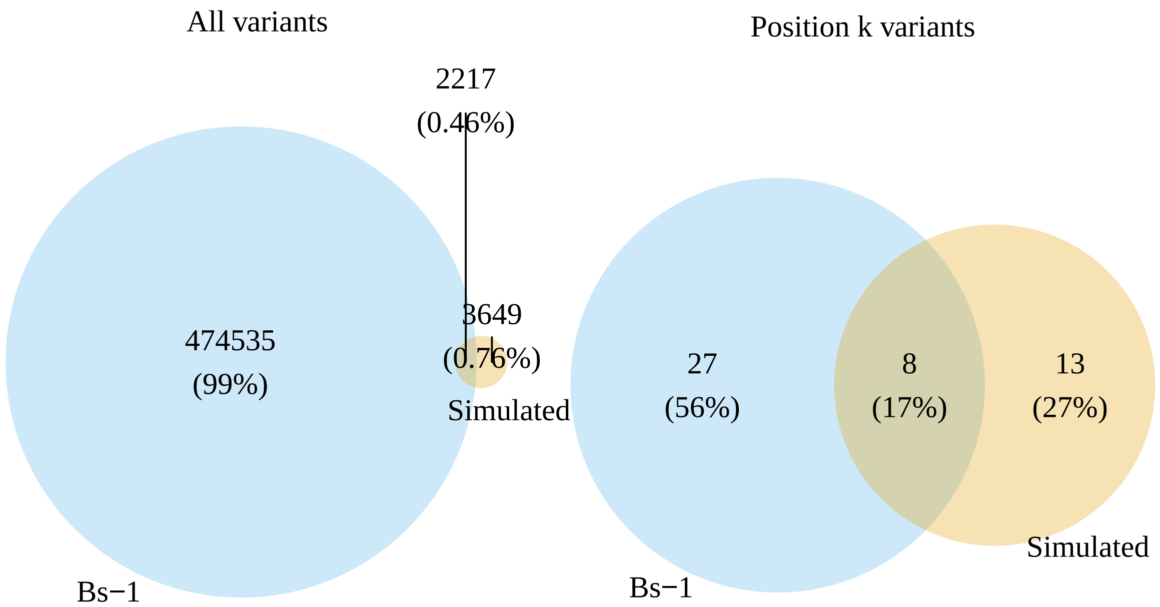

Figure S52: Intersection between SNPs called against **Sim_allp scaffolds** with the actual *A. thaliana* Bs-1 reads or our simulated reads. Scaffold coordinates were transformed into the contig coordinates.

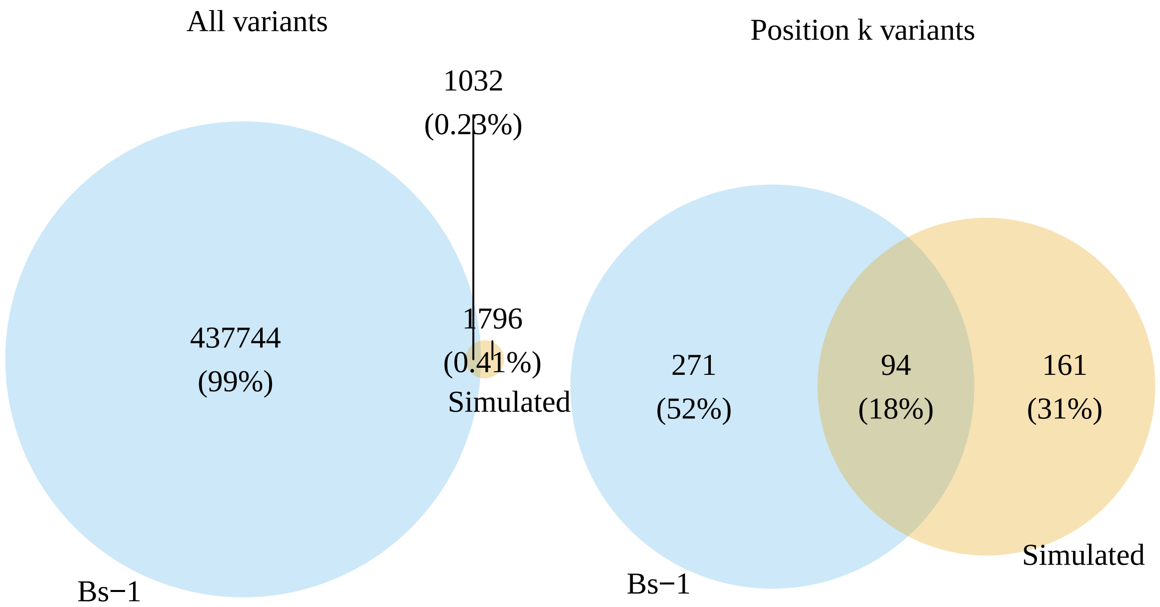

Figure S53: Intersection between SNPs called against **Sim_soap_K69 scaffolds** with the actual *A. thaliana* Bs-1 reads or our simulated reads. Scaffold coordinates were transformed into the contig coordinates.

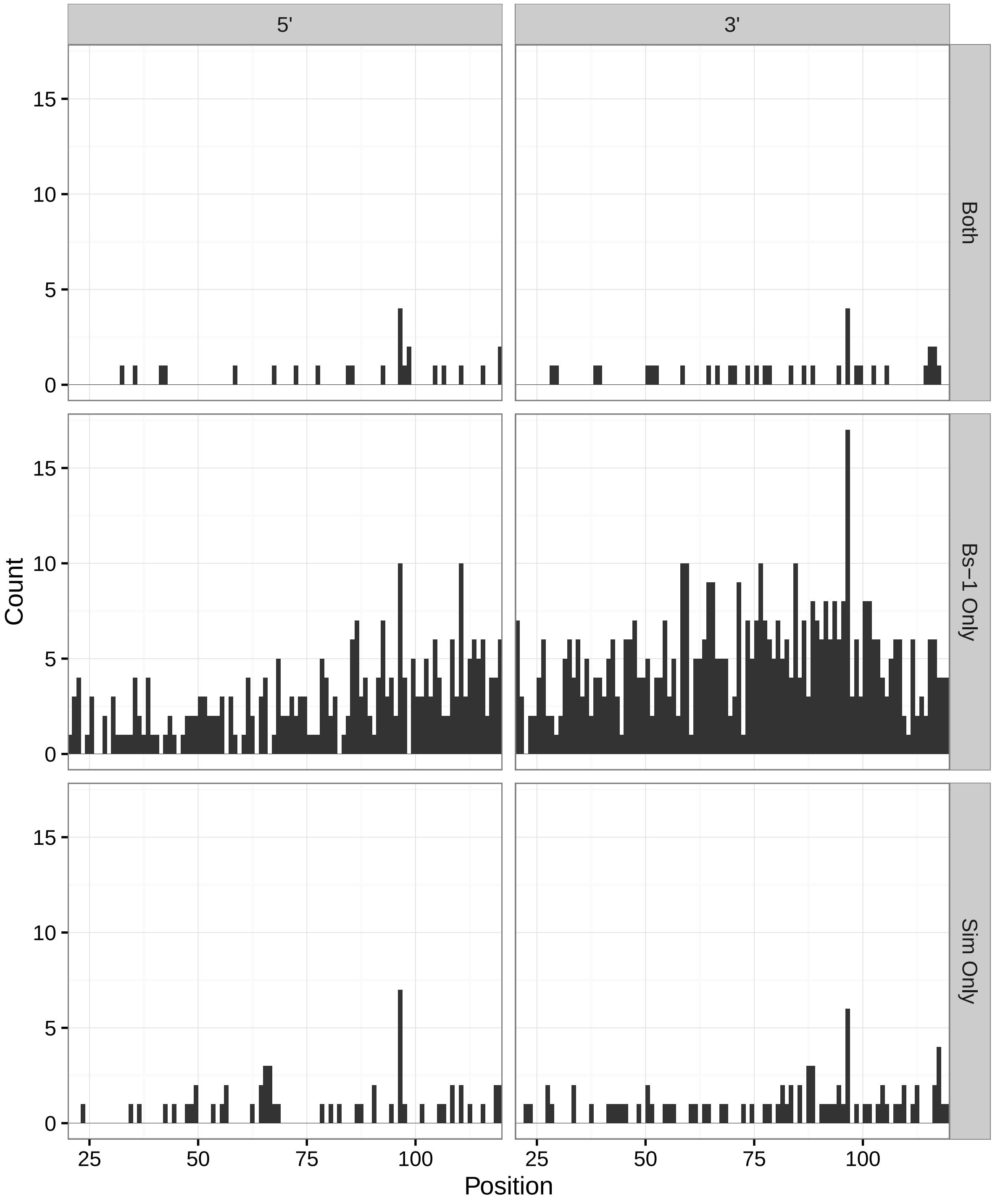

Figure S54: Distribution of positions for SNPs called against **Sim_allp scaffolds** using the actual *A. thaliana* Bs-1 reads or our simulated reads. The 'Sim only' row shows SNPs called only with the simulated reads, 'Bs-1 only' row exhibits SNPs unique to the Bs-1 alignments, while 'Both' row displays SNPs called individually with both Bs-1 and simulated reads. Scaffold coordinates were transformed into the contig coordinates.

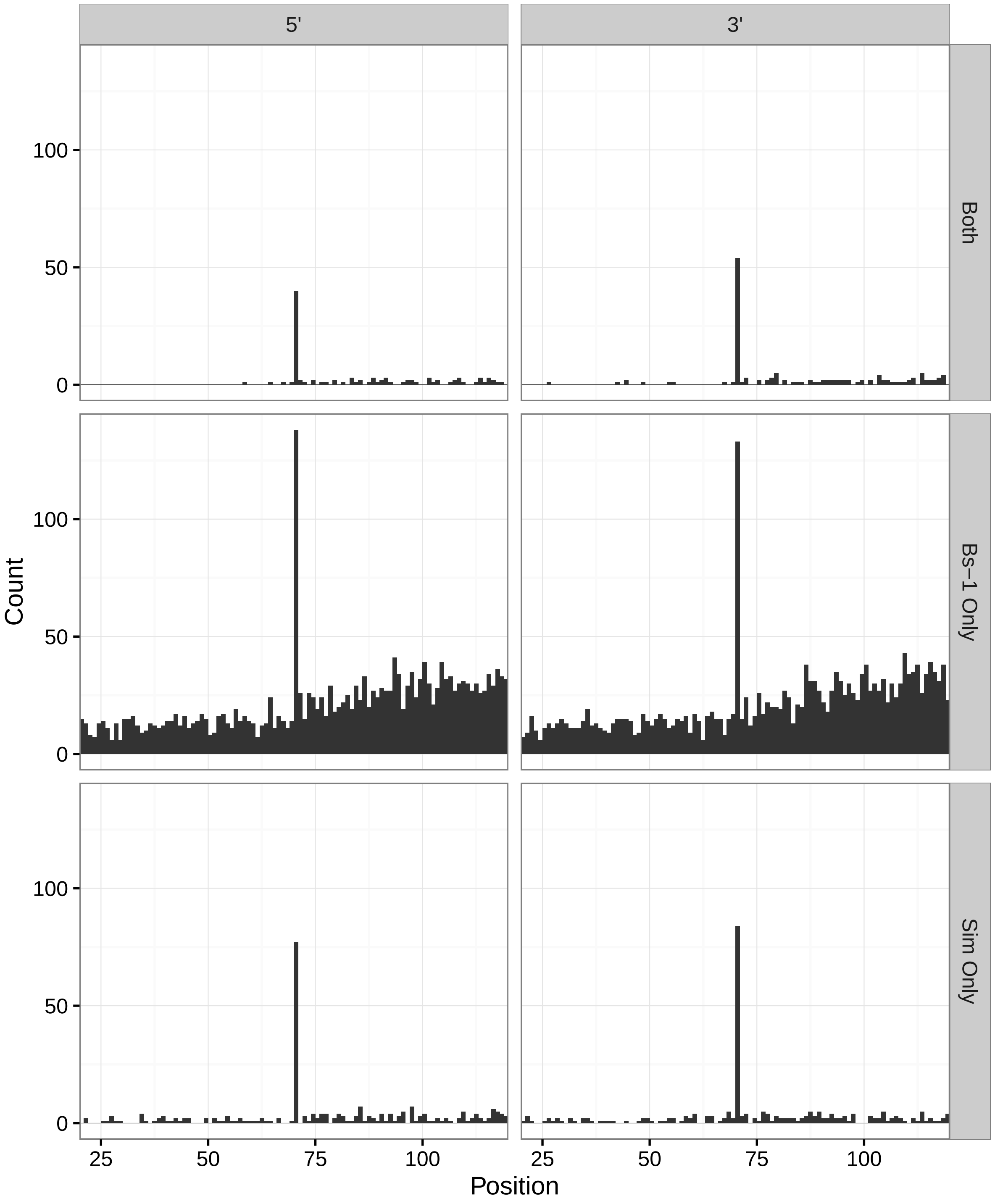

Figure S55: Distribution of positions for SNPs called against **Sim_soap_K69 scaffolds** using the actual *A. thaliana* Bs-1 reads or our simulated reads. The ‘Sim only’ row shows SNPs called only with the simulated reads, ‘Bs-1 only’ row exhibits SNPs unique to the Bs-1 alignments, while ‘Both’ row displays SNPs called individually with both Bs-1 and simulated reads. Scaffold coordinates were transformed into the contig coordinates.

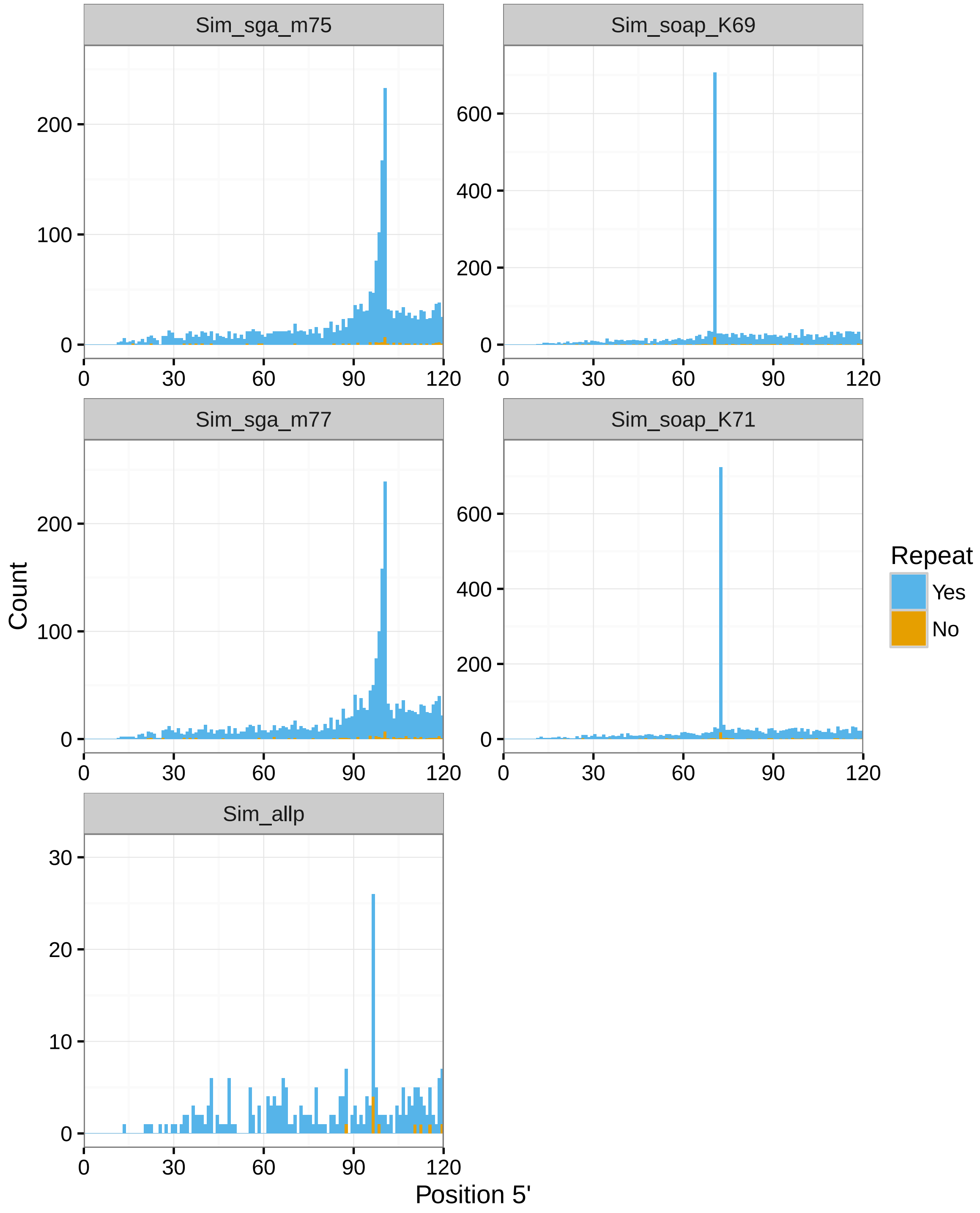

Figure S56: Distribution of SNP positions at the 5´ end of **contigs** in the simulated data set with repetitive element annotation. Colour indicates whether the SNPs are within repetitive sequences (blue) or not (orange). SNPs were called from the simulated read alignments. Repetitive elements included all sequences reported by RepeatMasker.

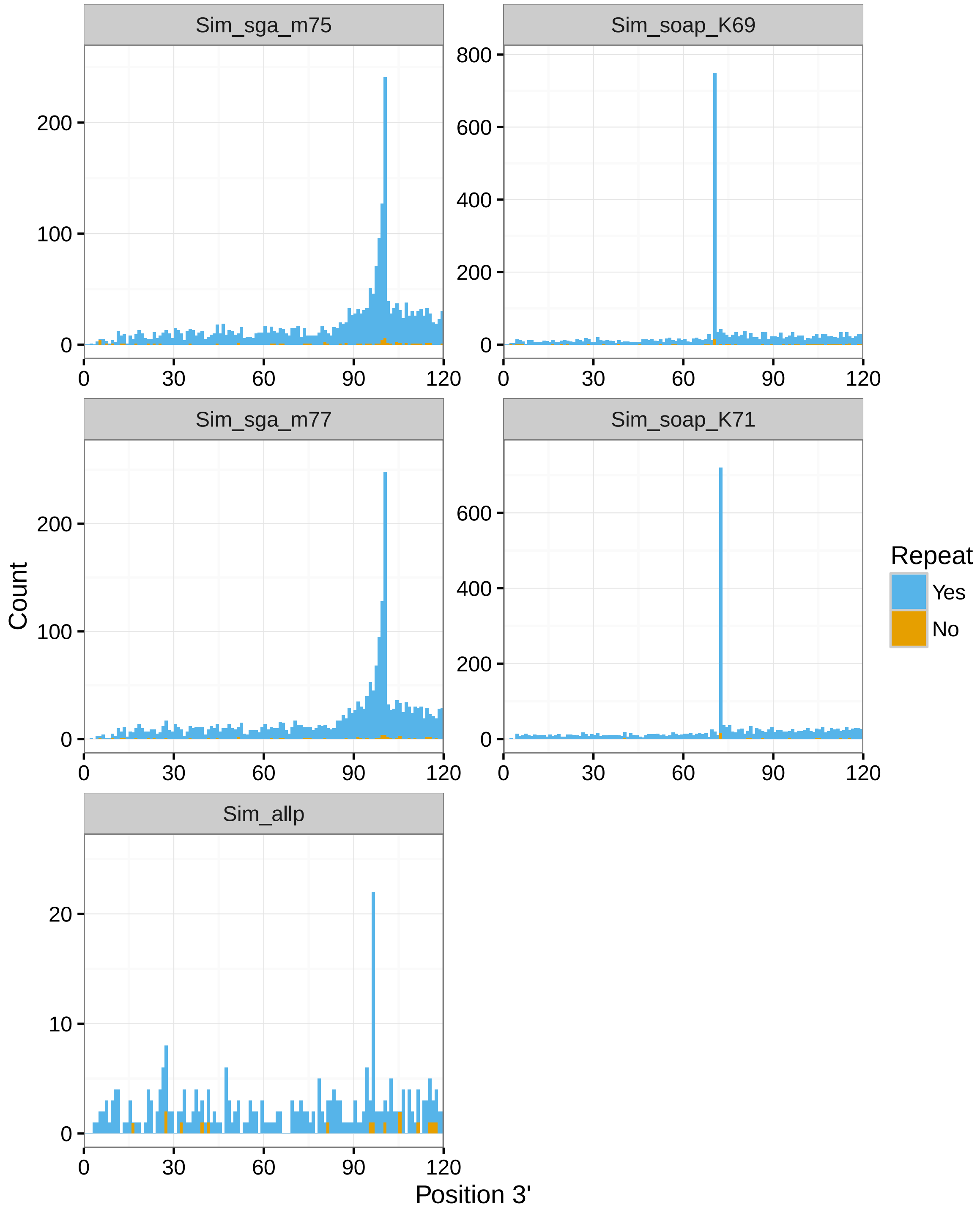

Figure S57: Distribution of SNP positions at the 3´ end of **contigs** in the simulated data set with repetitive element annotation. Colour indicates whether the SNPs are within repetitive sequences (blue) or not (orange). SNPs were called from the simulated read alignments. Repetitive elements included all sequences reported by RepeatMasker.

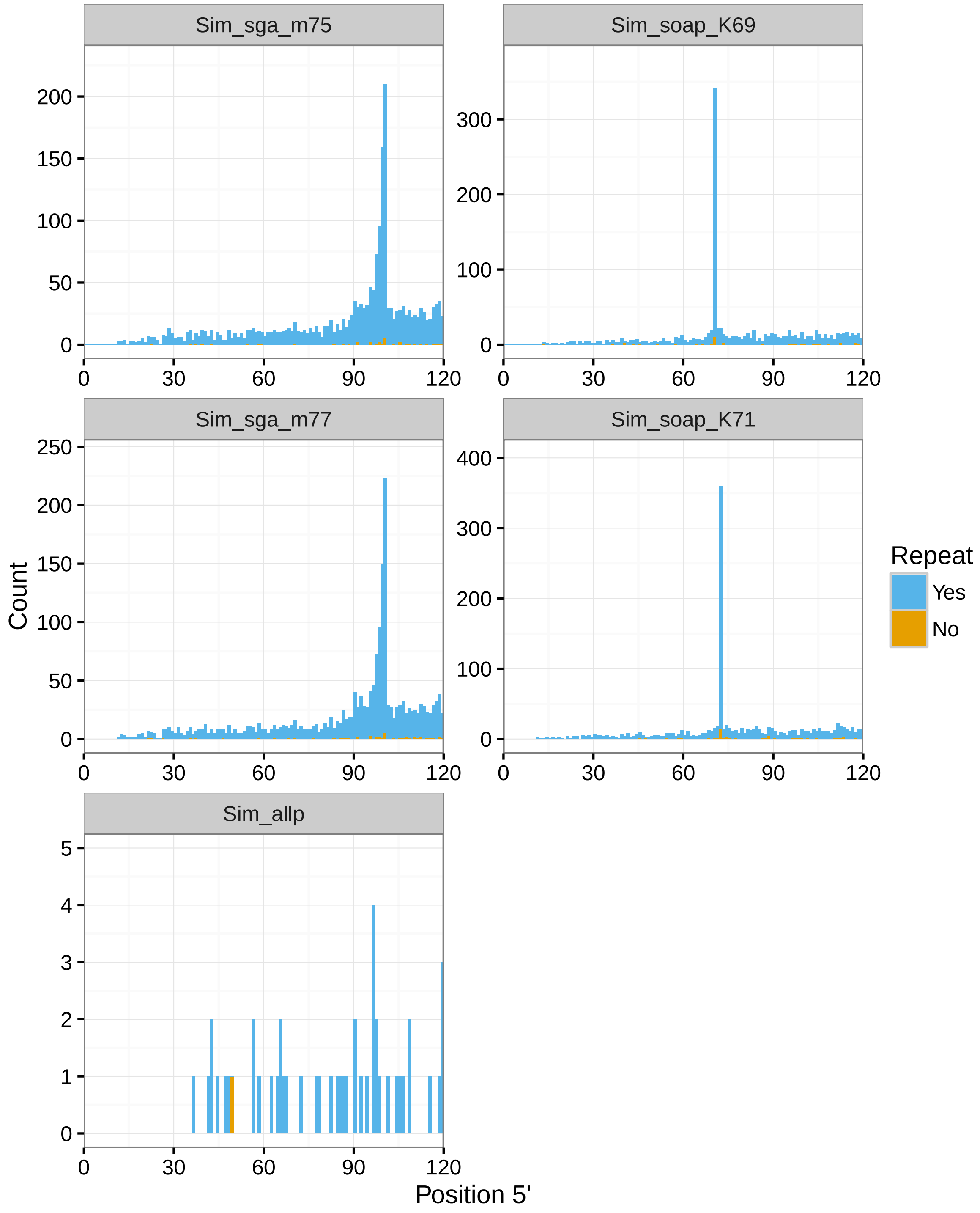

Figure S58: Distribution of SNP positions at the 5´ end of **scaffolds** in the simulated data set with repetitive element annotation. Colour indicates whether the SNPs are within repetitive sequences (blue) or not (orange). SNPs were called from the simulated read alignments. Repetitive elements included all sequences reported by RepeatMasker.

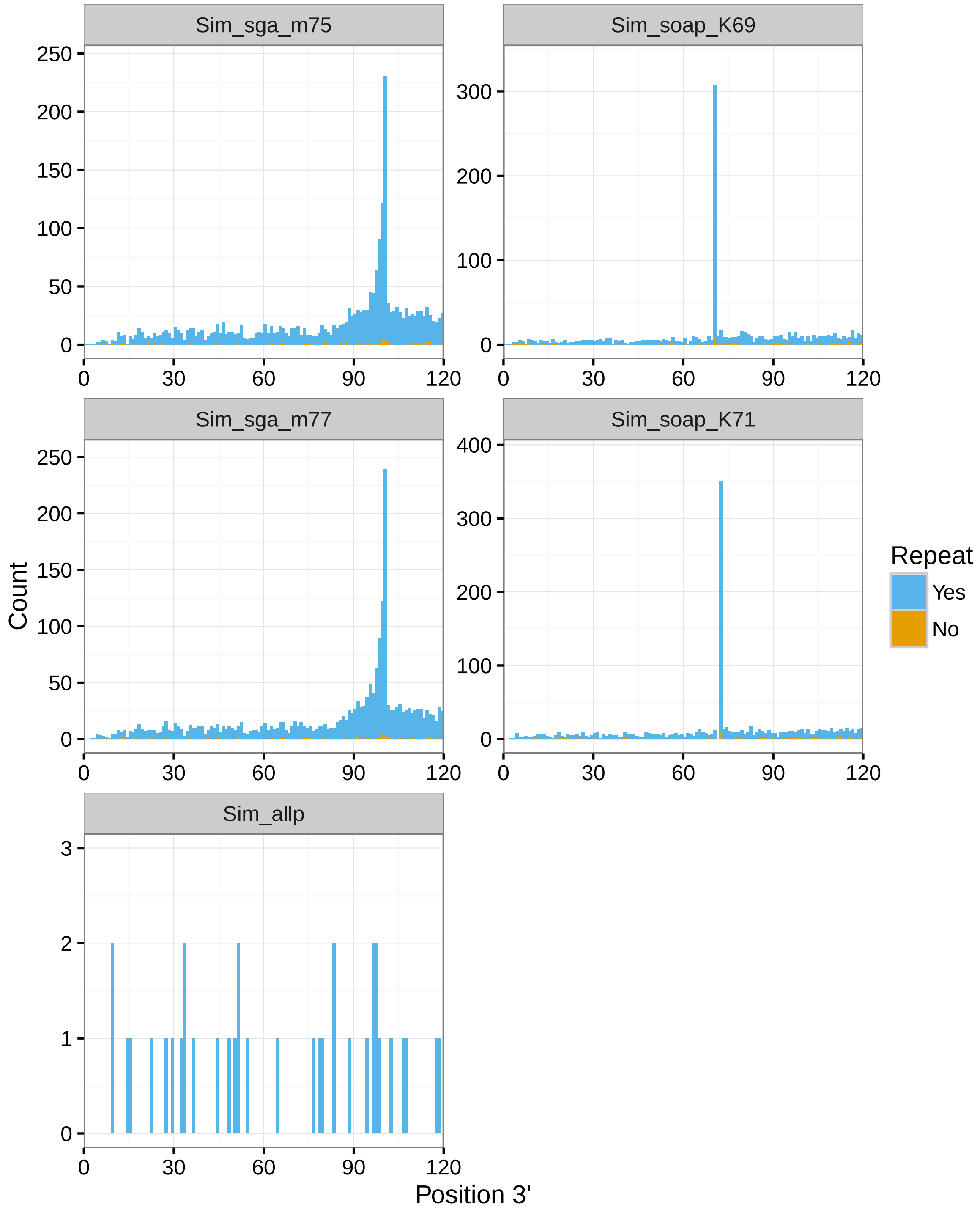

Figure S59: Distribution of SNP positions at the 3´ end of **scaffolds** in the simulated data set with repetitive element annotation. Colour indicates whether the SNPs are within repetitive sequences (blue) or not (orange). SNPs were called from the simulated read alignments. Repetitive elements included all sequences reported by RepeatMasker.

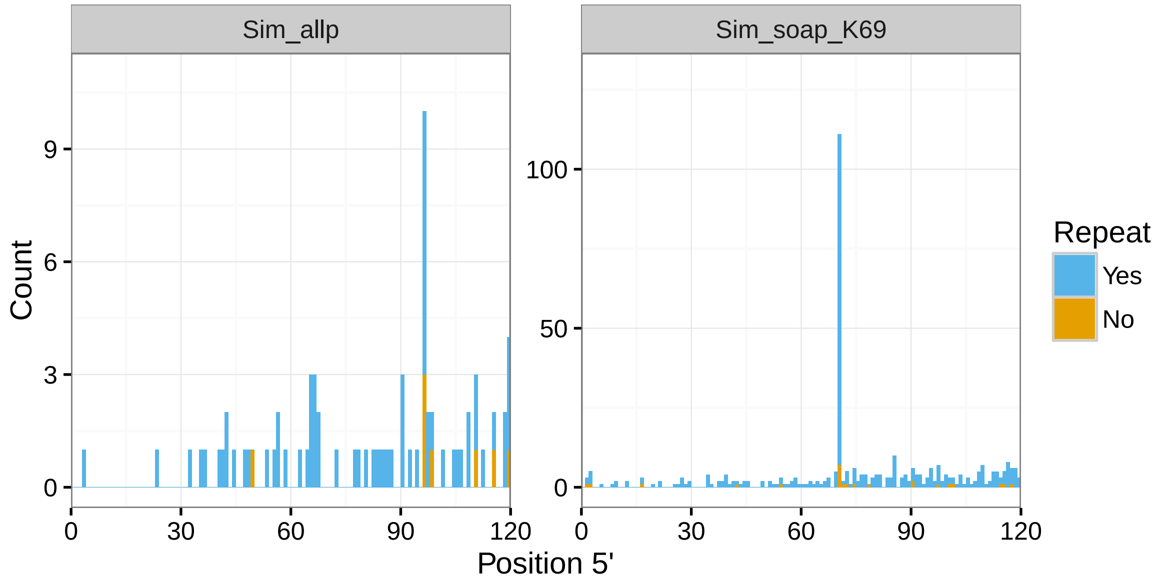

Figure S60: Distribution of SNP positions **transformed** from scaffold to contig coordinates at the 5´ end of contigs with repetitive element annotation. Colour indicates whether the SNPs are within repetitive sequences (blue) or not (orange). SNPs were called from the simulated read alignments. Repetitive elements included all sequences reported by RepeatMasker.

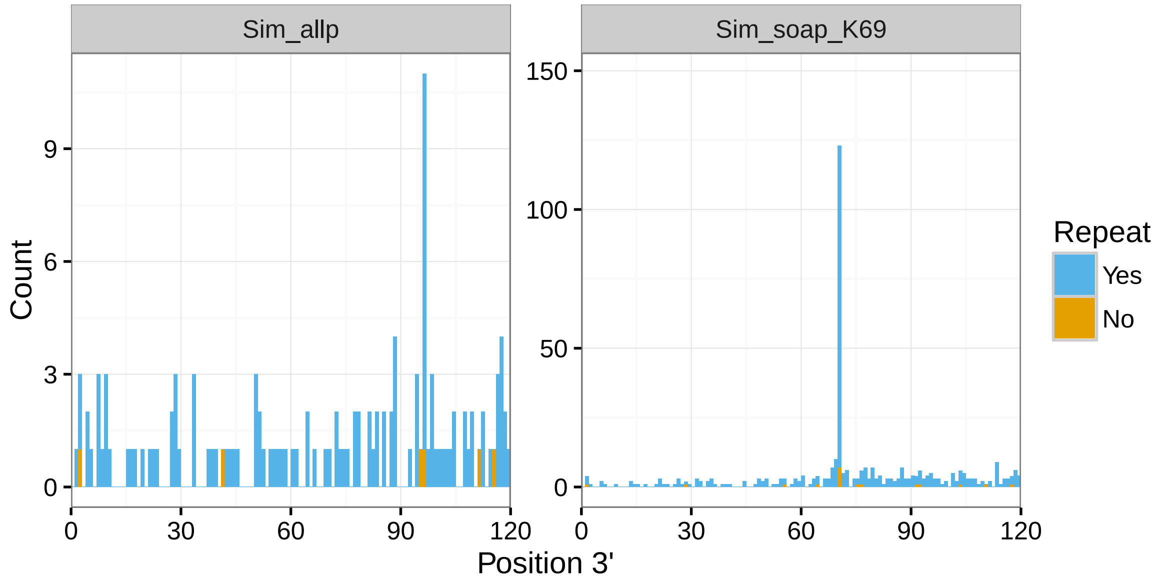

Figure S61: Distribution of SNP positions **transformed** from scaffold to contig coordinates at the 3´ end of contigs with repetitive element annotation. Colour indicates whether the SNPs are within repetitive sequences (blue) or not (orange). SNPs were called from the simulated read alignments. Repetitive elements included all sequences reported by RepeatMasker.

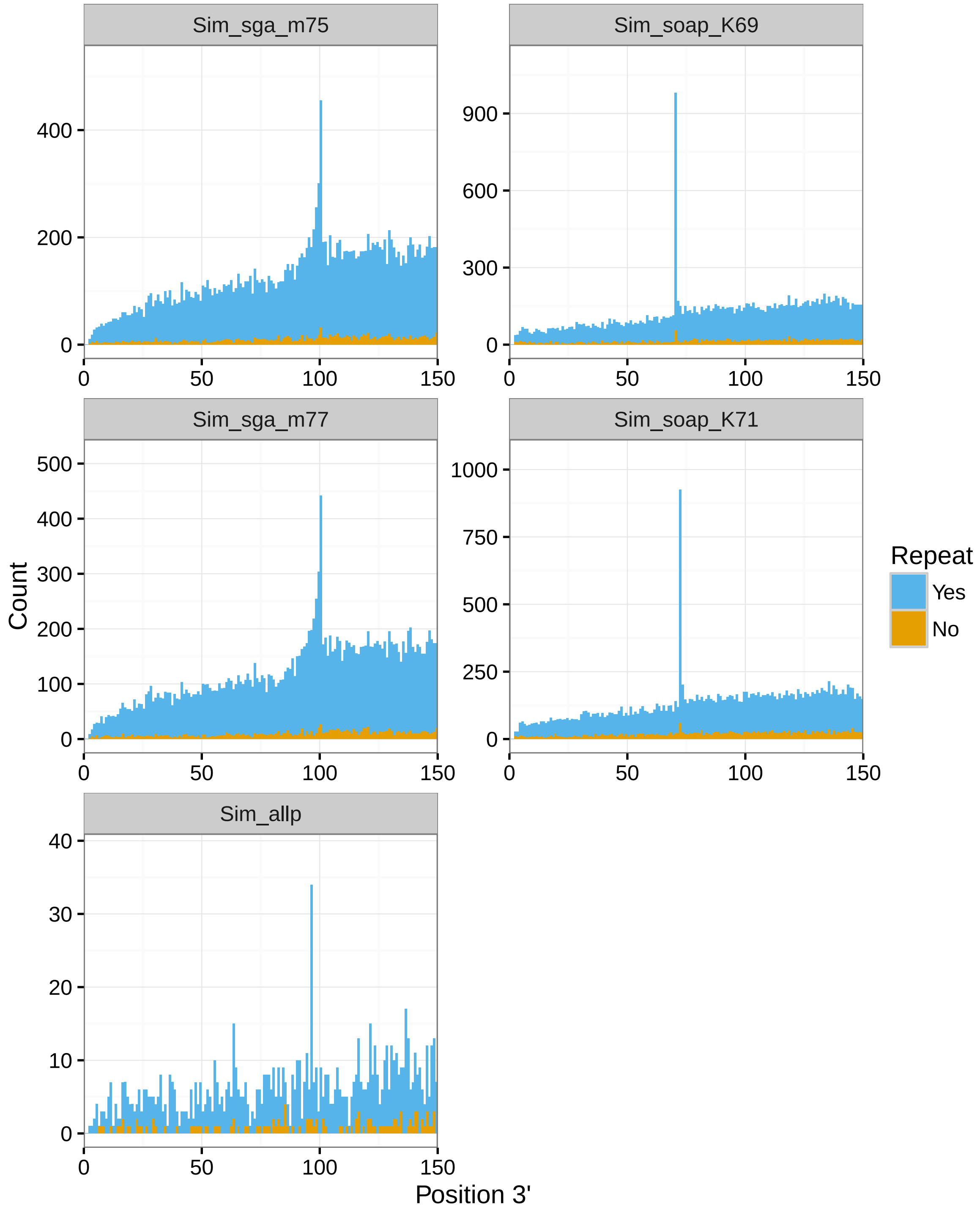

Figure S62: Distribution of SNP positions at the 3´ end of **contigs** in the Bs-1 data set with repetitive element annotation. Colour indicates whether the SNPs are within repetitive sequences (blue) or not (orange). SNPs were called from the Bs-1 read alignments. Repetitive elements included all sequences reported by RepeatMasker.

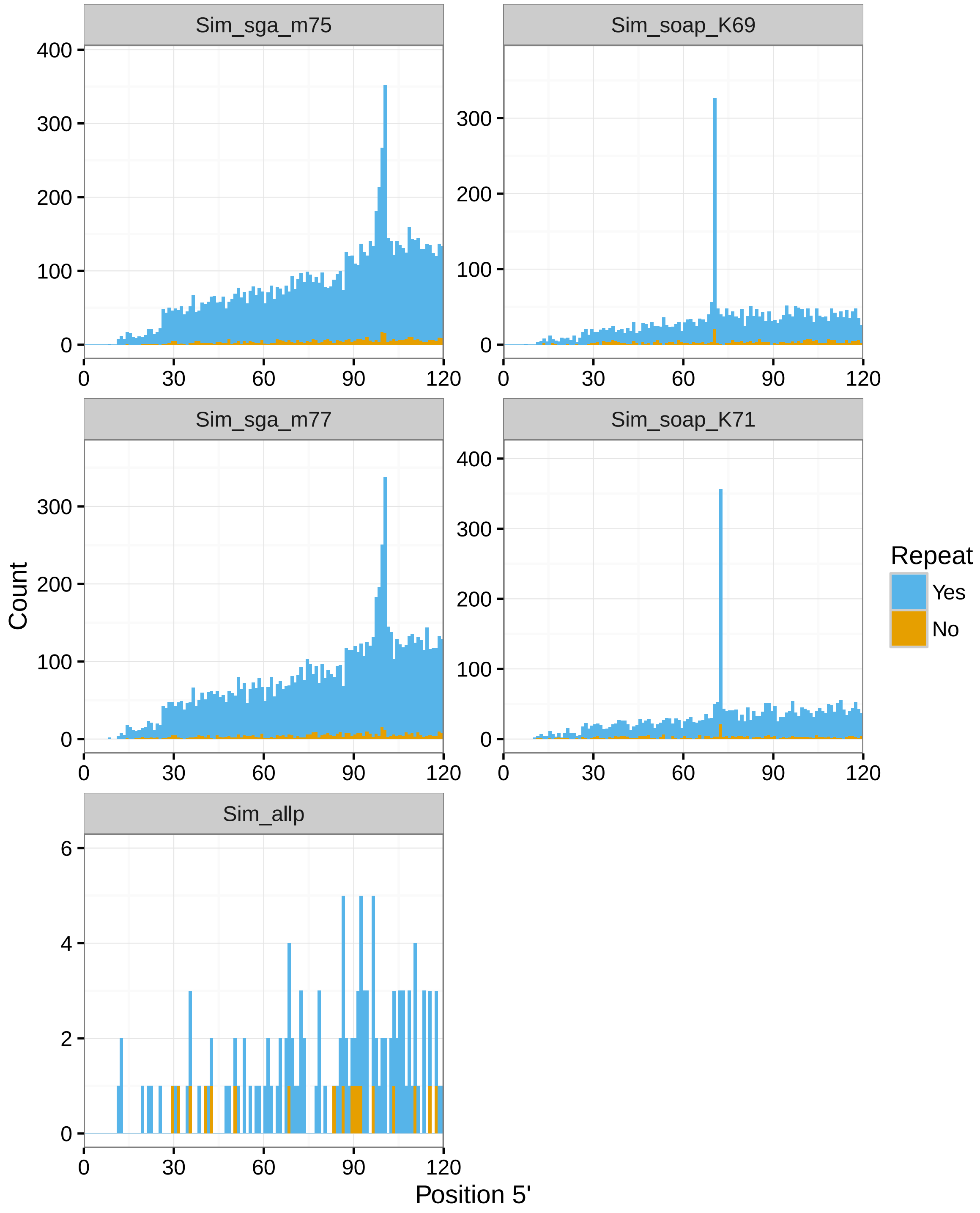

Figure S63: Distribution of SNP positions at the 5´ end of **scaffolds** in the Bs-1 data set with repetitive element annotation. Colour indicates whether the SNPs are within repetitive sequences (blue) or not (orange). SNPs were called from the Bs-1 read alignments. Repetitive elements included all sequences reported by RepeatMasker.

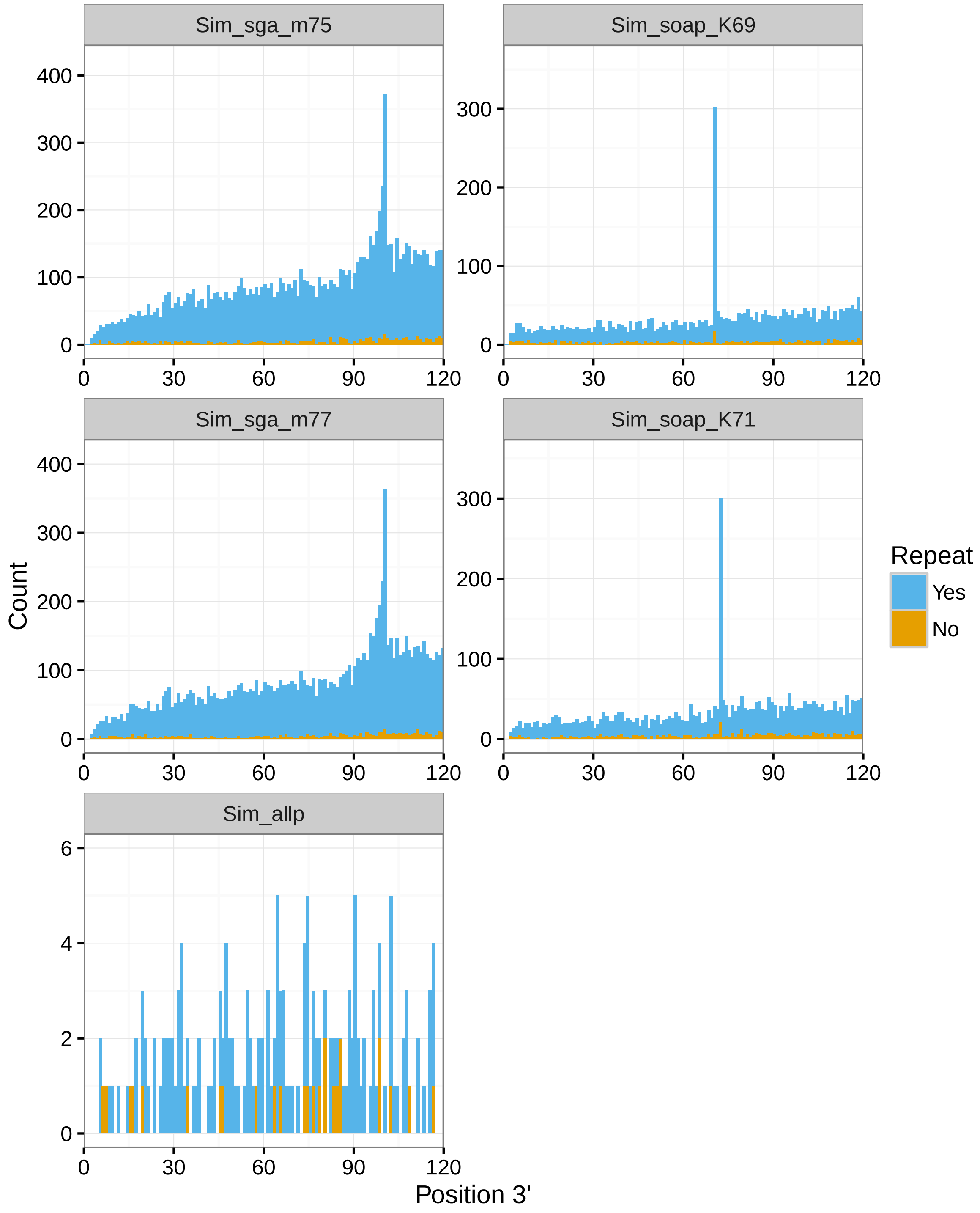

Figure S64: Distribution of SNP positions at the 3´ end of **scaffolds** in the Bs-1 data set with repetitive element annotation. Colour indicates whether the SNPs are within repetitive sequences (blue) or not (orange). SNPs were called from the Bs-1 read alignments. Repetitive elements included all sequences reported by RepeatMasker.

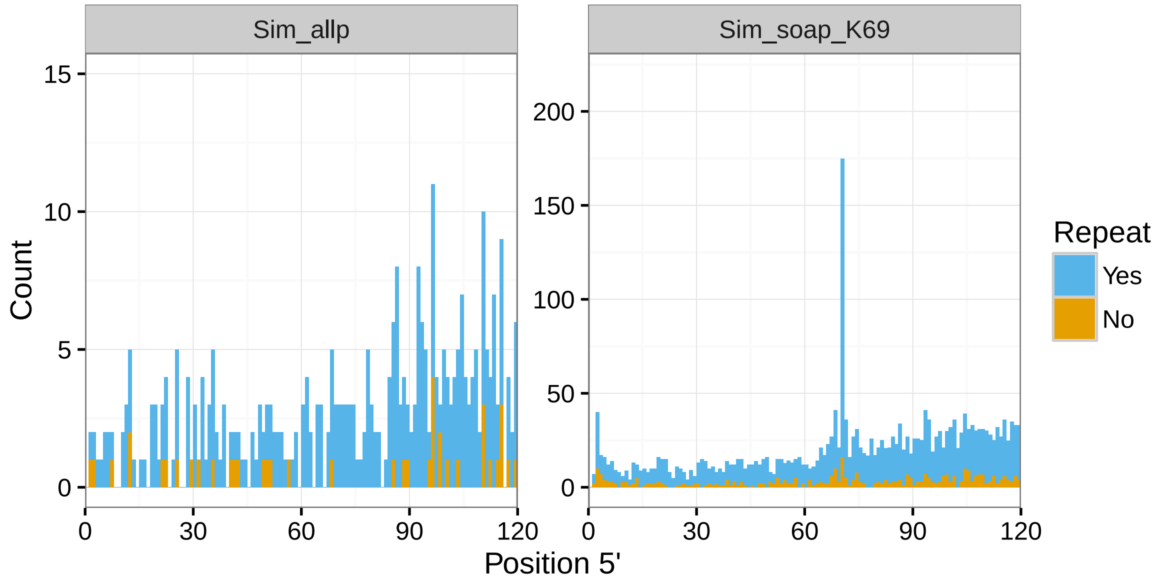

Figure S65: Distribution of SNP positions **transformed** from scaffold to contig coordinates at the 5´ end of contigs with repetitive element annotation. Colour indicates whether the SNPs are within repetitive sequences (blue) or not (orange). SNPs were called from the Bs-1 read alignments. Repetitive elements included all sequences reported by RepeatMasker.

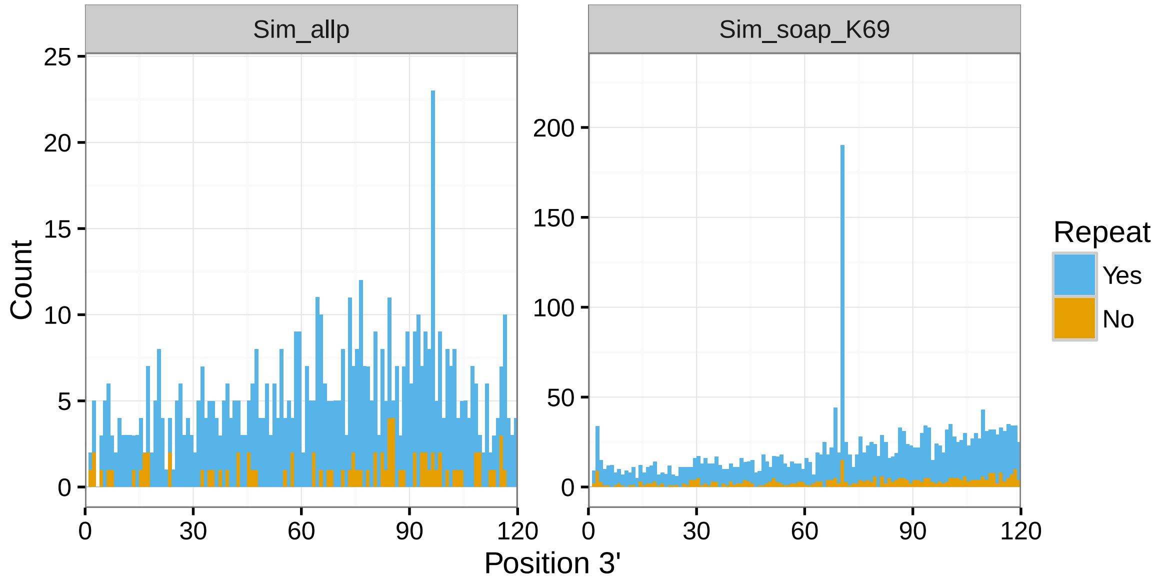

Figure S66: Distribution of SNP positions **transformed** from scaffold to contig coordinates at the 3´ end of contigs with repetitive element annotation. Colour indicates whether the SNPs are within repetitive sequences (blue) or not (orange). SNPs were called from the Bs-1 read alignments. Repetitive elements included all sequences reported by RepeatMasker.

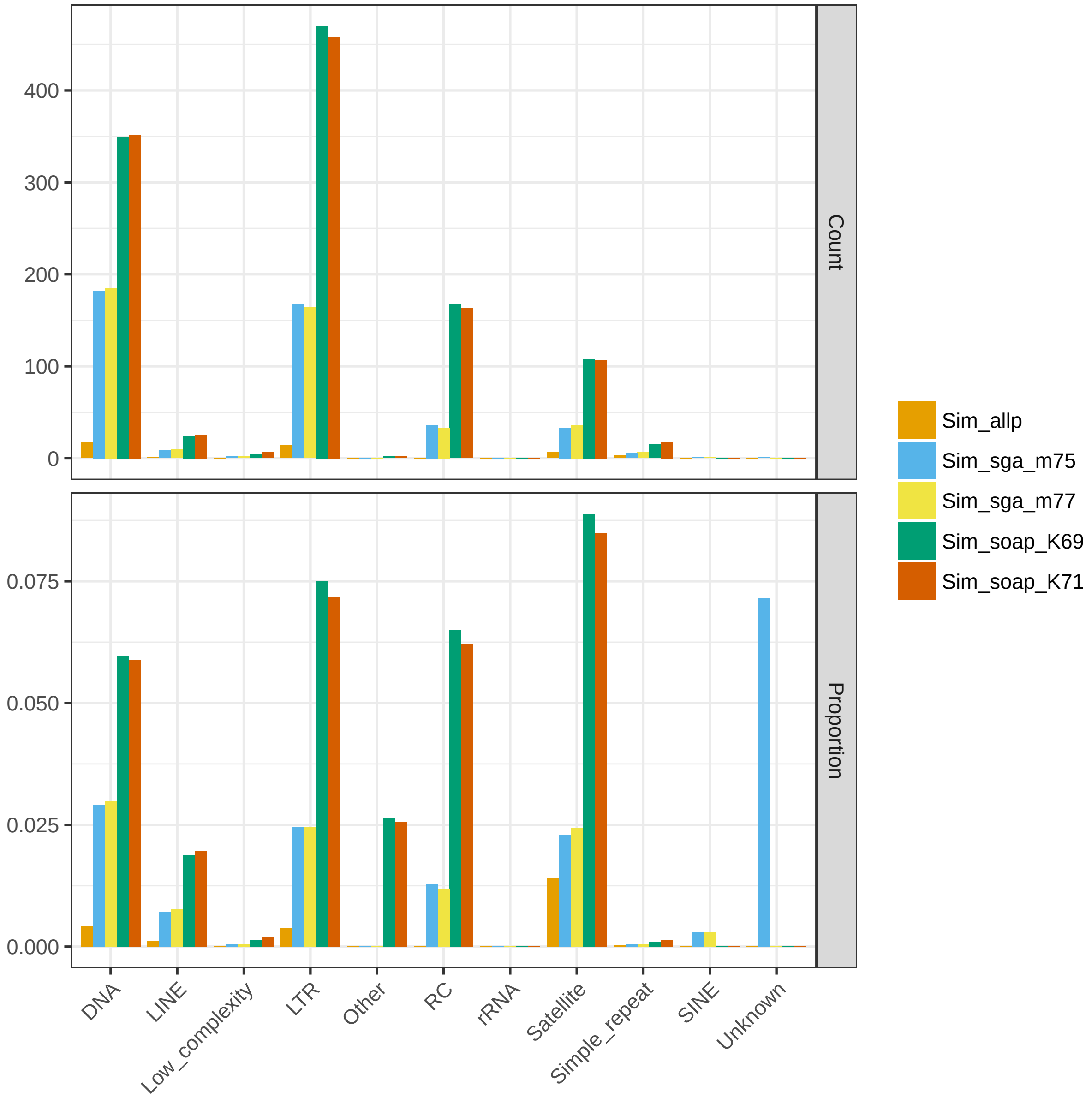

Figure S67: Distribution of SNPs at position k in the simulated contigs by repetitive element family. The top panel shows the total number of position k SNPs within each family. The lower panel shows the proportion of repetitive element sequences that contain a SNP at position k. The proprotion of SNPs in the Unknown family was high for Sim_sga_m75 due to the low total number of such sequences in the assembly, i.e. a single SNP occurrence would already yield a high proportion.

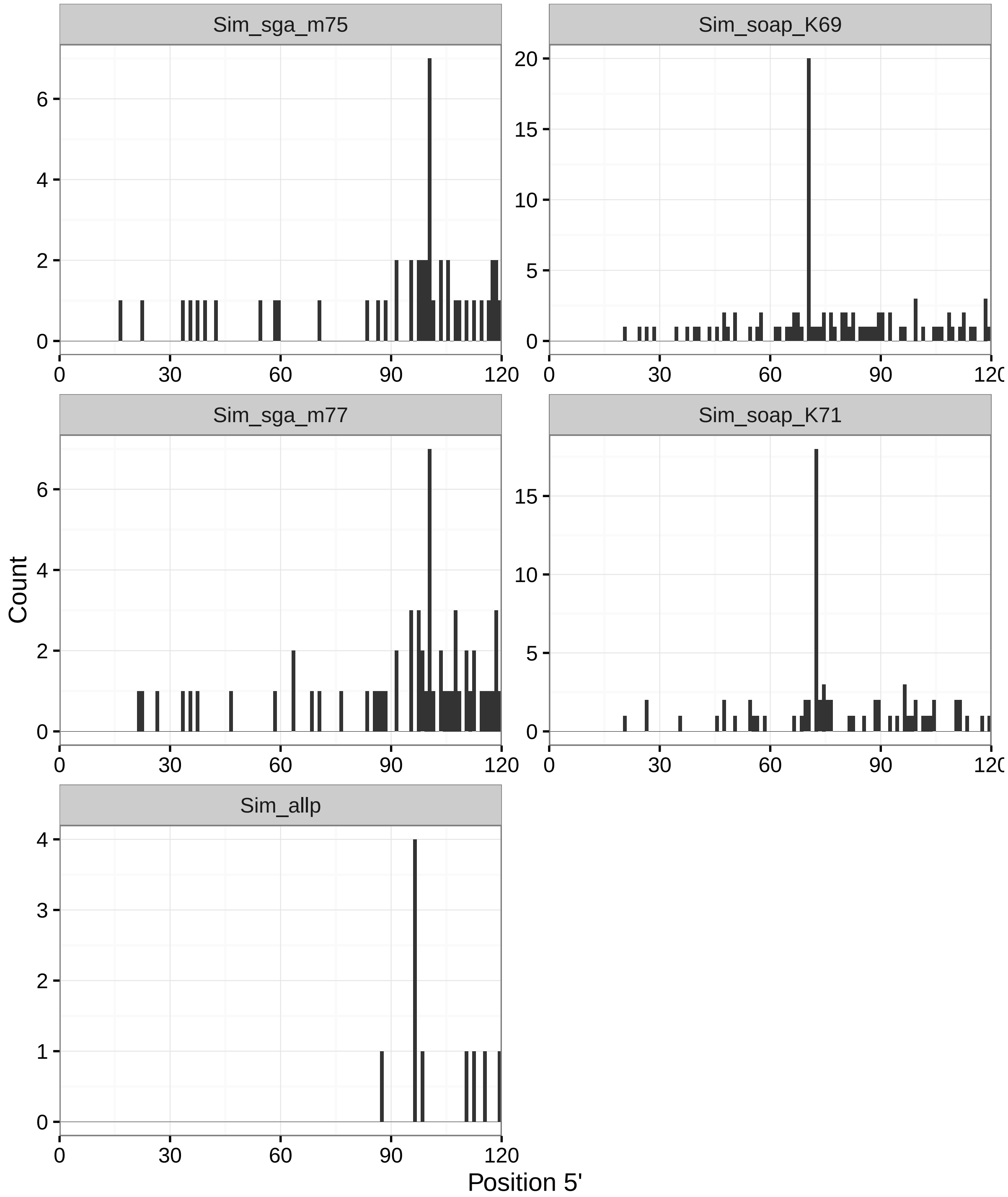

Figure S68: Distribution of SNP positions at the 5´ end of **contigs** in the simulated data set after repetitive element filtering. SNPs were called from the simulated read alignments and SNPs located in the annotated repetitive elements were removed.

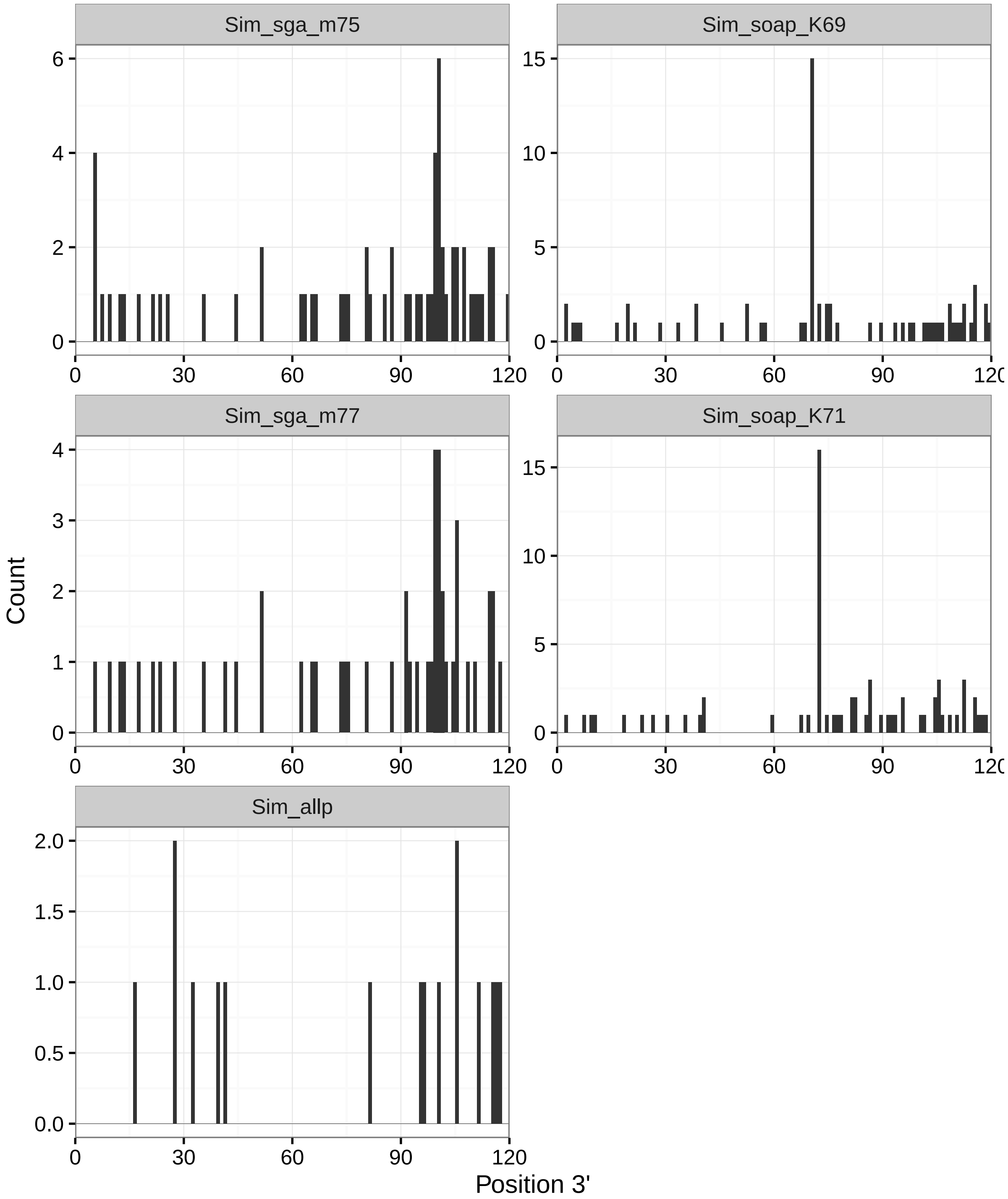

Figure S69: Distribution of SNP positions at the 3´ end of **contigs** in the simulated data set after repetitive element filtering. SNPs were called from the simulated read alignments and SNPs located in the annotated repetitive elements were removed.

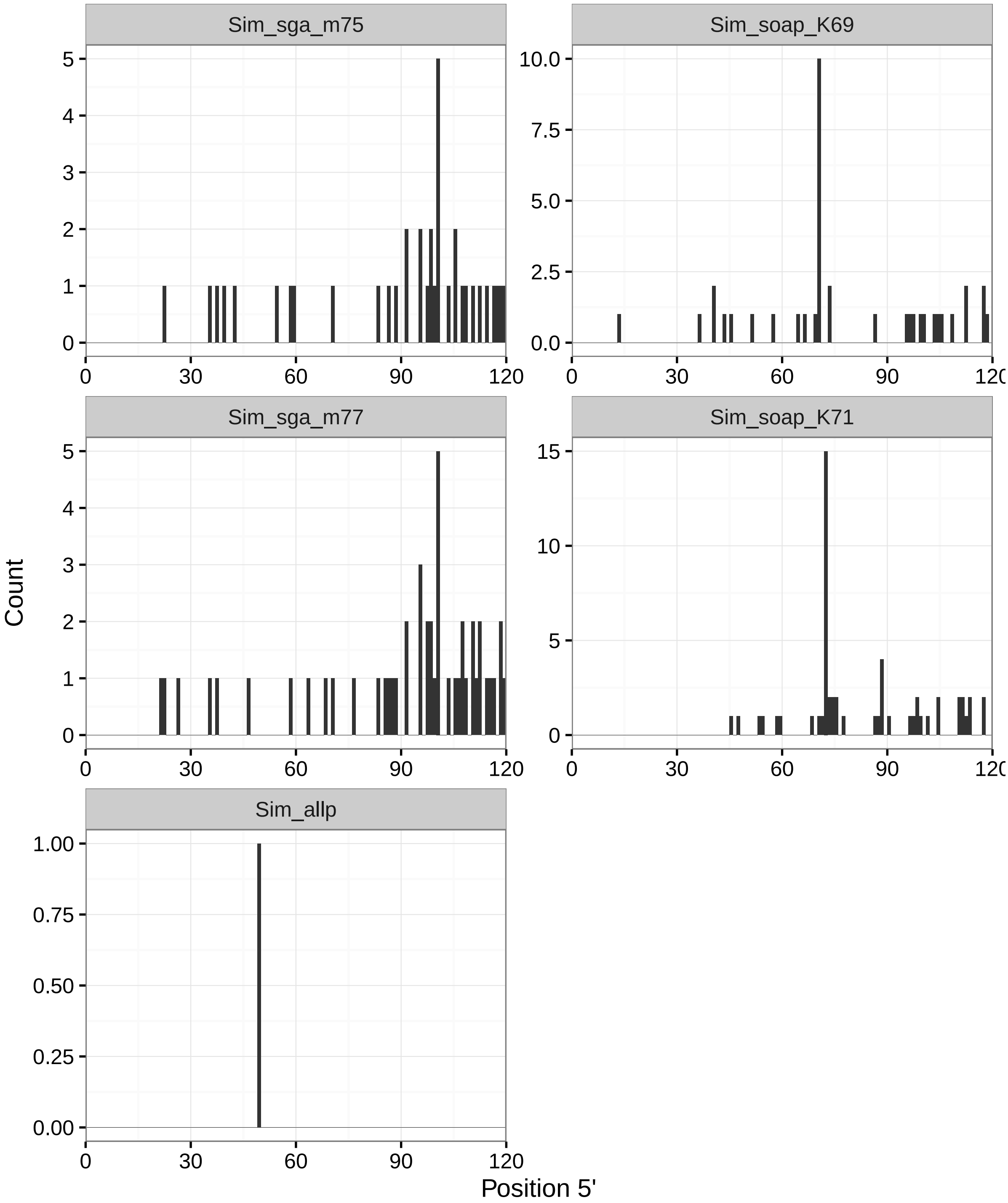

Figure S70: Distribution of SNP positions at the 5´ end of **scaffolds** in the simulated data set after repetitive element filtering. SNPs were called from the simulated read alignments and SNPs located in the annotated repetitive elements were removed.

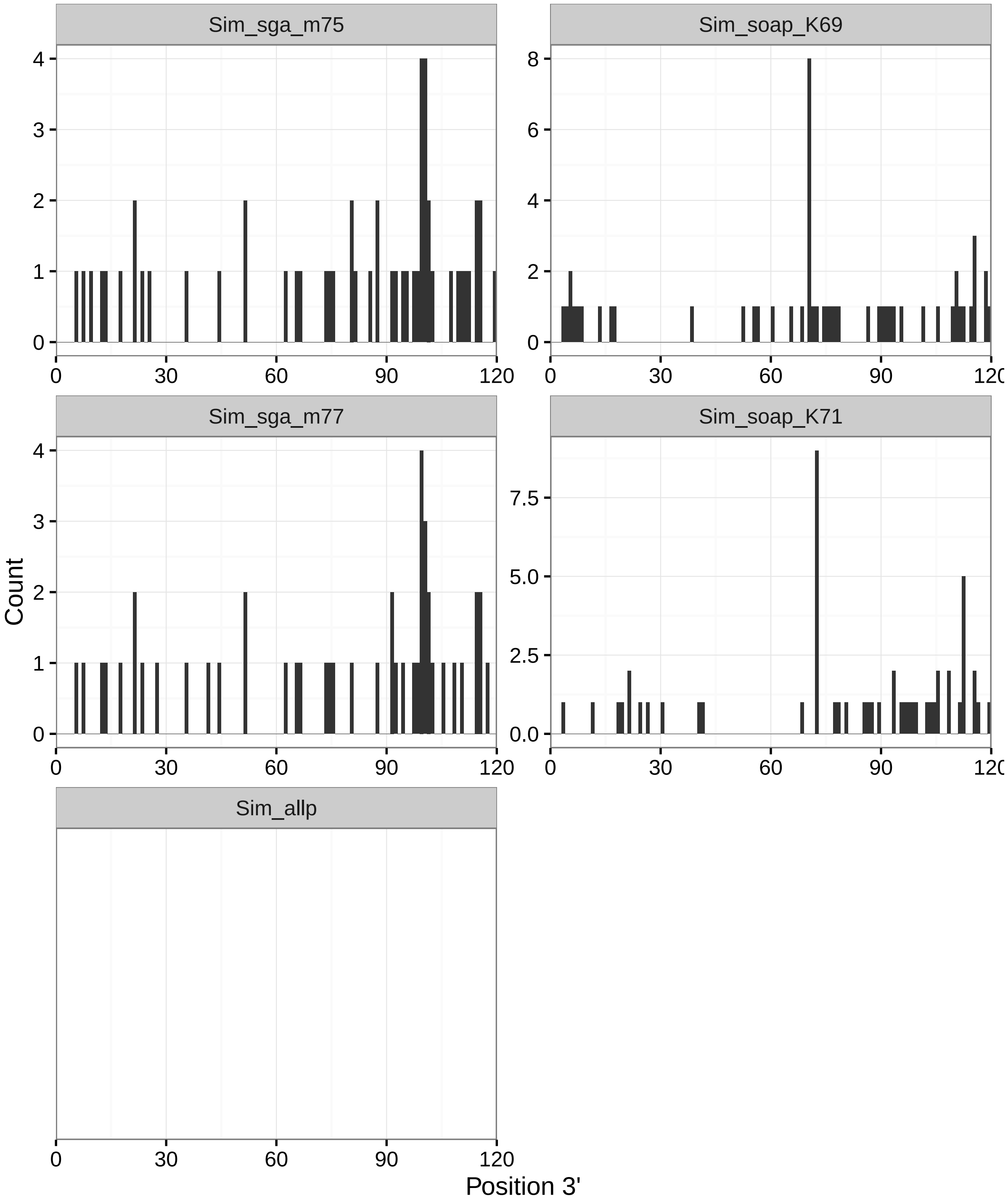

Figure S71: Distribution of SNP positions at the 3´ end of **scaffolds** in the simulated data set after repetitive element filtering. SNPs were called from the simulated read alignments and SNPs located in the annotated repetitive elements were removed.

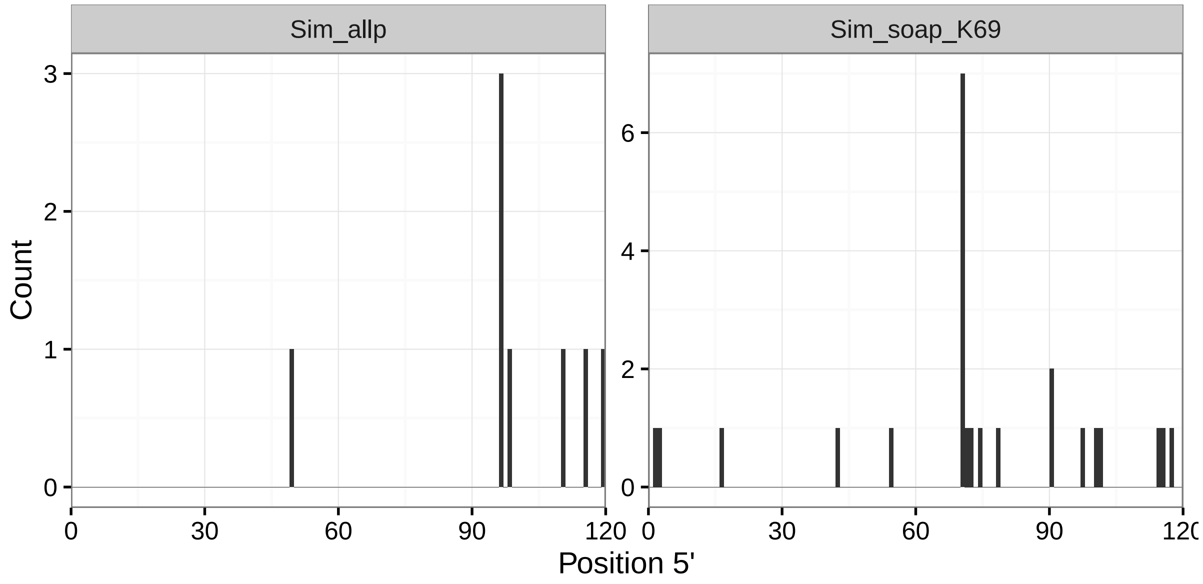

Figure S72: Distribution of SNP positions **transformed** from scaffold to contig coordinates at the 5´ end of contigs after repetitive element filtering. SNPs were called from the simulated read alignments and SNPs located in the annotated repetitive elements were removed.

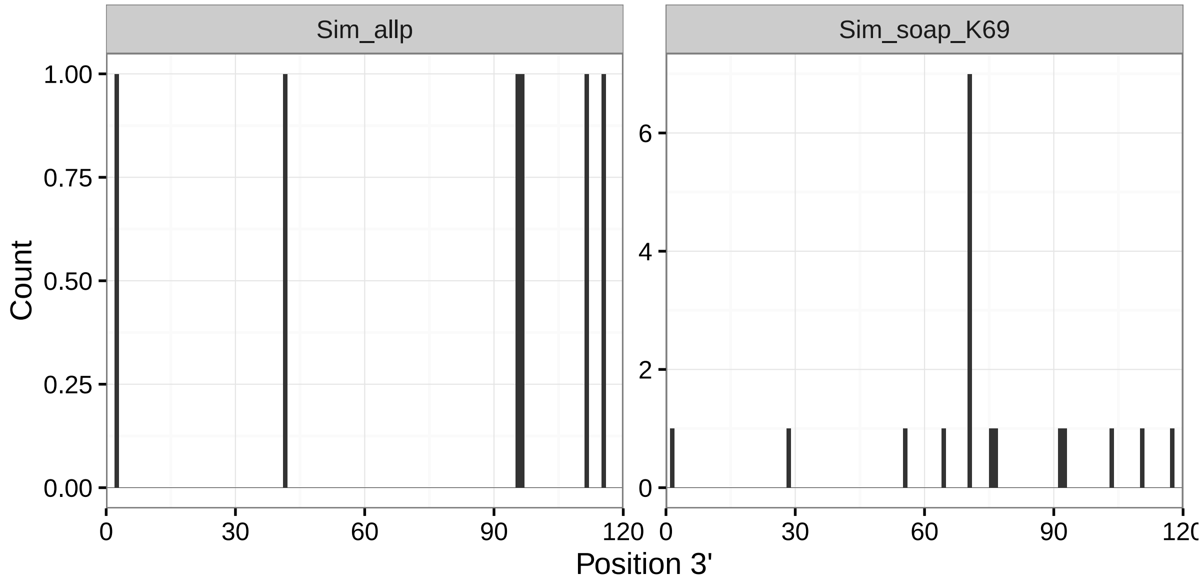

Figure S73: Distribution of SNP positions **transformed** from scaffold to contig coordinates at the 3´ end of contigs after repetitive element filtering. SNPs were called from the simulated read alignments and SNPs located in the annotated repetitive elements were removed.

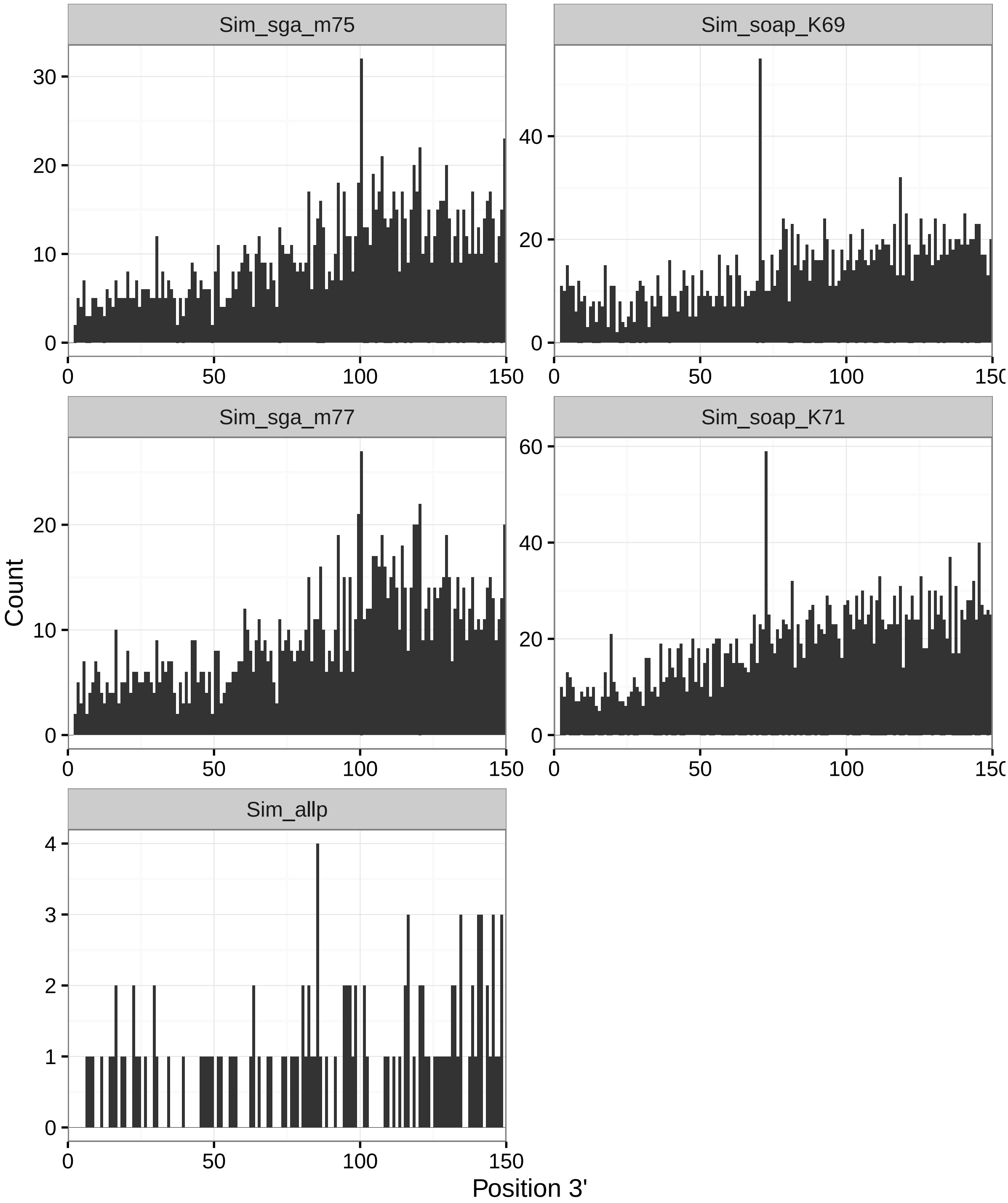

Figure S74: Distribution of SNP positions at the 3´ end of **contigs** in the Bs-1 data set after repetitive element filtering. SNPs were called from the Bs-1 read alignments and SNPs located in the annotated repetitive elements were removed.

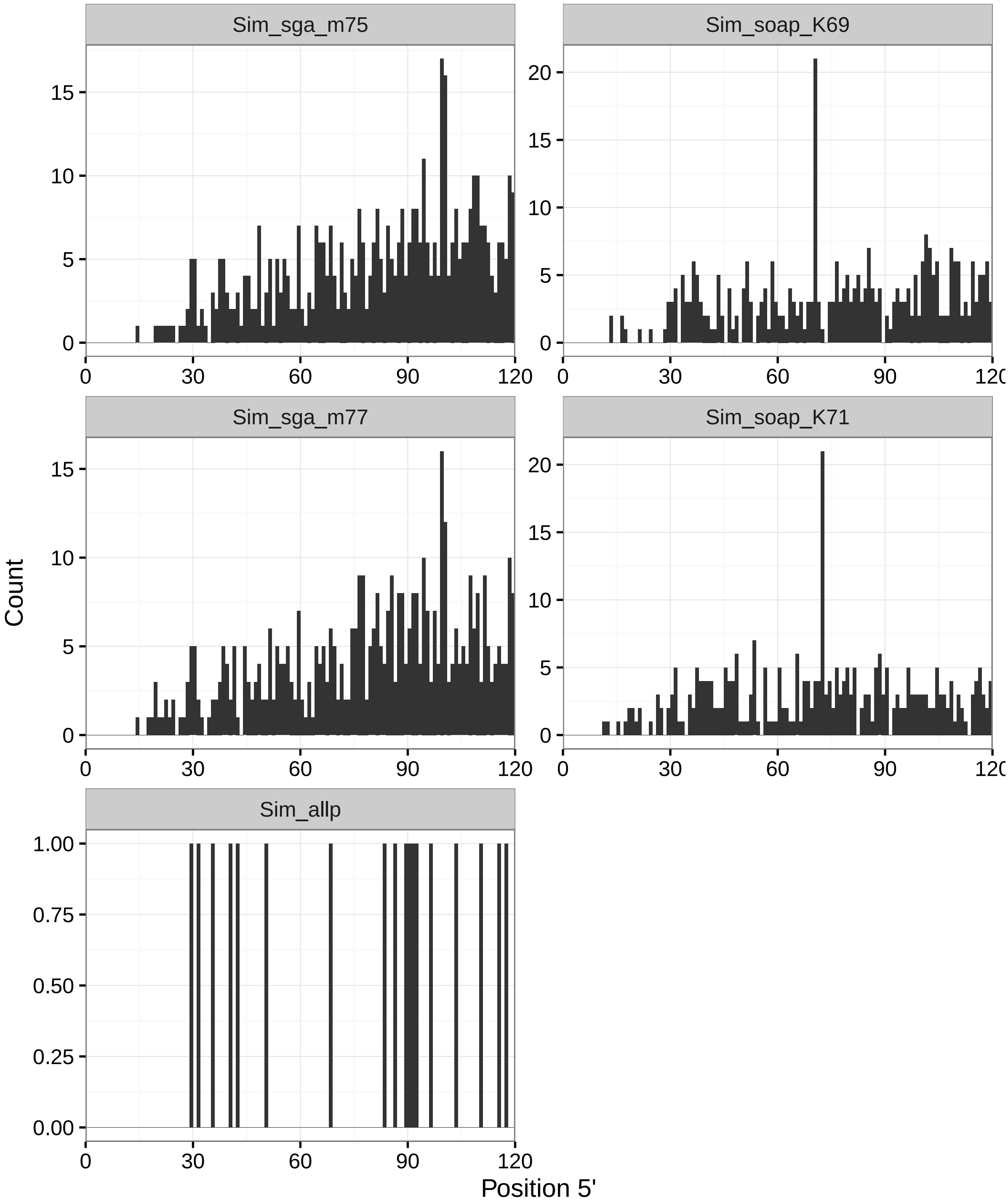

Figure S75: Distribution of SNP positions at the 5´ end of **scaffolds** in the Bs-1 data set after repetitive element filtering. SNPs were called from the Bs-1 read alignments and SNPs located in the annotated repetitive elements were removed.

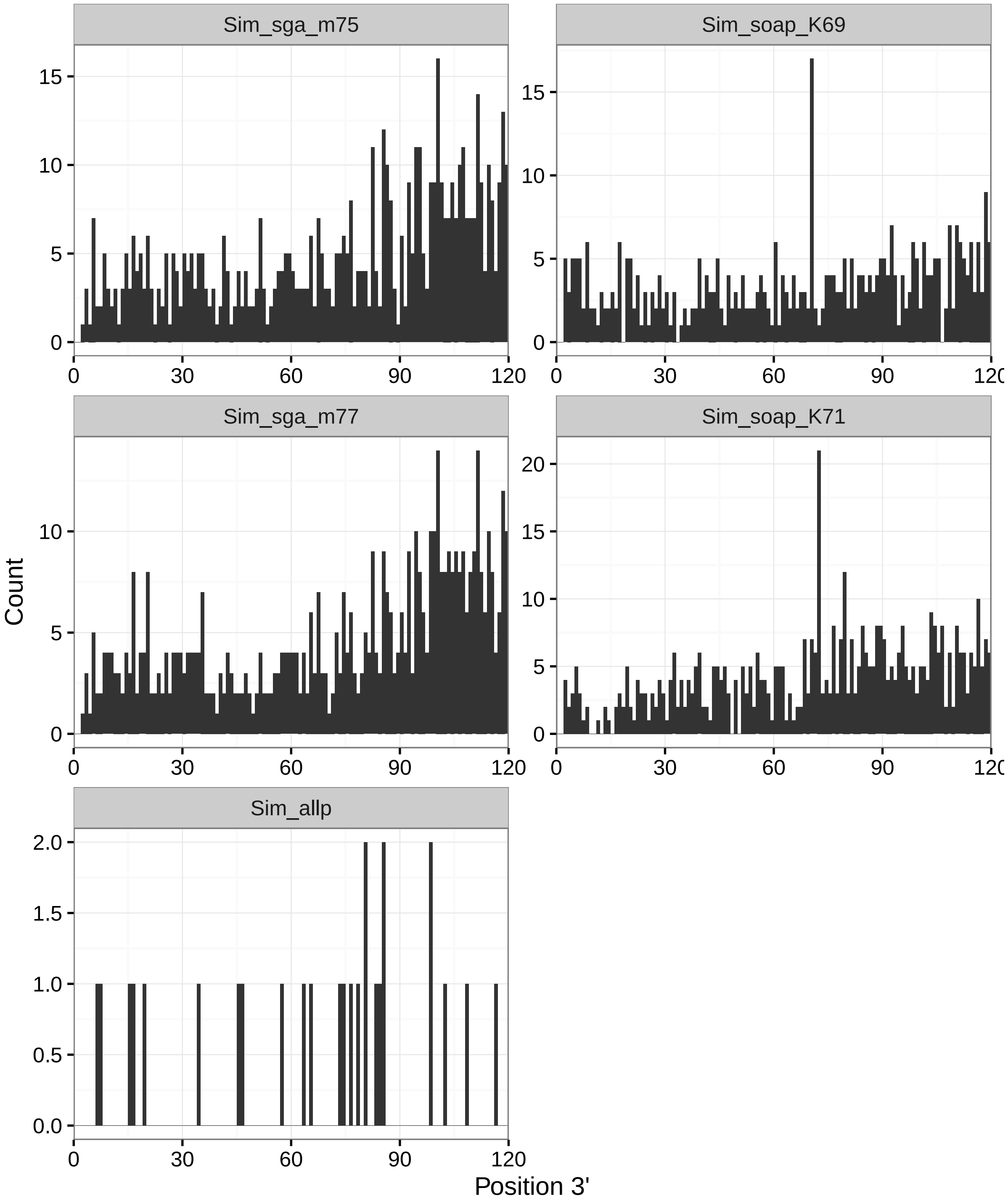

Figure S76: Distribution of SNP positions at the 3´ end of **scaffolds** in the Bs-1 data set after repetitive element filtering. SNPs were called from the Bs-1 read alignments and SNPs located in the annotated repetitive elements were removed.

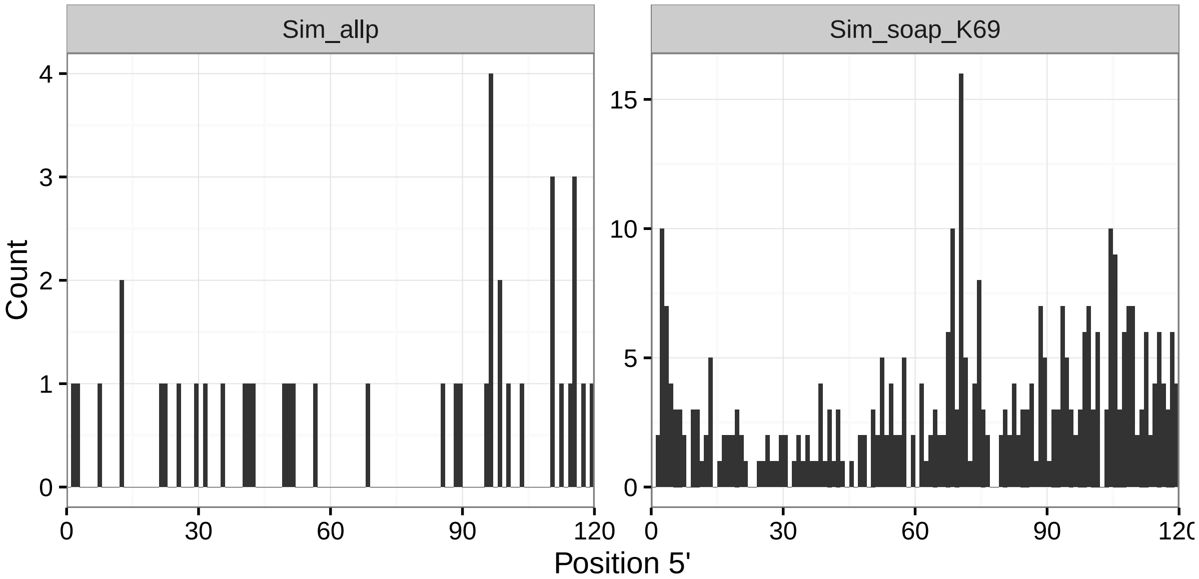

Figure S77: Distribution of SNP positions **transformed** from scaffold to contig coordinates at the 5´ end of contigs after repetitive element filtering. SNPs were called from the Bs-1 read alignments and SNPs located in the annotated repetitive elements were removed.

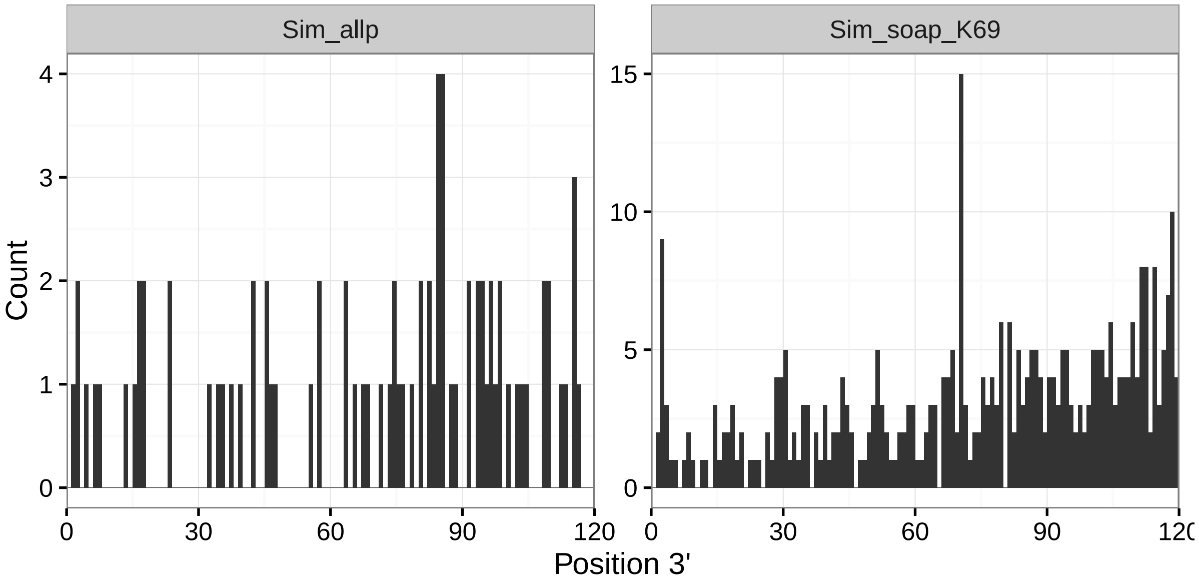

Figure S78: Distribution of SNP positions **transformed** from scaffold to contig coordinates at the 3´ end of contigs after repetitive element filtering. SNPs were called from the Bs-1 read alignments and SNPs located in the annotated repetitive elements were removed.

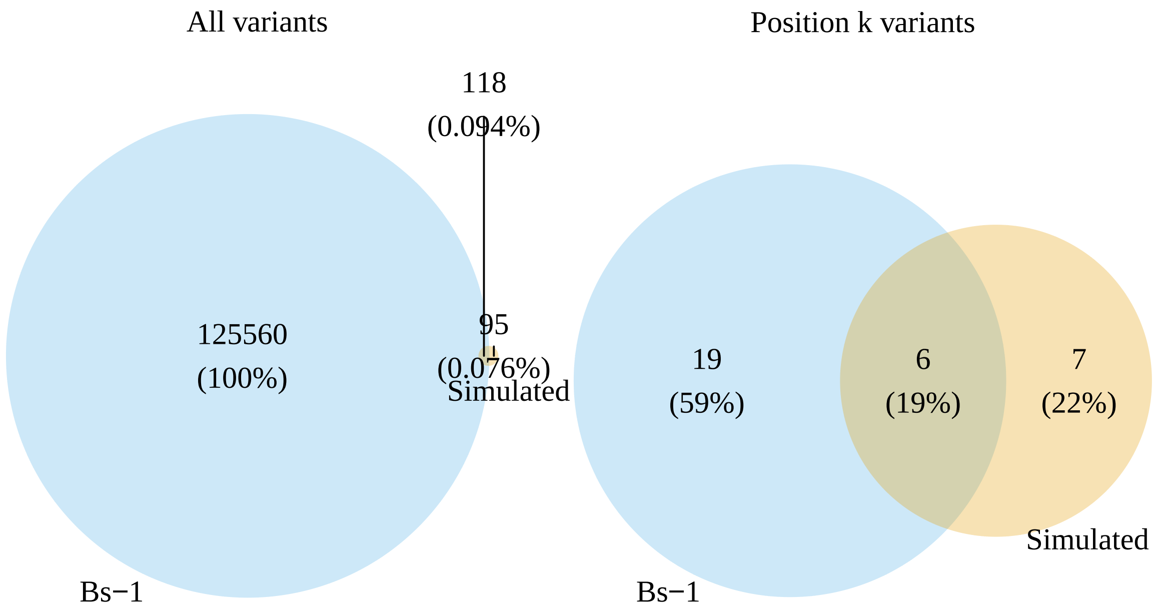

Figure S79: Intersection between SNPs called against **Sim_soap_K69 scaffolds** with the actual *A. thaliana* Bs-1 reads or our simulated reads after repetitive element filtering. Scaffold coordinates were transformed into the contig coordinates.

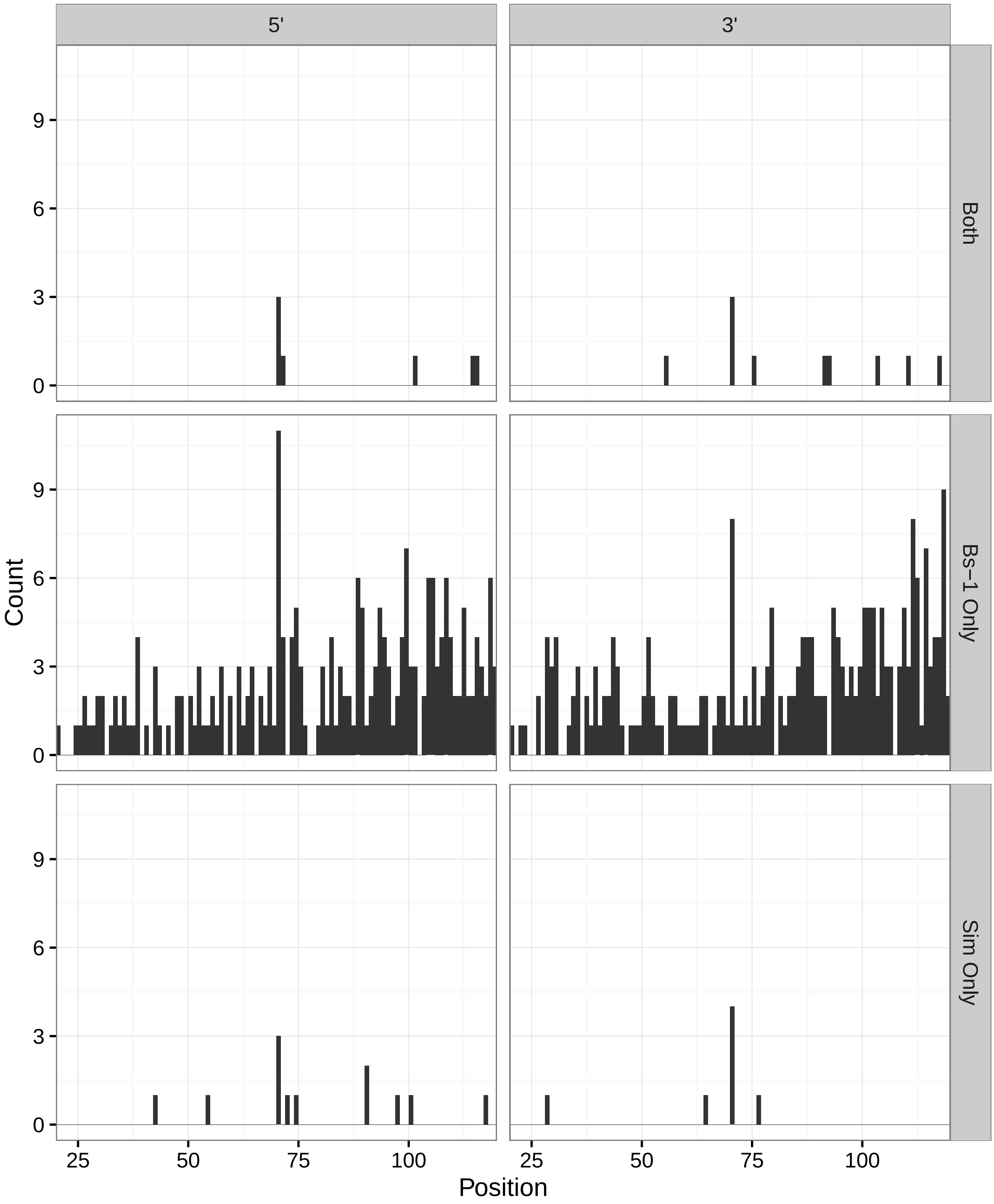

Figure S80: Distribution of positions for SNPs called against **Sim_soap_K69 scaffolds** after coordinate transformation and repetitive element filtering.
